# Iron(iv) alkyl complexes: electronic structure contributions to Fe–C bond homolysis and migration reactions that form N–C bonds from N_2_†

**DOI:** 10.1039/d3sc05939a

**Published:** 2024-01-24

**Authors:** Samuel M. Bhutto, Reagan X. Hooper, Sean F. McWilliams, Brandon Q. Mercado, Patrick L. Holland

**Affiliations:** a Department of Chemistry, Yale University New Haven Connecticut 06520 USA patrick.holland@yale.edu

## Abstract

High-valent iron alkyl complexes are rare, as they are prone to Fe–C bond homolysis. Here, we describe an unusual way to access formally iron(iv) alkyl complexes through double silylation of iron(i) alkyl dinitrogen complexes to form an NNSi_2_ group. Spectroscopically validated computations show that the disilylehydrazido(2−) ligand stabilizes the formal iron(iv) oxidation state through a strongly covalent Fe–N π-interaction, in which one π-bond fits an “inverted field” description. This means that the two bonding electrons are localized more on the metal than the ligand, and thus an iron(ii) resonance structure is a significant contributor, similar to the previously-reported phenyl analogue. However, in contrast to the phenyl complex which has an *S* = 1 ground state, the ground state of the alkyl complex is *S* = 2, which places one electron in the π* orbital, leading to longer and weaker Fe–N bonds. The reactivity of these hydrazido(2−) complexes is dependent on the steric and electronic properties of the specific alkyl group. When the alkyl group is the bulky trimethylsilylmethyl, the formally iron(iv) species is stable at room temperature and no migration of the alkyl ligand is observed. However, the analogous complex with the smaller methyl ligand does indeed undergo migration of the carbon-based ligand to the NNSi_2_ group to form a new N–C bond. This migration is followed by isomerization of the hydrazido ligand, and the product exists as two isomers that have distinct η^1^ and η^2^ binding of the hydrazido group. Lastly, when the alkyl group is benzyl, the Fe–C bond homolyzes to give a three-coordinate hydrazido(2−) complex which is likely due to the greater stability of a benzyl radical compared to that for methyl or trimethylsilylmethyl. These studies demonstrate the availability of a hydrocarbyl migration pathway at formally iron(iv) centers to form new N–C bonds directly to N_2_, though product selectivity is highly dependent on the identity of the migrating group.

## Abstract 2

Silylation of iron-alkyl-dinitrogen complexes gives high-spin iron(iv) products that also have an iron(ii) resonance structure. The stability and reactivity of these compounds depends on the alkyl group, with insertion and homolysis observed.

## Graphical abstract

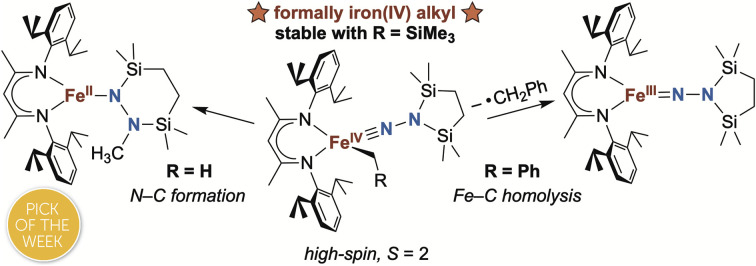

## Introduction

**Fig. 1:**
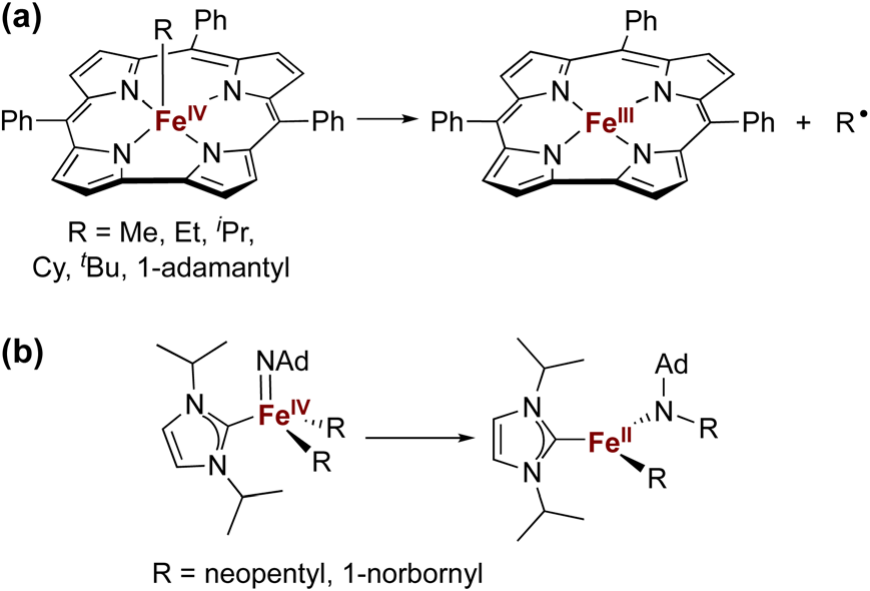
Examples of previously reported iron(iv) alkyl complexes and degradation pathways.

The isolation of iron(iv) compounds has been dominated by oxo, nitrido, and imido complexes, because the π-bonds in these compounds help to stabilize this high oxidation state.^1–13^ In contrast, reports of organometallic iron(iv) alkyl complexes are rare.^14–19^ One reason for this trend is that high-valent iron alkyl species are prone to Fe–C bond homolysis to produce alkyl radicals,^15,17^ exemplified by the well-documented reactivity of alkyliron porphyrin and corrole complexes (Fig. 1a).^20–26^ In contrast, Wolczanski and coworkers reported NHC-supported alkyliron(iv) complexes that were more resistant to Fe–C homolysis, and instead underwent alkyl group migration to the imido ligand to produce the corresponding amidoiron(ii) complexes (Fig. 1b).^16^ To our knowledge, this is the only well-characterized example of alkyl migration from a transition metal to a M=NR group, though a migration step may be involved in some reactions where a metal-alkyl undergoes amination by addition of an azide.^27,28^ Meyer has also reported insertion of a coordinated NHC ligand into the Co–N bond of a Co=NR complex,^29^ and there are several reports of carbenes inserting into Fe–N single bonds associated with supporting ligands.^29–33^

**Scheme 1:**
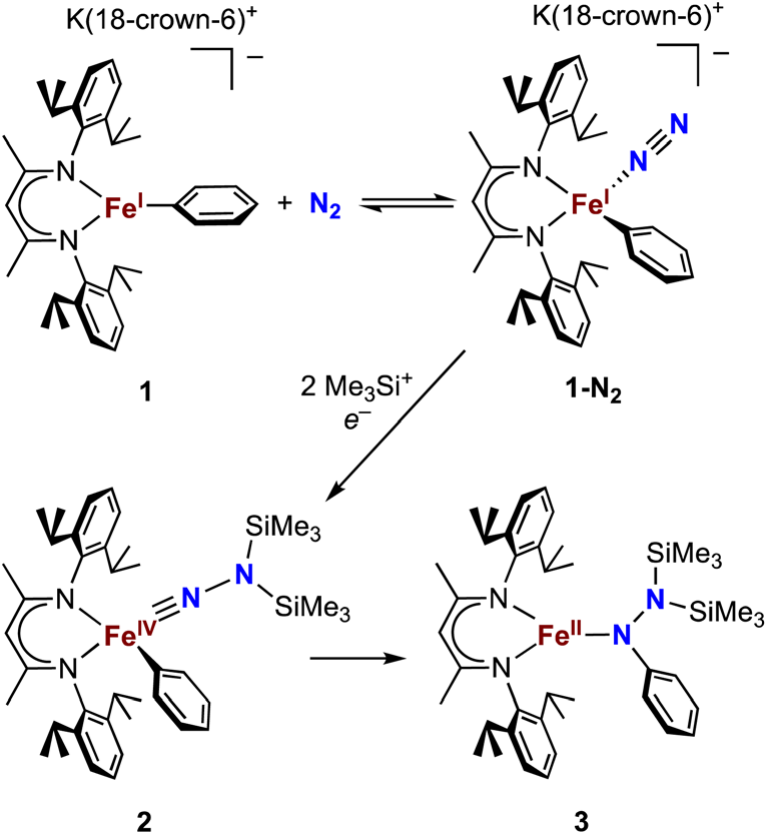
Formation of a formally iron(iv) hydrazido(2−) complex from N_2_, and subsequent migratory insertion of the aryl ligand.

Our research in this area emerged from the study of formally iron(iv) aryl species that undergo migration of the aryl group to the Fe-bound NNR_2_ group (Scheme 1).^34–36^ The reaction sequence of interest starts with the reaction of iron(i) aryl N_2_ complexes (1-N_2_) with two equivalents of Me_3_SiX (X = Br, I, OTf; OTf = trifluoromethanesulfonate) and one equivalent of reducing agent. This gives a double silylation at the distal N atom and net three-electron oxidation at the metal, resulting in a formally iron(iv) complex with aryl and hydrazido(2−) ligands (2). It is this complex that can perform the migration of the aryl group from Fe to the proximal N atom to form a new N–C bond in a hydrazido product (3).

So far, the migrations of hydrocarbyl groups from Fe to a multiply-bound N ligand that we have reported have been limited to iron–aryl complexes.^35,36^ Since N_2_ binding has been reported in a β-diketiminatoiron(i) alkyl complex as well,^37^ we hypothesized that iron(iv) alkyl hydrazido(2−) complexes might undergo alkyl migration by analogy to the aryl migrations. Previously, Peters described hydride migration to a hydrazido(2−) ligand at a formally iron(iv) center, suggesting that migration chemistry is not limited to aryl groups.^38^ However, we found that alkynyl groups do not migrate in the diketiminate system.^36^ Here, we describe a series of iron(i) alkyl complexes that bind N_2_ at low temperatures and their reactivities upon N_2_ silylation, including the characterization of the first formally iron(iv) alkyl hydrazido(2−) complex that is stable at room temperature. In some cases, N–C bond formation occurs but in other cases homolysis causes loss of the alkyl group without N–C bond formation, and the differences give insight into the feasibility of iron(iv) in these different environments. Density functional theory (DFT) calculations elucidate the competition between Fe–C homolysis and alkyl migration pathways. A preliminary description of this research has been shared in a preprint.^39^

## Results and discussion

### Binding of N_2_ to iron(i) alkyl complexes

**Fig. 2:**
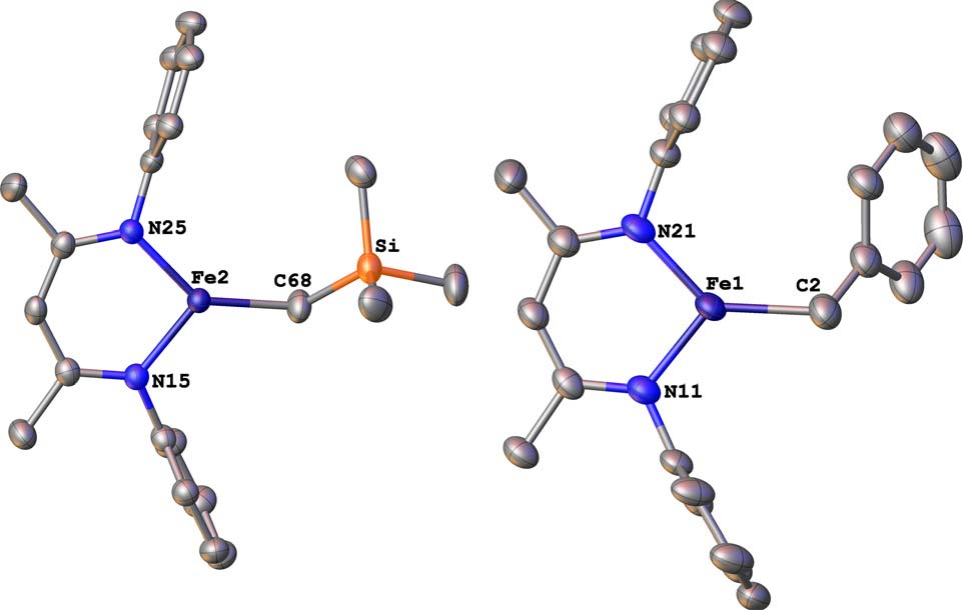
ORTEP diagrams of the iron(i) complexes 5a (left) and 5b (right) with thermal ellipsoids shown at 50% probability. H atoms, ^i^Pr groups, and K(18-crown-6)(THF)_2_^+^ cations are omitted for clarity, as well as a molecule of THF in the asymmetric unit of 5a.

**Scheme 2:**
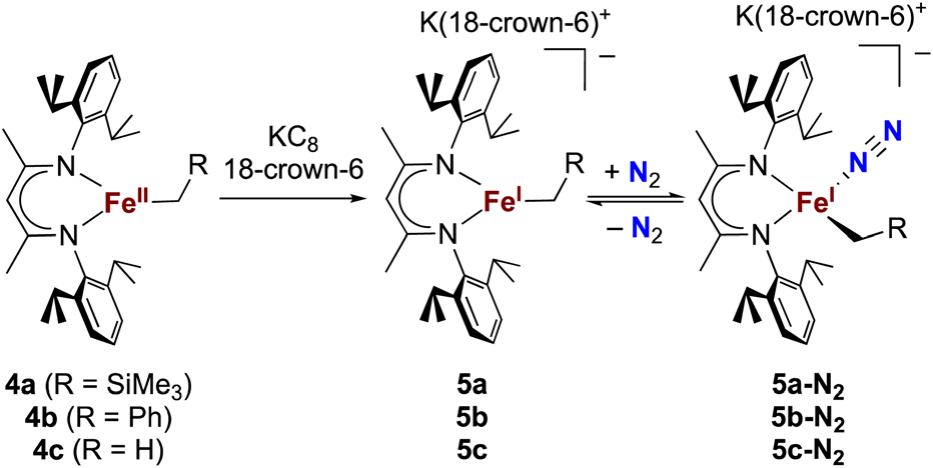
Synthesis of iron(i) alkyl complexes 5a–5c and reversible N_2_ binding.

The alkyl chemistry described here starts from the known high-spin three-coordinate iron(ii) alkyl complexes 4a–4c.^37,40–42^ Previous work has shown that an analogous β-diketiminatoiron(ii) alkyl complex can be reduced to an iron(i) complex, which upon cooling can bind N_2_ at the iron center.^34,37^ Accordingly, we prepared the iron(i) complexes 5a–5c by reduction of the corresponding iron(ii) alkyl complexes with KC_8_ in the presence of 18-crown-6, and isolated them in *ca.* 80% yield (Scheme 2). Complex 5c was isolated and fully characterized previously.^37^ Crystals of the new complexes 5a and 5b, grown from THF/hexanes, yielded X-ray crystallographic structures (Fig. 2). The average Fe–N bond lengths of 5a (1.928(5) Å) and 5b (1.918(3) Å) are equivalent to the distance in 5c (1.922(4) Å). The Fe–C bond length of 5b (2.063(4) Å) is longer than that in the starting iron(ii) complex 4b (2.041(2) Å),^42^ consistent with the lower oxidation state, while the Fe–C bond lengths of 5a and 4a are indistinguishable (5a, avg. 2.015(2) Å; 4a, 2.022(2) Å).^42^ The Mössbauer parameters of 5a (*δ* = 0.44 mm s^−1^, |Δ*E*_Q_| = 1.90 mm s^−1^) and 5b (*δ* = 0.27 mm s^−1^, |Δ*E*_Q_| = 1.75 mm s^−1^) are similar to those reported for high-spin 5c (*δ* = 0.38 mm s^−1^, |Δ*E*_Q_| = 2.06 mm s^−1^).^43^ Consistent with this assignment, solution magnetic susceptibilities indicate high-spin (*S* = 3/2) ground states for 5a (*μ*_eff_ = 4.3(1) *μ*_B_) and 5b (*μ*_eff_ = 4.3(1) *μ*_B_).

We then tested N_2_ binding at the iron(i) alkyl complexes at low temperature. Freezing solutions of 5a–5c in THF under an atmosphere of N_2_ led to a color change of the solutions from green to magenta, and thawing the solutions gave back the original green color. These color changes were not observed when freezing solutions under an atmosphere of Ar. van't Hoff analysis of the variable-temperature ^1^H NMR (5a and 5b) and UV-vis (5c) spectra gave the thermodynamic parameters shown in Table 1. The negative enthalpy and entropy values for each complex are consistent with N_2_ binding at lower temperatures, likely in an end-on fashion as proposed in the analogous iron β-diketiminate systems mentioned above.^34–37^

**Table 1:** Results from van't Hoff analysis of variable-temperature spectroscopic measurements of 5a–5c under N_2_

	Alkyl group	Δ*H* (kcal mol^−1^)	Δ*S* (e.u.)
5a	CH_2_SiMe_3_	−8.8(6)	−52(3)
5b	CH_2_Ph	−9.5(6)	−57(2)
5c	CH_3_	−4.3(7)	−33(3)

### Silylation of N_2_ in the trimethylsilylmethyliron complex leads to an isolable iron(iv) alkyl complex

**Scheme 3:**
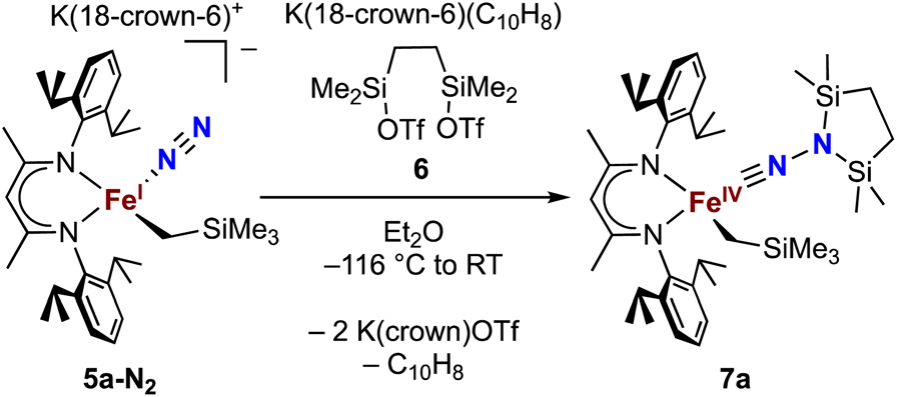
Synthesis of the formally iron(iv) complex 7a.

**Fig. 3:**
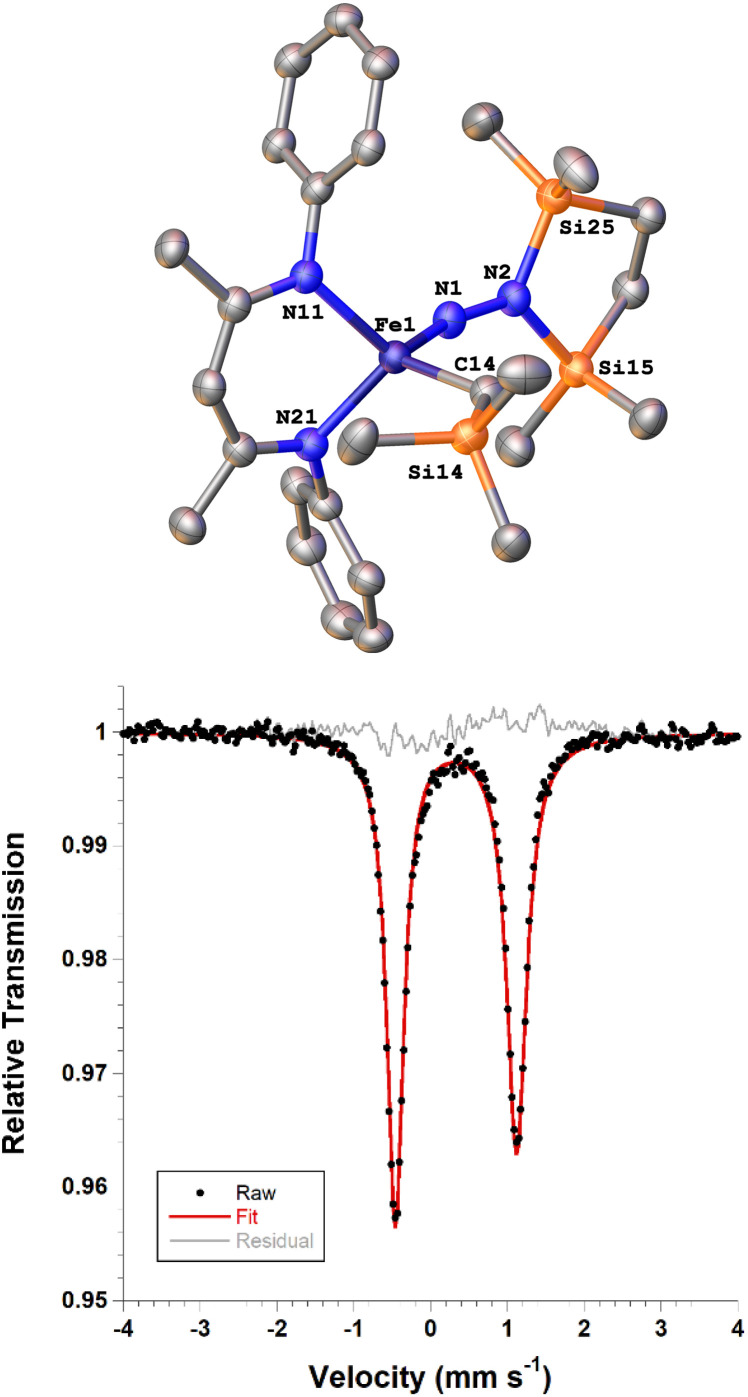
(Top) ORTEP diagram of 7a with thermal ellipsoids shown at 50% probability, with H atoms and ^i^Pr groups omitted for clarity. (Bottom) Solid state zero-field Mössbauer spectrum of 7a at 80 K. The black circles are the data, the red line is the fit, and the grey line is the residual (data – fit).

Next we explored the silylation reactions of the N_2_-bound iron(i) complexes to form formally iron(iv) complexes (Scheme 3). Addition of the bis(silyl) reagent 6 to a mixture of 5a-N_2_ and K(18-crown-6)(C_10_H_8_) (used as an external reductant) in Et_2_O at −116 °C led to an immediate color change from magenta to brown. The ^1^H NMR spectrum of the crude reaction mixture showed the formation of a new *C*_s_ symmetric complex in 73% spectroscopic yield. Cooling a concentrated hexamethyldisiloxane (HMDSO) solution at −35 °C overnight led to the isolation of brown crystals in 32% yield, which were identified by X-ray diffraction as the formally iron(iv) complex 7a (Fig. 3, top). The N–N bond length is 1.326(3) Å, which lies between the values for a N–N single bond (1.45 Å) and double bond (1.25 Å) in the corresponding organic N_2_H_*x*_ compounds, and is comparable to those in other four-coordinate iron hydrazido(2−) complexes as well as the phenyl complex 2 (1.340(4) Å).^35,44,45^ However, the bond lengths to iron in 7a are significantly different than those in 2. The Fe–N_hyd_ bond length (1.749(2) Å) and average Fe–N_nacnac_ bond length (2.051(1) Å) in 7a are ∼0.08 Å longer than those in 2 (1.673(3) and 1.970(2) Å, respectively). Additionally, the iron center in 7a adopts a distorted tetrahedral geometry (*τ*_4_ = 0.88) rather than the distorted trigonal pyramidal geometry in 2 (*τ*_4_ = 0.75).

The zero-field Mössbauer spectrum of 7a consists of a doublet with an isomer shift of *δ* = 0.33 mm s^−1^, which is much higher than the value of *δ* = 0.17 mm s^−1^ in 2 (Fig. 3, bottom). The higher isomer shift in 7a may indicate that the complex has a different ground spin state, and the longer bonds in 7a noted above suggest the higher spin state of *S* = 2. Furthermore, the bond distances to the iron center in 7a closely resemble those observed in a DFT model of 2 in an *S* = 2 state (see ESI†).^35^ Finally, a solution magnetic susceptibility measurement gave *μ*_eff_ = 5.0(2) *μ*_B_, which confirms the high-spin ground state.

### Electronic structure of the alkyl hydrazido(2−) complex

**Fig. 4:**
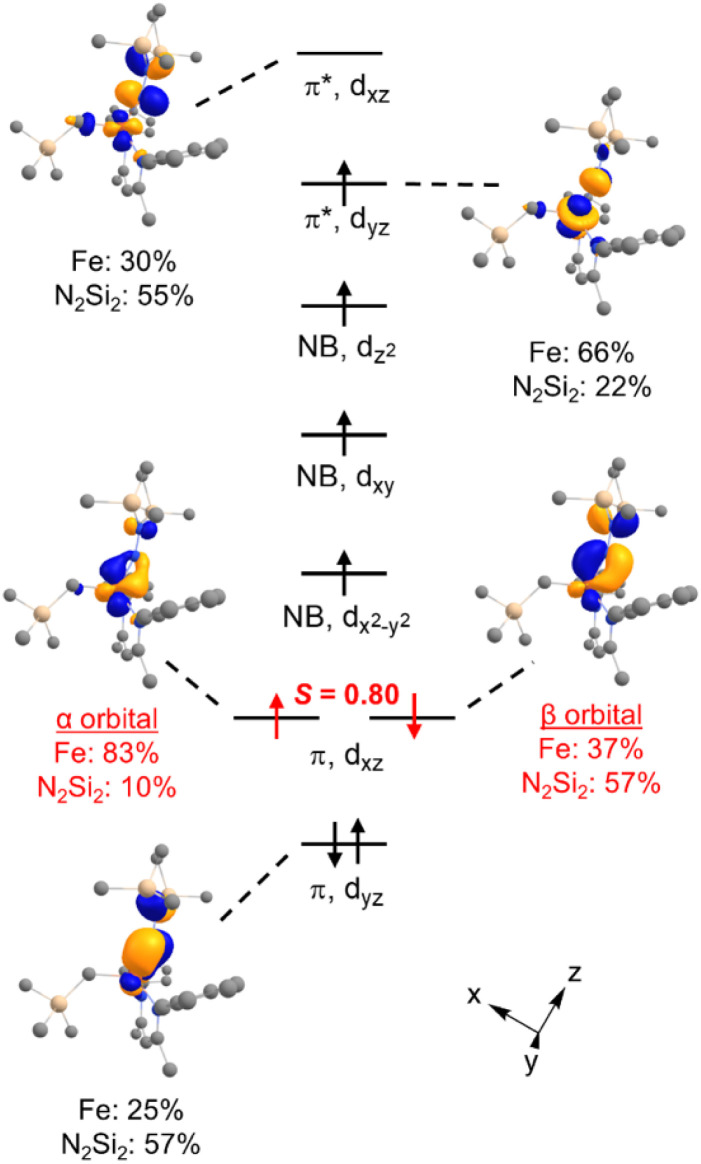
Qualitative molecular orbital diagram showing the QROs of 7a (*S* = 2) with selected QRO plots shown at an isovalue of 0.05 au. In red are the one-electron orbitals for the “corresponding pair” of electrons in the spin-polarized Fe–N π-bond.

DFT calculations were performed for greater insight into the electronic structure of 7a. The geometry of 7a in an *S* = 2 ground state was optimized at the B3LYP/def2-TZVP level, giving bond metrics that agree with those in the experimental X-ray structure of 7a (see ESI for details†). This computational model was further validated by computing^46^ the expected Mössbauer parameters of *δ* = 0.37 mm s^−1^ and |Δ*E*_Q_| = 1.63 mm s^−1^, which are close to the experimental values of 0.33 and 1.58 mm s^−1^, respectively. The quasi-restricted orbitals (QROs) from the model of 7a are shown in Fig. 4, with the *z* axis along the Fe–hydrazido(2−) bond. The formal bond order of the Fe–N_hyd_ bond is 2.5, as the Fe d_*yz*_ π* orbital is singly occupied. This is consistent with the longer Fe–N_hyd_ bond distance in 7a compared with that of 2, which has an *S* = 1 ground state and a formal Fe–N_hyd_ bond order of 3.

The Fe–N_hyd_ π bonding interaction involving the Fe d_*yz*_ orbital has more hydrazido than iron character, typical of a normal π bond. The π bonding interaction involving the Fe d_*xz*_ orbital, however, is more complex. The doubly-occupied Fe–N_hyd_ π bonding orbital has a relatively low orbital overlap of 〈*α*|*β*〉 = 0.80, and thus it is spin-polarized with the α electron lying 83% on the Fe and the β electron lying only 37% on Fe. Additionally, the unoccupied Fe–N_hyd_ π* orbital has significantly less Fe character than N_2_Si_2_ ligand character, indicative of an “inverted ligand field.”^47,48^ Taken together, the large spin polarization of one of the Fe–N_hyd_ π bonding orbitals and “inverted” ligand character suggests that the formally hydrazido(2−) ligand may be alternatively described as a neutral NNR_2_ (isodiazene) implying an iron(ii) oxidation state. This situation is analogous to that described for the metastable formally iron(iv) phenyl complex 2,^35^ with one spin-polarized orbital having Fe–N π character.

From the available data, it is difficult to discern why 7a has a different ground spin state than 2, as the energetic differences between the two are small. It is possible that the somewhat lower relative energy of the *S* = 2 state in 7a arises because the geometry is distorted from trigonal pyramidal toward tetrahedral, leading to a weaker ligand field.

To further probe the redox noninnocence of the NNR_2_ ligand in 7a, multireference CASSCF(8,7) calculations were performed on the DFT-optimized *S* = 2 structure; details are shown in the SI. The dominant configuration (72%) has the expected Hund filling, corresponding to a hydrazido(2−) ligand. The next two most important configurations have single (16%) and double (10%) occupation of the Fe–N π antibonding orbital involving the Fe d_*xz*_ orbital, which correspond to iron(iii) hydrazido and iron(ii) isodiazene descriptions of 7a, respectively. Thus, both single reference and multireference calculations point to ligand noninnocence of the NNR_2_ ligand, to an extent that is comparable to the phenyl and alkynyl analogues.^35,36^

### The trimethylsilylmethyl group does not migrate

We then turned to the solution behavior of 7a. Complex 7a was much more stable in solution than 2, showing only about 25% decomposition after 4 days in C_6_D_6_ solution at room temperature (whereas 2 is completely consumed within a few hours at room temperature). Heating a C_6_D_6_ solution of 7a at 80 °C for 2 hours led to its complete consumption. However, the product was not alkyl migration (in analogy to 2) but rather formation of the iron(ii) alkyl complex 4a (24%), the hydrazido(2−) product 8 (29%), and the amido complex LFeN(Me_2_Si(CH_2_)_2_SiMe_2_) (36%) (see ESI for details†). By analogy to a related N–Si bond cleavage in an iron silyldiazenido complex by Ashley,^49^ we speculate that the mechanism for the formation of 4a might involve homolytic cleavage of N–Si bonds, though there is also cleavage of Fe–C, Fe–N, and N–N bonds to yield the other observed products.

Why does the trimethylsilylmethyl group in 7a not migrate as previously observed for the phenyl group in 2? Though it is tempting to attribute this to the difference in the spin state, we were not able to optimize transition states for migration of the CH_2_SiMe_3_ group (triplet or quintet states) to assess the impact of spin state and TS geometry with DFT. However, since the electronically similar methyl group does migrate (see below), there is some evidence that steric effects play a role.

### Attempts to generate the iron(iv)-benzyl lead to homolysis

**Scheme 4:**
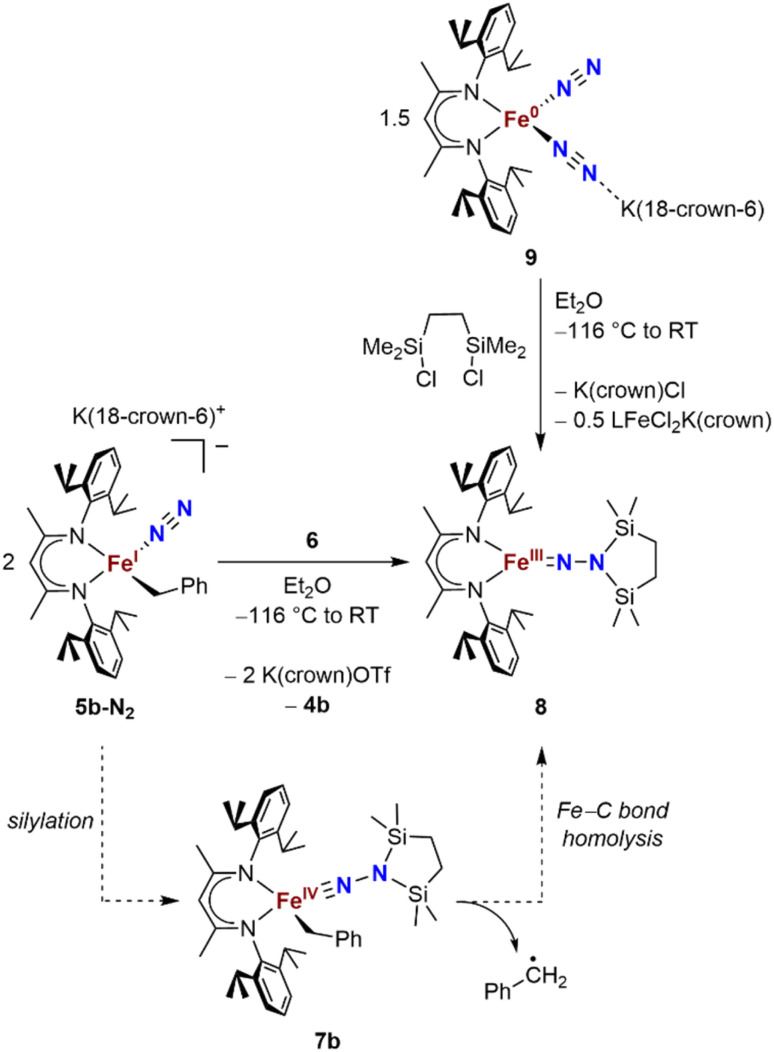
Syntheses of the iron(iii) hydrazido(2−) complex 8, and proposed mechanism of formation from 5b-N_2_.

**Fig. 5:**
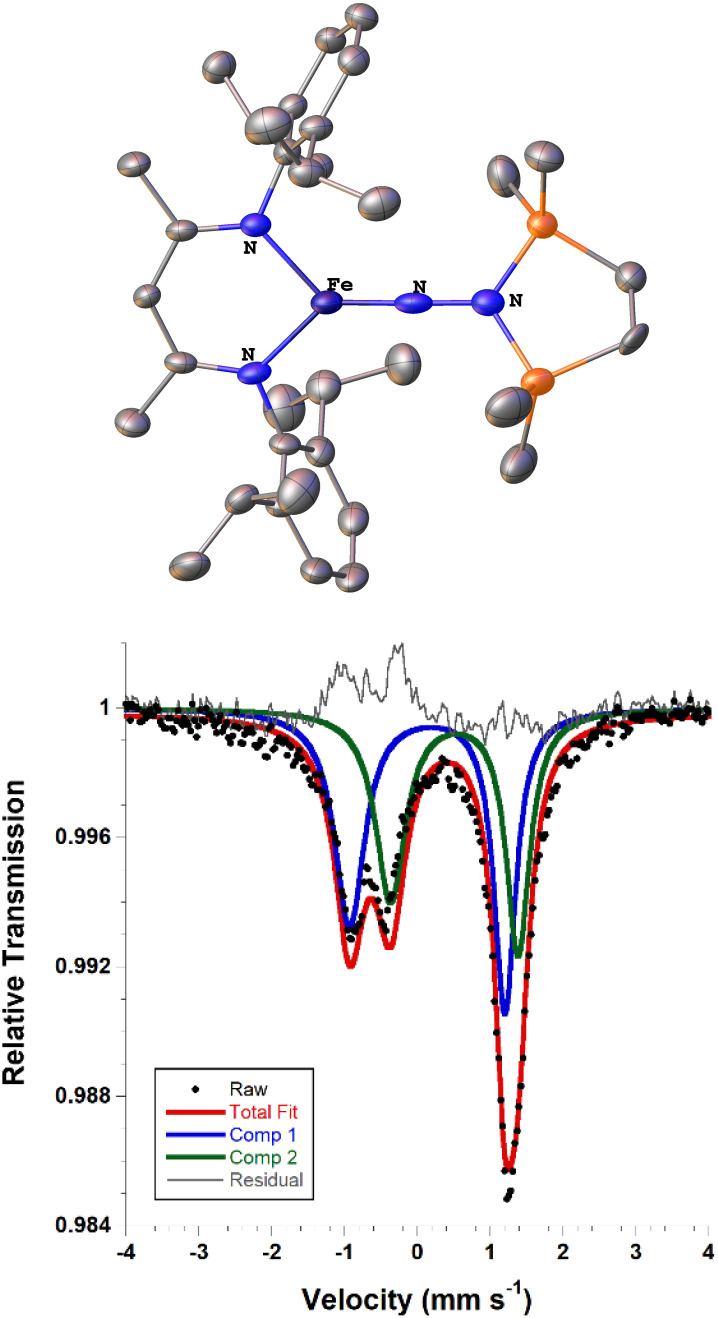
(Top) ORTEP diagram of 8 with thermal ellipsoids shown at 50% probability and H atoms omitted for clarity; due to apparent disorder in the core, the structure is for connectivity only. (Bottom) Solid state zero-field Mössbauer spectrum of 8 at 80 K. The black circles are the data, the blue and green lines are the components of the fit, the red line is the sum of the components, and the grey line is the residual (data – fit).

We also explored the N_2_ silylation reactivity of the other iron(i) alkyl complexes. In contrast with the silylation of 5a-N_2_, the use of K(18-crown-6)(C_10_H_8_) as an external reductant in the reaction of the iron(i) benzyl complex 5b-N_2_ and the silyl triflate 6 led to an intractable mixture of unidentified species. However, it has previously been shown in the synthesis of a related aryl diazenido species that the starting iron(i) aryl complex can provide the necessary electron equivalent in the reaction (unfortunately limiting the yield of silylated product to a maximum of 50% based on iron, since part of it is a sacrificial reductant).^34^ Thus, addition of 6 to a solution of 5b-N_2_ in Et_2_O at −116 °C without an external reductant led to the formation of the oxidized iron(ii) product 4b in 64% spectroscopic yield (yield based on the stoichiometry in Scheme 4, quantified by ^1^H NMR spectroscopy), as well as a new *C*_2v_ symmetric species 8 in 22% spectroscopic yield (Scheme 4, middle). This same species 8 was also identified in the decomposition mixture of 7a in 27% spectroscopic yield, indicating that it had lost the alkyl group. Indeed, X-ray diffraction revealed 8 to be an iron(iii) hydrazido(2−) complex (Fig. 5, top).

Compound 8 is closely related to an iron(iii) hydrazido(2−) complex we previously reported, the trimethylsilyl analogue LFeNN(SiMe_3_)_2_ (L = 2,4-bis(2,6-diisopropylphenylimido)pentyl).^50^ Accordingly, complex 8 could be prepared independently from the reaction of the iron(0)-bis(dinitrogen) complex 9 and 1,2-bis(chlorodimethylsilyl)ethane in 22% isolated yield (Scheme 4, upper right). The products from the two synthetic methods had identical ^1^H NMR spectra, further supporting the proposed composition of 8.

We propose that the route from 5b-N_2_ to 8 begins with silylation to give the formally iron(iv) complex 7b followed by Fe–C bond homolysis,^17,26,51,52^ producing a benzyl radical (Scheme 4, bottom). In order to test this idea, we performed trapping experiments using the radical scavenger TEMPO (TEMPO = 2,2,6,6-tetramethylpiperidine 1-oxyl). When TEMPO was added to the reaction mixture immediately after silane addition at −116 °C, ^1^H NMR spectroscopy showed >80% formation of TEMPO-Bn.^53^ This implies the formation of a transient intermediate that can release benzyl radicals.

Unfortunately, the X-ray crystal structure solution of 8 had a second component in the core, and the disorder prevents us from deriving reliable metrical parameters, and we were unable to obtain satisfactory results from CHN analysis. Despite these problems that prevent deep study of 8, we note in passing several intriguing aspects of its spectroscopic properties. Similar to the previously characterized trimethylsilyl analogue LFeNN(SiMe_3_)_2_,^50^ the solid state Mössbauer spectrum of crystalline 8 collected at 80 K shows two doublets in a 1 : 1 ratio with isomer shifts of 0.13 and 0.51 mm s^−1^ (Fig. 5, bottom). In the previous work, the 1 : 1 ratio was explained by the presence of two molecules with significantly different bond distances, which coexist in alternating positions in the crystals. Comparison to calculations and fitting of magnetic susceptibility data supported our conclusion that the two molecules had different spin states (*S* = 1/2 and *S* = 3/2).^50^ In order to determine whether this might be the case in 8 as well, DFT calculations were carried out to determine the relative energies of the *S* = 1/2 and *S* = 3/2 states using geometry-optimized structures of 8 (Table 2). Calculations using both the BP86 and B3LYP functionals show a small energy difference between the doublet and quartet states: using BP86 the low-spin conformer is lower in energy by 9 kcal mol^−1^, while B3LYP predicts the high-spin state to be lower in energy by 2 kcal mol^−1^. This difference in lowest energy calculated spin conformer is not surprising, as hybrid functionals such as B3LYP have been shown to favor higher spin states.^54^ These small calculated differences in energy suggest that the actual spin isomers could indeed be isoenergetic. Importantly, the calculated Mössbauer parameters of the geometry-optimized doublet and quartet DFT models are in excellent agreement with the two signals in the spectrum of 8 (Table 2).^46^

**Table 2:** Comparison of Mössbauer parameters between experiment and DFT models (BP86/def2-TZVP or B3LYP/def2-TZVP)

Compound	Functional	Spin state	Rel. energy (kcal mol^−1^)	*δ* (mm s^−1^)	|Δ*E*_Q_| (mm s^−1^)
8	Exp.	1/2		0.13	2.13
		3/2		0.51	1.75
	BP86	1/2	0	0.12	2.04
		3/2	9	0.48	1.83
	B3LYP	1/2	2	0.24	1.76
		3/2	0	0.67	1.36
LFeNN(SiMe_3_)_2_ (ref. 55)	Exp.	1/2		0.22	1.99
		3/2		0.46	1.16
	BP86	1/2	0	0.14	1.93
		3/2	9	0.51	1.72
		5/2	31	0.61	3.69
	B3LYP	1/2	1		
		3/2	0		
		5/2	15		

**Fig. 6:**
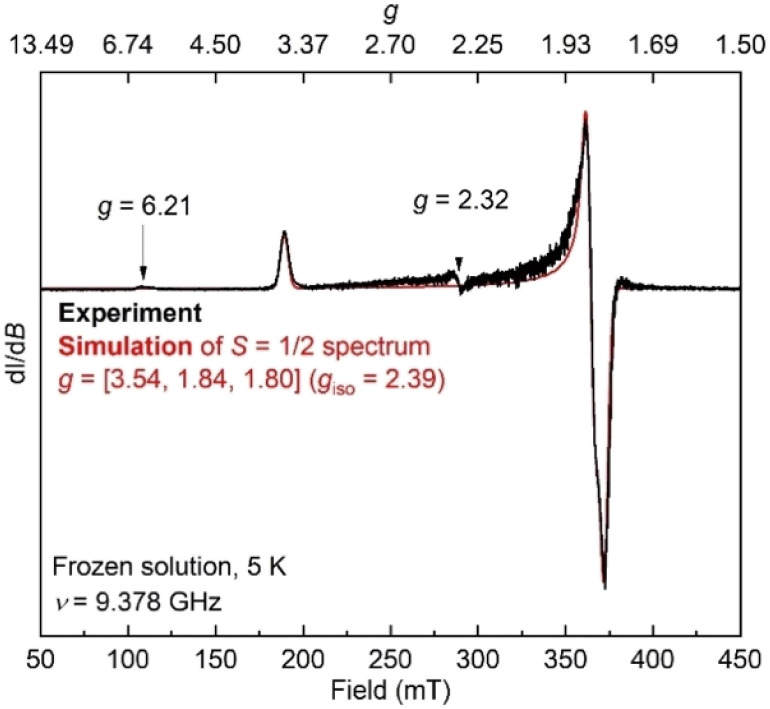
EPR spectrum of 8 in toluene at 5 K (black) and simulation of the *S* = 1/2 component (red).

Additionally, the electron paramagnetic resonance (EPR) spectrum of a frozen toluene solution of 8 collected at 5 K shows two overlapping signals consistent with coexisting *S* = 1/2 and *S* = 3/2 species (Fig. 6). Simulating the spectrum gives an *S* = 1/2 species with *g* = [1.80, 1.84, 3.54]. The large anisotropy of *g* values was similarly observed in the previously reported trimethylsilyl analogue which had *g* = [1.79, 1.84, 3.61].^50^ The small signals with *g*_eff_ values of 6.21 and 2.32 are reminiscent of a three-coordinate, intermediate-spin (*S* = 3/2) iron(iii) imido complex with the same diketiminate supporting ligand,^55,56^ supporting this assignment. Overall, it appears that spin isomerism is present in this three-coordinate iron(iii) hydrazido(2−) complex. Future studies will aim to unravel the reason why the 1 : 1 ratio is observed even without apparent crystal constraints.

### Methyl migration followed by silyl migration leads to a different kind of complex, with spin and coordination isomers

**Scheme 5:**
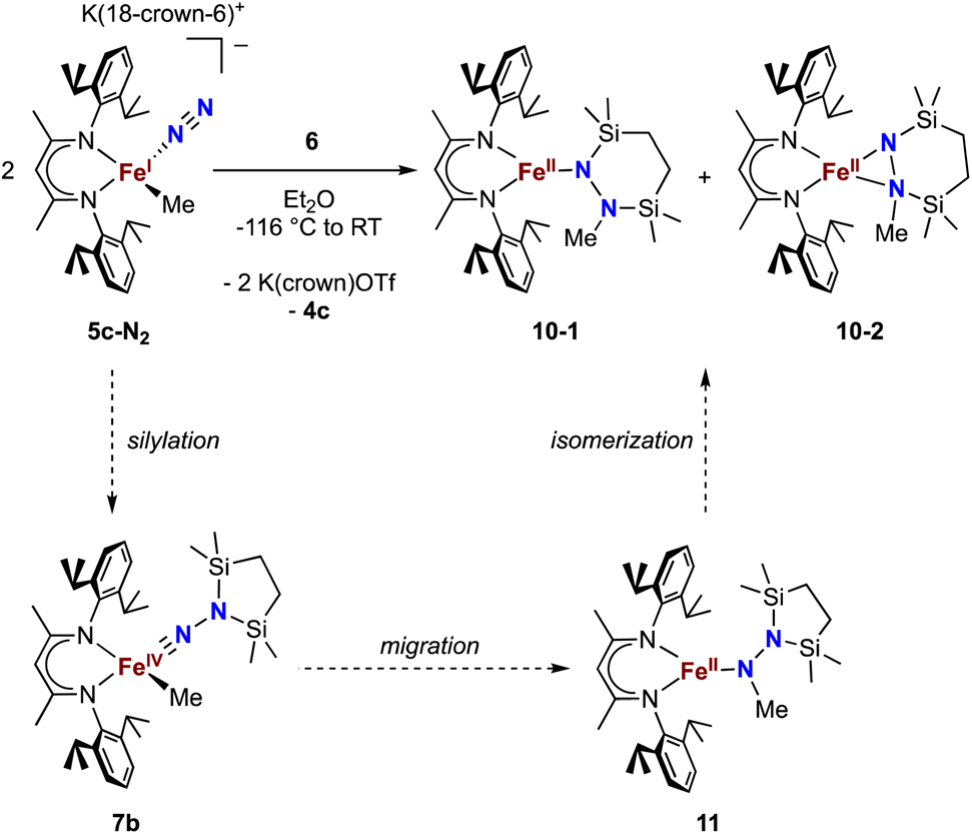
Synthesis of the methylhydrazido complex 10, which crystallizes as two isomers 10-1 and 10-2. Along the bottom is shown the proposed mechanism of the reaction.

**Fig. 7:**
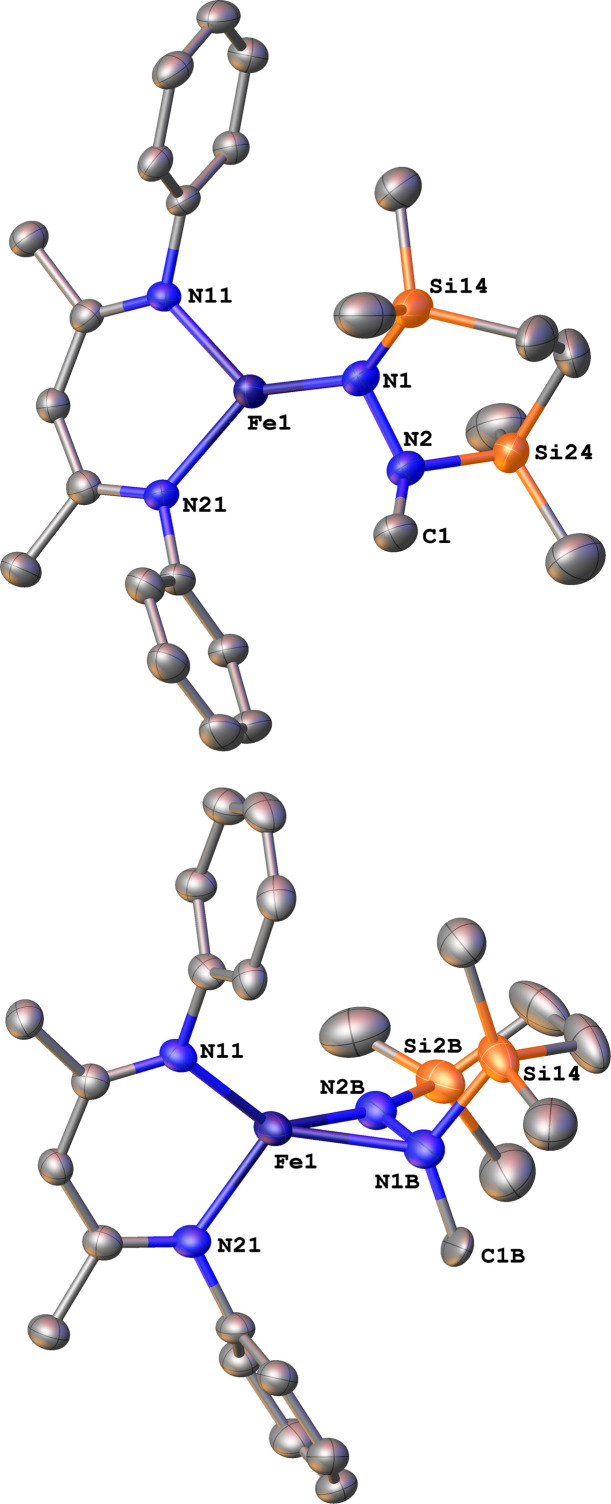
ORTEP diagrams of 10-1 and 10-2 with thermal ellipsoids shown at 50% probability and H atoms and ^i^Pr groups omitted for clarity. The η^1^ isomer (major component 10-1, 75%) is shown on the top, and the η^2^ isomer (minor component 10-2, 25%) is shown on the bottom.

Finally, we explored the silylation of the iron(i) methyl complex 5c (Scheme 5). Addition of the disilyl electrophile 6 to a solution of 5c-N_2_ in Et_2_O at −116 °C led to an immediate color change from magenta to brown, and then the mixture turned yellow upon warming to ambient temperature. The ^1^H NMR spectrum of the crude reaction mixture showed the presence of two species: the oxidized iron(ii) complex 4c (57%) and a new *C*_s_ symmetric species 10 (37%). In this case, no formation of 8 (which would result from loss of a methyl radical) was observed in the crude mixture. X-ray crystallography identified 10 as a 1,2-bis(silyl)methylhydrazido complex (Fig. 7), with disorder that indicates co-crystallization of η^1^ and η^2^ isomers (described in more detail below). A reasonable mechanism for the formation of 10 is through the initial formation of the formally iron(iv) complex 7c, followed by methyl migration to form the expected methylhydrazido complex 11 (Scheme 5, bottom). This complex could then isomerize through a silyl shift to give the observed product 10. Shifting of the bis(silyl)methylhydrazido ligand in this way has precedent in the isomerization of free bis(trimethylsilyl)methylhydrazine, which can shift its silyl groups in the presence of catalytic amounts of base.^57^ Most relevantly, Peters has reported an example of a disilylhydrazido(2−) complex in which the silyl group is proposed to go through an intermediate that resembles 10.^58^

**Scheme 6:**
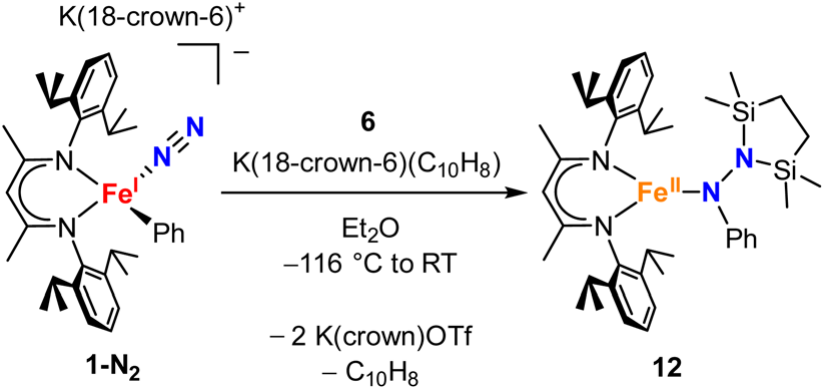
Synthesis of the phenyl-migrated complex 12.

**Fig. 8:**
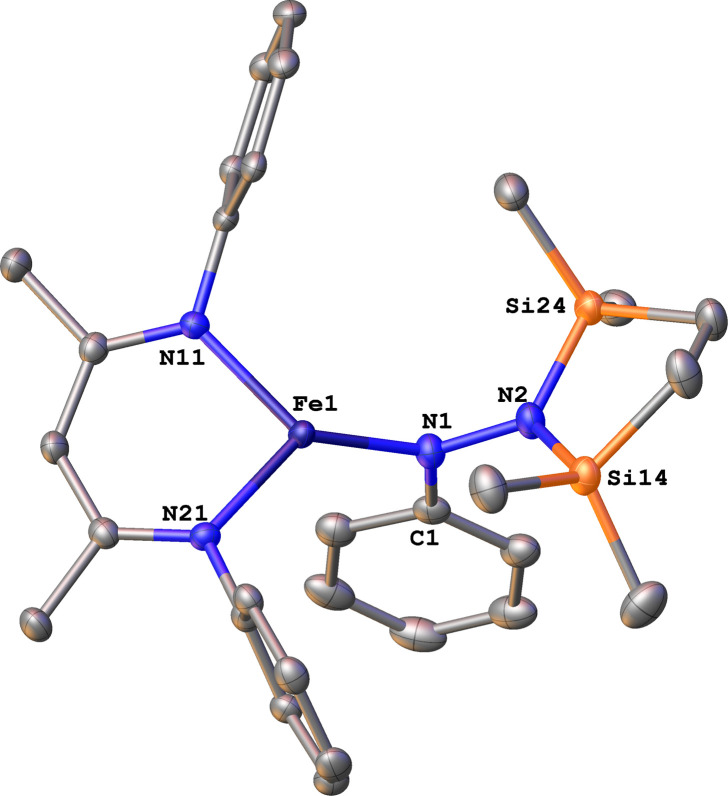
ORTEP diagram of 12 with thermal ellipsoids shown at 50% probability. H atoms and ^i^Pr groups omitted for clarity.

To test whether the ability of the silyl to shift arises somehow from the change from trimethylsilyl to the bis(silyl) reagent 6, we prepared the bis(silyl) analogue of 2. Specifically, the iron(i) phenyl complex 1-N_2_ was treated with 6 in the presence of K(18-crown-6)(C_10_H_8_) (Scheme 6),^34^ which led to the expected 1,1-bis(silyl)phenylhydrazido complex 12 (Fig. 8) without the silyl shift observed in the methyl system. It is unclear whether the lack of isomerization by 12 to give the 1,2-bis(silyl)phenylhydrazido complex is the result of a high kinetic barrier or a thermodynamically unfavorable reaction. Regardless, this result suggests that the use of the bis(silyl) reagent is not the sole reason for hydrazido isomerization, and that the identity of the hydrocarbyl group on the hydrazido ligand influences its ability to take part in the silyl shift.

### Isomers of compound 10

The X-ray crystal structure of 10 was disordered, and the best-fit model has two components, as mentioned above. Though both components contain the same cyclic ligand, its coordination to iron in one of the components (10-1) is η^1^ (roughly 75% occupancy), whereas the other (10-2) is η^2^ through both nitrogen atoms (roughly 25% occupancy). Accordingly, the Mössbauer spectrum of 10 has a shoulder that is indicative of multiple components, and the spectrum could be fit with two or three Mössbauer doublets (Fig. S21 and S22†). Because both X-ray and Mössbauer methods suggested multiple isomers in samples that were pure (as judged by CHN analysis), we explored the energies and geometries of both isomers in triplet and quintet states using DFT geometry optimizations (Table S10†). These indicated that there are three forms that have low energies (within 4 kcal mol^−1^ of one another): an η^1^ isomer in a quintet state, and η^2^ isomers in triplet or quintet states. Further, a superposition of signals with the calculated isomer shift and quadrupole splitting values from these three models fits well to the experimental Mössbauer spectrum (Table S11†). Finally, the predominance of quintet states agrees with the experimental solution magnetic moment of 10 of *μ*_eff_ = 5.1(2) *μ*_B_. Though the limited amount of experimental information hinders our ability to delve further, this combination of spectroscopy, crystallography, and computations is consistent with the idea that multiple isomers could coexist and be distinct in the solid-state structure (though equilibrating on a subsecond timescale such that one set of resonances is observed by ^1^H NMR spectroscopy).

### DFT computations give insight into product selectivity

**Fig. 9:**
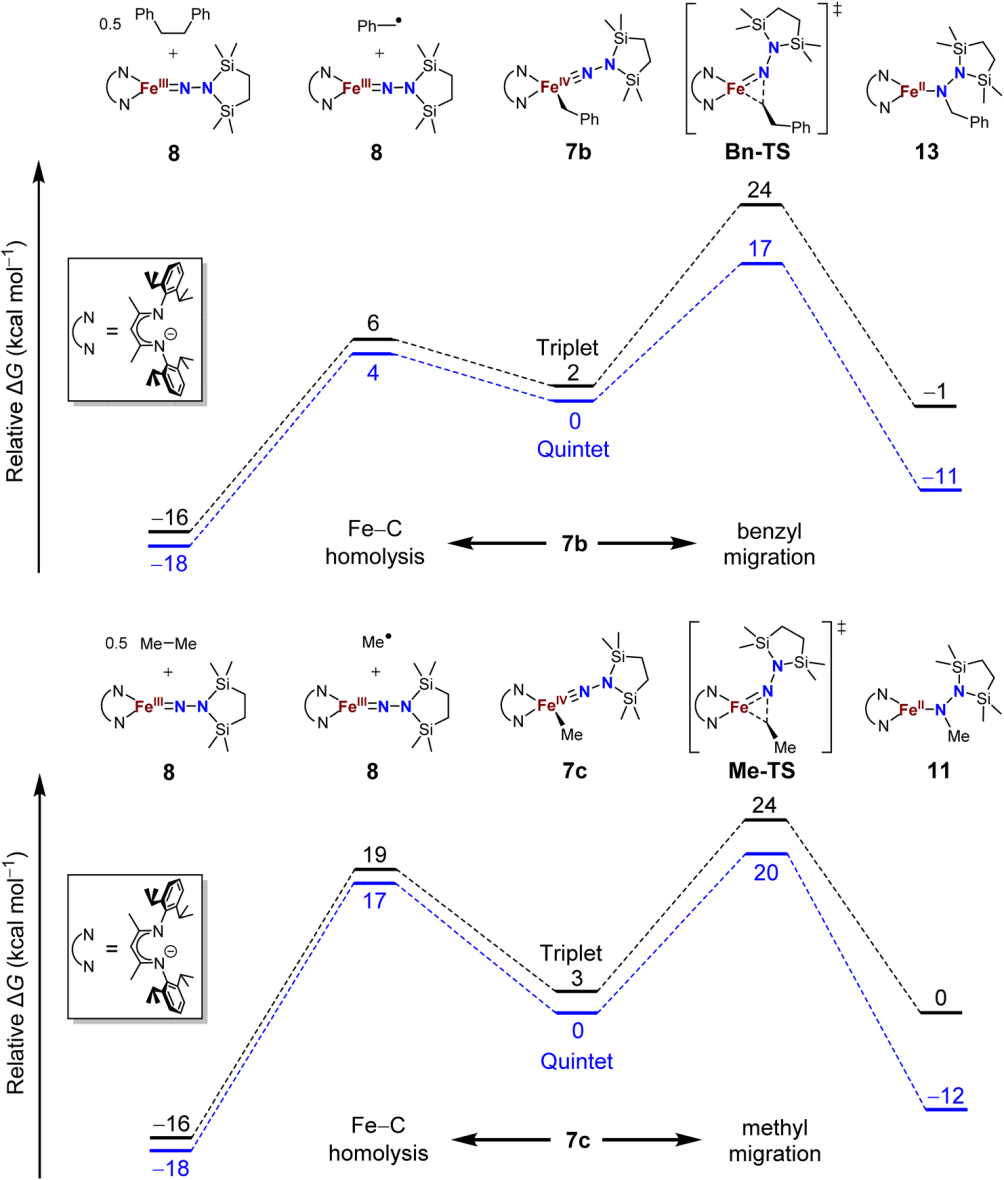
Potential energy surfaces of Fe–C bond homolysis (to the left) *versus* alkyl migration (to the right) starting from the proposed iron(iv) benzyl (top) and methyl (bottom) complexes from DFT calculations (B3LYP/def2-TZVP).

The difference in product speciation between the silylation reactions of the benzyl complex 5b and the methyl complex 5c suggests that there may be two competing reaction pathways upon formation of the resulting formally iron(iv) alkyl complexes: Fe–C homolysis and hydrocarbyl migration. These two competing reaction pathways were investigated using DFT calculations (Fig. 9, next page). Geometries were optimized using DFT (B3LYP/def2-TZVP) in both the triplet and quintet states to probe possible spin crossover as observed in the aryl migration mechanism.^35^

No transition states were found for the Fe–C_alkyl_ bond homolysis steps in either the benzyl (Fig. 9, top) or methyl (Fig. 9, bottom) system. This is not surprising, because bond homolysis reactions often have barriers very close to the BDFE if uncomplicated by steric constraints or spin state changes.^59^ The quintet surface was calculated to be the lowest energy pathway for Fe–C homolysis in both systems. The products of Fe–benzyl bond homolysis are only 4 kcal mol^−1^ uphill from 7b, while products of Fe–methyl homolysis are higher at 17 kcal mol^−1^ relative to 7c. This difference likely stems from the greater stability of a benzyl radical relative to a methyl radical.^60^ Therefore, alkyl radical formation is expected to be much more rapid for the benzyl complex.

To assess the alkyl migrations, the nudged elastic band method was used to find transition states, which were then optimized. The lowest energy pathways for both benzyl and methyl migration were found on the quintet surfaces. The calculated barriers for benzyl (17 kcal mol^−1^) and methyl (20 kcal mol^−1^) migration are similar to the experimentally measured activation barriers for aryl migration reactions (21–23 kcal mol^−1^).^35^

The significantly lower energy for Fe–C_Bn_ bond homolysis compared to the barrier for benzyl migration is consistent with the observation that silylation of 5b-N_2_ gave homolysis to the iron(iii) complex 8 rather than migration. Meanwhile, the energy for Fe–C_Me_ bond homolysis starting from 7c is similar to the barrier for methyl migration, suggesting that after the silylation of 5c-N_2_, homolysis could potentially compete with the migration pathway. We cannot rule out the possibility that radical recombination after Fe–C_Me_ bond homolysis forms 11 in a two-step migration mechanism.^21^

## Conclusions

In summary, the above studies have demonstrated that silylation of iron(i) alkyl N_2_ complexes can give formally iron(iv) alkyl hydrazido(2−) species, with interesting differences in the subsequent reactivity. A methyl group does migrate, though it is followed by a silyl migration. With the bulkier trimethylsilylmethyl, the alkyl hydrazido(2−) product can be isolated, indicating that steric effects slow the migration. The resistence to migration in the iron(iv) compound in this case allowed us to characterize it in detail, and show that it has a high-spin ground state. The benzyl compound contrasts with the others, with a weak Fe–C bond leading to homolysis. DFT calculations support the feasibility of each proposed pathway and show that they are indeed expected to be kinetically competitive.

An important conclusion is that the use of formally iron(iv) centers enables migrations that can form N–C_alkyl_ as well as N–C_aryl_ bonds to a hydrazido(2−) ligand derived from N_2_, showing the generalizability of this novel approach for forming N–C bonds that come from organometallic fragments and N_2_. However, attempted migration of alkyl ligands is problematic when there is the potential to form a stabilized alkyl radical, because homolysis may result in rapid Fe–C bond cleavage. Thus, while alkyl migration was feasible for one case of N–C bond formation from N_2_, the selectivity of the reaction was poorer for alkyl than the previously observed aryl migration.

## Data availability

The data are in the ESI.†

## Author contributions

S. F. M. and P. L. H. conceptualized the project, S. M. B. and R. X. H. and B. Q. M. collected and analyzed the data, S. M. B. wrote the original draft, and P. L. H. supervised the research and edited the manuscript.

## Conflicts of interest

There are no conflicts to declare.

## Supplementary Material

SC-015-D3SC05939A-s001

SC-015-D3SC05939A-s002
