# Supplementary material for: Iron(iv) alkyl complexes: electronic structure contributions to Fe–C bond homolysis and migration reactions that form N–C bonds from N2
Source: Chem Sci. 2024 Jan 24;15(10):3485–94. doi: 10.1039/d3sc05939a (PMC10915813; doi:10.1039/d3sc05939a)
Supplement: SC-015-D3SC05939A-s001 [file SC-015-D3SC05939A-s001.pdf]

Electronic Supplementary Material (ESI) for Chemical Science.
This journal is © The Royal Society of Chemistry 2024

Supporting Information

Iron(IV) alkyl complexes: electronic structure contributions to Fe–C bond homolysis and migration reactions that form N–C bonds from N_2

*Samuel M. Bhutto, Reagan X. Hooper, Sean F. McWilliams, Brandon Q. Mercado, Patrick L. Holland**

Department of Chemistry, Yale University, New Haven, Connecticut 06520

* email: patrick.hollland@yale.edu

Materials and Methods

General considerations. All manipulations were performed under an N_2 (or Ar where specified) atmosphere by Schlenk techniques or in an M. Braun or Vigor glovebox maintained at or below 1 ppm of O_2 and H_2O. Glassware was oven-dried at 160 ˚C for at least 12 h before use. Graphite, Celite, and molecular sieves were dried at 200 ˚C under vacuum for at least 12 h. Pentane, hexanes, diethyl ether, THF, and toluene were purified by passage through activated alumina and Q5 columns from Glass Contour Co., under Ar. Hexamethyldisiloxane (HMDSO) was degassed by sparging with N_2 for 3 hours. All solvents were stored over 4 Å molecular sieves. Benzene-d_6 was dried over activated alumina and stored over 4 Å molecular sieves. THF-d_8 and toluene-d_8 were vacuum transferred from sodium benzophenone ketyl solutions and stored over 4 Å molecular sieves. Compounds $[LFeBr]_2$,[1] **1**,[2] **4a**,[3] **4b**,[3] **5c**,[4] **9**,[5] and K(18-crown-6)($C_{10}H_8$)[6] were prepared as previously reported. Potassium was obtained from Sigma-Aldrich (98%). Potassium on graphite (KC_8) was prepared by heating stoichiometric amounts of potassium and graphite at 140 °C under an argon atmosphere. 1,2-Bis(chlorodimethylsilyl)ethane (96%), silver trifluoromethanesulfonate (AgOTf) (≥99%), TEMPO (98%), and methylhydrazine (98%) were obtained from Sigma-Aldrich. Methylhydrazine was dried over barium oxide and vacuum distilled prior to use. 18-Crown-6 was obtained from ACROS Organics. 18-Crown-6 was dried by washing with acetonitrile prior to use. ^{1}H NMR data were recorded on a Bruker Avance 400 or 500 spectrometer (400 or 500 MHz) at room temperature unless otherwise noted. Chemical shifts in ^{1}H NMR spectra are referenced to external $SiMe_4$ using the residual protiated solvent peaks in C_6D_5H (δ 7.16 ppm) and THF-d_7 (δ 3.58 ppm). Resonances were singlets unless otherwise noted. Line fitting of some resonances in the ^{1}H NMR spectra was performed using MestReNova 10.0.1.

Elemental analyses were obtained from the CENTC Elemental Analysis Facility at the University of Rochester. Microanalysis samples were weighed on a PerkinElmer Model AD-6 Autobalance, analyzed on a PerkinElmer 2400 Series II Analyzer and handled in a VAC Atmospheres glovebox under argon. IR spectra were collected on a Bruker Alpha Platinum IR Spectrometer in ATR mode. UV-vis spectra were recorded on a Cary 50 spectrometer using Schlenk-adapted quartz cuvettes with a 1 mm path length. Continuous-wave X-Band EPR spectra were recorded in perpendicular mode using a Bruker EleXsys EPR Spectrometer equipped with an ER 049X microwave bridge. The spectra were simulated using EasySpin.[7] Solid and solution Mössbauer samples were packed in Delrin sample cups and loaded into the spectrometer at 77 K. Mössbauer measurements were performed using a SEE Co. MS4 Mössbauer spectrometer integrated with a Janis SVT-400T He/N_2 cryostat for measurements at 80 K (unless otherwise noted) with a 0.07 T applied magnetic field. Isomer shifts were determined relative to α-iron at 298 K. All Mössbauer spectra were fit using the program WMoss (SEE Co.) or MossA,[8] using Lorentzian doublets.

Preparation of [K(18-crown-6)(THF)$_2$][LFeCH$_2$SiMe$_3$] (5a). A mixture of **4a** (500 mg, 0.892 mmol) and 18-crown-6 (259 mg, 0.981 mmol) was dissolved in THF (15 mL), and the resulting orange solution was cooled to –35 °C. Solid KC_8 (133 mg, 0.981 mmol) was added to the stirring orange solution leading to a color change to green upon mixing. The reaction warmed to ambient temperature and was stirred for 90 minutes. The mixture was filtered through Celite yielding a green filtrate, and the filter was washed with THF (12 mL). The two filtrates were combined and concentrated under vacuum to 5 mL, layered with hexanes (5 mL), and placed in a –35 °C freezer overnight yielding green crystals of **5a** (787 mg, 82%). ^{1}H NMR (400 MHz, THF-d_8) δ 31 (4H), 20 (2H), 3.3 (24H), 2.5 (12H), 0.5 (6H), –4 (9H), –31 (2H), –145 (1H) ppm. Two resonances

corresponding to 4H and 12H were not found and may be broadened too much to be observed. μ_{eff} (Evans, THF-d_8, 298 K) = 4.3(1) μ_B. Zero-field Mössbauer (solid, 80 K) δ = 0.44 mm s^{-1}, $|\Delta E_Q|$ = 1.90 mm s^{-1}, Γ_L = 0.35 mm s^{-1}, Γ_R = 0.41 mm s^{-1}. IR (ATR, near, cm^{-1}): 3064 (w), 3025 (w), 2967 (m), 2893 (m), 2875 (m), 2840 (w), 2756 (w), 2721 (w), 2698 (w), 1621 (w), 1595 (w), 1545 (w), 1523 (m), 1467 (m), 1438 (s), 1410 (s), 1369 (m), 1356 (m), 1323 (s), 1288 (w), 1253 (m), 1233 (w), 1177 (m), 1110 (s), 1060 (m), 1046 (m), 1025 (m), 964 (s), 937 (m), 863 (m), 840 (m), 793 (m), 760 (m), 736 (m), 715 (w), 699 (w), 674 (w), 664 (w), 627 (w), 602 (w), 575 (w), 495 (m), 442 (m). UV-vis (THF, ε in mM^{-1}cm^{-1}): 758 (3.2), 454 (3.9), 422 (4.4), 374 (5.5), 278 (17) nm. Anal. Calcd for $C_{53}H_{92}FeKN_2O_8Si$: C, 63.13; H, 9.20; N, 2.78. Found: C, 62.65; H, 8.97; N, 2.92.

Preparation of [K(18-crown-6)(THF)$_2$][LFeBn] (5b). A mixture of **4b** (271 mg, 0.480 mmol) and 18-crown-6 (139 mg, 0.528 mmol) was dissolved in THF (8 mL), and the resulting red solution was cooled to –35 °C. Solid KC_8 (71 mg, 0.53 mmol) was added to the stirring red solution leading to a color change to green upon mixing. The reaction warmed to ambient temperature and was stirred for 90 minutes. The mixture was filtered through Celite yielding a green filtrate, and the filter was washed with THF (3 mL). The two filtrates were combined and concentrated under vacuum to 4 mL, layered with hexanes (5 mL), and placed in a –35 °C freezer overnight yielding green crystals of **5b** (376 mg, 77%). ^{1}H NMR (400 MHz, THF-d_8) δ 35 (4H), 19 (2H), 14 (2H), 3.3 (12H), 3.2 (24H), –5 (12H), –12 (2H), –19 (1H), –36 (2H), –141 (1H) ppm. Three resonances corresponding to 2H, 4H, and 6H were not found. These resonances may be broadened or hidden under solvent peaks. μ_{eff} (Evans, THF-d_8, 298 K) = 4.3(1) μ_B. Zero-field Mössbauer (solid, 80 K) δ = 0.27 mm s^{-1}, $|\Delta E_Q|$ = 1.75 mm s^{-1}, Γ_L = 0.38 mm s^{-1}, Γ_R = 0.34 mm s^{-1}. IR (ATR, neat, cm^{-1}): 3052 (w), 2959 (w), 2901 (m), 2875 (w), 2836 (w), 2807 (w), 1588 (m), 1480 (w), 1461 (w), 1449 (w), 1432 (m), 1385 (m), 1356 (m), 1325 (m), 1305 (w), 1301 (w), 1288 (w), 1256 (m), 1212 (w),

1177 (w), 1110 (s), 1062 (w), 968 (s), 941 (w), 927 (w), 888 (s), 849 (s), 840 (s), 808 (w), 795 (m), 756 (s), 744 (s), 715 (m), 695 (m), 592 (w), 584 (w), 540 (w), 532 (w), 522 (w), 432 (w), 417 (w). UV-vis (THF, ε in $mM^{-1}cm^{-1}$): 692 (3.6), 450 (4.4), 371 (8.0), 413 (5.5), 278 (24) nm. Anal. Calcd for $C_{56}H_{88}FeKN_2O_8$: C, 66.45; H, 8.76; N, 2.77. Found: C, 66.62; H, 8.49; N, 3.19.

Preparation of (OTf)$Me_2Si(CH_2)_2SiMe_2$(OTf) (6). In the dark, 1,2-bis(chlorodimethyl-silyl)ethane (176 mg, 0.818 mmol) was dissolved in toluene (3 mL) and cooled to –35 °C, alongside a solution of AgOTf (630 mg, 2.45 mmol) in toluene (10 mL). The cold silane solution was added dropwise to the stirring cold AgOTf solution, upon which a white precipitate formed immediately. The resulting mixture warmed to ambient temperature and was stirred for 18 h. Volatile materials were removed under vacuum leaving a white solid. The solid was extracted with pentane (30 mL) and filtered through Celite. Volatile materials were removed under vacuum, yielding colorless flakes of **6** (364 mg, 96%). ^1H NMR (400 MHz, C_6D_6) δ 0.46 (s, 4H, CH_2), 0.00 (s, 12H, CH_3) (lit: δ 0.90 and 0.52, $CDCl_3$; δ 0.74 and 0.38, DMSO-d_6) ppm.[9-10] ^{19}F NMR (376 MHz, C_6D_6) –77.2 (lit: δ –77.0) ppm.[9]

Preparation of LFe(CH_2SiMe_3)NN($Me_2Si(CH_2)_2SiMe_2$) (7a). A mixture of **5a** (157 mg, 0.145 mmol) and K(18-crown-6)($C_{10}H_8$) (63 mg, 0.14 mmol) was suspended in Et_2O (20 mL), giving a green mixture which turned magenta as it was frozen in a –196 °C cold well (corresponding to N_2 binding to form **5a-N$_2$**). A sample of **6** (64 mg, 0.14 mmol) was dissolved in Et_2O (5 mL), and this solution was frozen as well. Immediately upon thawing, the silane solution was added to the stirring red mixture of **5a-N$_2$** and K(18-crown-6)($C_{10}H_8$) using a cooled pipet (which was cooled by repeatedly sucking the thawing silane solution up and down), leading to an immediate color change to brown. The reaction mixture warmed to ambient temperature and was stirred for 20 minutes. Volatile materials were removed under vacuum to give a brown solid, which was then

extracted with toluene (12 mL) and filtered over Celite to give a brown filtrate. Volatile materials were removed under vacuum, and the resulting brown solid was dissolved in HMDSO (8 mL). The brown solution was concentrated under vacuum to 4 mL and placed in a –35 °C freezer overnight yielding brown crystals of **7a** (34 mg, 32%). ^1H NMR (500 MHz, C_6D_6) δ 27 (2H), 21 (2H), 16 (4H), 10 (9H), 6.6 (6H), 5.9 (2H), 5.2 (12H), 2.3 (6H), 0.3 (6H), –0.2 (6H), –12 (2H), –155 (1H) ppm. Three resonances corresponding to 6H, 2H, and 2H were not found. These resonances may be broadened or hidden under solvent peaks. μ_{eff} (Evans, C_6D_6, 298 K) = 5.0(2) μ_B. Zero-field Mössbauer (solid, 80 K) δ = 0.33 mm s^{-1}, $|\Delta E_Q|$ = 1.58 mm s^{-1}, Γ_L = 0.27 mm s^{-1}, Γ_R = 0.32 mm s^{-1}. IR (ATR, neat, cm^{-1}): 3070 (w), 2971 (m), 2932 (w), 2899 (w), 2879 (w), 1541 (w), 1521 (m), 1467 (m), 1440 (m), 1387 (m), 1369 (m), 1317 (s), 1251 (m), 1239 (m), 1196 (w), 1175 (m), 1151 (w), 1110 (m), 1060 (m), 1048 (m), 1019 (m), 964 (w), 956 (w), 935 (m), 914 (m), 857 (s), 838 (s), 816 (s), 793 (s), 758 (s), 729 (m), 715 (s), 670 (m), 656 (s), 616 (m), 604 (m), 559 (m), 540 (m), 524 (m), 489 (m), 440 (m), 413 (m), 403 (m). UV-vis (hexanes, ε in mM^{-1}cm^{-1}): 566 (1.2), 492 (2.2), 428 (6.7), 314 (23) nm. Anal. Calcd for $C_{39}H_{68}FeN_4Si_3$: C, 63.90; H, 9.35; N, 7.64. Found: C, 63.55; H, 9.37; N, 6.78. The found %N is significantly off from the calculated %N, and the reason is unknown.

Degradation of 7a. A mixture of **5a** (8.6 mg, 0.0080 mmol) and K(18-crown-6)($C_{10}H_8$) (3.4 mg, 0.0080 mmol) was suspended in Et_2O (2 mL), giving a green mixture which turned magenta as it was frozen in a –196 °C cold well (corresponding to N_2 binding to form **5a-N$_2$**). A sample of **6** (3.5 mg, 0.0080 mmol) was dissolved in Et_2O (1 mL), and this solution was frozen as well. Immediately upon thawing, the silane solution was added to the stirring red mixture of **5a-N$_2$** and K(18-crown-6)($C_{10}H_8$) using a cooled pipet, leading to an immediate color change to brown. The reaction mixture warmed to ambient temperature and was stirred for 10 minutes. Volatile materials

were removed under vacuum to give a brown residue, which was extracted with C_6D_6 (0.6 mL). The brown solution was transferred to a resealable NMR tube containing an internal $NiCp_2$ standard capillary and subsequently heated to 80 °C for 2 hours, leading to a color change to orange. Quantification by ^{1}H NMR spectroscopy showed the complete consumption of the *in situ* formed **7a** and the formation of **4a** (24%), **8** (29%), and the iron(II) amido complex $LFeN(Me_2Si(CH_2)_2SiMe_2)$ (36%) which was identified by X-ray diffraction on crystals grown from a scaled-up reaction. The mechanism of formation for the iron(II) amido complex $LFeN(Me_2Si(CH_2)_2SiMe_2)$ is unknown at this time. Trapping experiments with excess NC^tBu, PPh_3, or *trans*-stilbene gave no nitrene coupled products, arguing against the formation of an alkylnitrene intermediate.

Isolation of $LFeN(Me_2Si(CH_2)_2SiMe_2)$ from the degradation of 7a. A mixture of **5a** (88 mg, 0.081 mmol) and K(18-crown-6)($C_{10}H_8$) (35 mg, 0.081 mmol) was suspended in Et_2O (16 mL), giving a green mixture which turned magenta as it was frozen in a –196 °C cold well (corresponding to N_2 binding to form **5a-N₂**). A sample of **6** (36 mg, 0.081 mmol) was dissolved in Et_2O (2 mL), and this solution was frozen as well. Immediately upon thawing, the silane solution was added to the stirring red mixture of **5a-N₂** and K(18-crown-6)($C_{10}H_8$) using a cooled pipet, leading to an immediate color change to brown. The reaction mixture warmed to ambient temperature and was stirred for 15 minutes. Volatile materials were removed under vacuum to give a brown solid, which was then suspended in C_6H_6 (20 mL). The resulting brown suspension was heated to 80 °C for 90 minutes, during which time the color of the solution changed from brown to orange. Volatile materials were removed under vacuum, and the resulting brown solid was extracted with hexanes (10 mL) and filtered over Celite. The brown filtrate was concentrated under vacuum to 2 mL and placed in a –35 °C freezer overnight yielding brownish-yellow crystals of

$LFeN(Me_2Si(CH_2)_2SiMe_2)$ (12 mg). This complex was only partially characterized by ^1H NMR spectroscopy and X-ray crystallography. ^1H NMR (400 MHz, C_6D_6): δ 108 (4H), 78 (1H), 59 (6H), –1.9 (12H), –2.1 (4H), –3.3 (12H), –50 (2H), –57 (12H), –128 (4H) ppm.

Conversion of 5b to $LFeNN(Me_2Si(CH_2)_2SiMe_2)$ (8). A sample of **5b** (14.5 mg, 0.0143 mmol) was dissolved in Et_2O (3 mL), yielding a green solution that turned magenta as it was frozen in –196 °C cold well (corresponding to N_2 binding to form **5b-N_2**). A sample of **6** (3.2 mg, 0.0072 mmol) was dissolved in Et_2O (1 mL), and this solution was frozen as well. Immediately upon thawing, the silane solution was added to the stirring red solution of **5b-N_2** using a cooled pipet, leading to an immediate color change from red to brown. The reaction mixture warmed to ambient temperature and was stirred for 1 hour, giving an orange solution. Volatile materials were removed under vacuum to give a yellow residue, which was then extracted with C_6D_6 (0.6 mL). Quantification by ^1H NMR spectroscopy (comparing to an external $NiCp_2$ capillary) showed the formation of **4b** (64%) and **8** (22%).

Independent Synthesis of 8. A sample of **9** (309 mg, 0.313 mmol) was dissolved in Et_2O (12 mL), yielding a green solution that turned magenta as it was frozen in a –196 °C cold well. A sample of 1,2-bis(chlorodimethylsilyl)ethane (74 mg, 0.34 mmol) was dissolved in Et_2O (3 mL), and this solution was frozen as well. Immediately upon thawing, the silane solution was added to the stirring red solution of **9** using a cooled pipet, leading to an immediate color change to brown. The reaction mixture warmed to ambient temperature and was stirred for 50 minutes. Volatile materials were removed under vacuum to give a brown solid. The solid was extracted with HMDSO (40 mL) and filtered over Celite. The resulting brown filtrate was concentrated to 15 mL and placed in a –35 °C freezer overnight yielding a mixture of brown and yellow solids. The supernatant was decanted, and the solids were triturated with HMDSO (3 x 1 mL). The remaining solids were

dissolved in hexanes (2 mL) and filtered through Celite. The brown filtrate was placed in a –35 °C freezer overnight yielding brown crystals of **8** (30 mg, 22%). ^{1}H NMR (400 MHz, C_6D_6) spectra can be assigned as a mixture of the two spin isomers: Spin isomer #1: δ 52 (1H), 10.3 (12H), 1.9 (4H), –2.4 (2H), –3.7 (6H), –3.8 (4H), –5.7 (12H), –27 (12H), –34 (4H) ppm. Spin isomer #2: δ 16 (4H), 11.5 (6H), 11.3 (2H), 10.8 (12H), –0.3 (12H), –3.2 (12H), –52 (1H), –54 (4H) ppm. One resonance corresponding to 4H was not found for spin isomer #2. This resonance may be broadened or hidden under solvent peaks. μ_{eff} (Evans, C_6D_6, 298 K) = 2.9(1) μ_B. Zero-field Mössbauer (solid, 80 K) Spin isomer #1: δ = 0.13 mm s^{-1}, $|\Delta E_Q|$ = 2.13 mm s^{-1}, Γ_L = 0.50 mm s^{-1}, Γ_R = 0.36 mm s^{-1}. Spin isomer #2: δ = 0.51 mm s^{-1}, $|\Delta E_Q|$ = 1.75 mm s^{-1}, Γ_L = 0.50 mm s^{-1}, Γ_R = 0.39 mm s^{-1}. IR (ATR, neat, cm^{-1}): 3068 (w), 2969 (m), 2936 (w), 2904 (w), 2877 (w), 2811 (w), 1630 (w), 1588 (w), 1531 (w), 1465 (w), 1440 (m), 1383 (s), 1321 (s), 1251 (s), 1202 (w), 1180 (w), 1151 (w), 1128 (w), 1106 (w), 1060 (w), 1003 (m), 953 (s), 898 (m), 877 (m), 845 (s), 824 (s), 795 (s), 771 (s), 760 (s), 699 (m), 672 (m), 637 (w), 621 (m), 563 (w), 549 (w), 540 (w), 526 (w), 520 (w), 503 (w), 475 (w), 440 (m). UV-vis (hexanes, ε in mM^{-1}cm^{-1}): 480 (1.2), 437 (1.7), 401 (2.9), 319 (14), 287 (12) nm. Anal. Calcd for $C_{37}H_{57}FeN_4Si_2$: C, 65.09; H, 8.90; N, 8.67. Found: C, 60.98; H, 8.34; N, 5.97. The microanalytical data are significantly off from the calculated values, which may indicate impurities.

Conversion of 5c to LFeN($Me_2Si(CH_2)_2SiMe_2$)NMe (10). A sample of **5c** (9.9 mg, 0.011 mmol) was dissolved in Et_2O (2 mL), yielding a green solution that turned red as it was frozen in a –196 °C cold well (corresponding to N_2 binding to form **5c-N$_2$**). A sample of **6** (2.5 mg, 0.0056 mmol) was dissolved in Et_2O (1 mL), and this solution was frozen as well. Immediately upon thawing, the silane solution was added to the stirring red solution of **5c-N$_2$** using a cooled pipet, leading to an immediate color change from red to brown. The reaction mixture warmed to ambient

temperature and was stirred for 50 minutes, giving a yellow solution. Volatile materials were removed under vacuum to give a yellow residue, which was then extracted with C_6D_6 (0.5 mL). Quantification by ^1H NMR spectroscopy (comparing to a $NiCp_2$ capillary) showed the formation of **4c** (57%) and **10** (37%).

Independent Synthesis of 10. A solution of 1,2-bis(chlorodimethylsilyl)ethane (249.6 mg, 1.160 mmol) in Et_2O (1 mL) was added dropwise to a solution of methylhydrazine (183 μL, 3.48 mmol) in Et_2O (1 mL), leading to the immediate formation of a white precipitate. The reaction mixture was heated to 50 °C and stirred vigorously for 21 hours. The white solid was filtered off using a glass frit, and the solid was washed with Et_2O (5 mL). The two colorless filtrates were combined, and n-BuLi (0.82 mL, 1.3 mmol) was added dropwise leading to an immediate color change to yellow. The reaction mixture was stirred for 1 hour, during which the solution became cloudy. Volatile materials were removed under vacuum to give a yellow oil which was then redissolved in pentane (5 mL). Volatile materials were once again removed under vacuum to give a yellow oil (^1H NMR spectrum is consistent with the assignment of $LiN(Me_2Si(CH)_2SiMe_2)NMe$) which was used without further purification. A sample of this oil (123.5 mg, 0.64 mmol) was dissolved in toluene (4 mL) and added dropwise to a stirring orange suspension of $[LFeBr]_2$ (234.0 mg, 0.21 mmol) in toluene (10 mL), leading to an immediate color change to yellow-brown. The reaction was stirred at ambient temperature for 3 hours. Volatile materials were removed under vacuum to give a yellow-brown solid. The solid was extracted with pentane (10 mL) and filtered over Celite. The resulting yellow-brown filtrate was concentrated under vacuum to 2 mL and placed in a –35 °C freezer for 1 day, yielding yellow crystalline **10** (58.8 mg, 21%). ^1H NMR (500 MHz, C_6D_6) δ 167 (3H), 129 (1H), 106 (2H), 61 (6H), –10 (12H), –16 (4H), –59 (2H), –86 (12H) ppm. Four resonances corresponding to 2H, 4H, 6H, and 6H were not found. These resonances may be

broadened or hidden under solvent peaks. μ_{eff} (Evans, C_6D_6, 298 K) = 5.1(2) μ_B. Zero-field Mössbauer (solid, 80 K): as described below (Fig. S21-22), there were two alternative fits. The three-component fit is given here. η^2 $S = 2$ isomer (9%): δ = 0.98 mm s^{-1}, $|\Delta E_Q|$ = 1.45 mm s^{-1}; η^2 $S = 1$ isomer (37%): δ = 0.55 mm s^{-1}, $|\Delta E_Q|$ = 1.28 mm s^{-1}; η^1 $S = 2$ isomer (54%): δ = 0.68 mm s^{-1}, $|\Delta E_Q|$ = 0.96 mm s^{-1}. IR (ATR, neat, cm^{-1}): 3068 (w), 2969 (m), 2936 (m), 2912 (m), 2877 (m), 2828 (w), 2780 (w), 1529 (m), 1465 (m), 1385 (s), 1319 (m), 1284 (m), 1264 (m), 1249 (m), 1198 (m), 1180 (m), 1103 (m), 1081 (m), 1060 (m), 1025 (m), 972 (m), 941 (m), 873 (m), 824 (s), 795 (m), 777 (m), 760 (s), 703 (m), 684 (m), 637 (m), 602 (m), 586 (m), 462 (m) 446 (m), 417 (m). UV-vis (hexanes, ε in mM^{-1}cm^{-1}): 486 (0.61), 463 (0.91), 405 (2.9), 379 (6.2), 333 (12), 280 (8.3) nm. Anal. Calcd for $C_{36}H_{60}FeN_4Si_2$ • 0.7 C_5H_{12} (pentane): C, 64.61; H, 9.39; N, 7.63. Found: C, 64.53; H, 9.04; N, 7.44.

Preparation of LFeN(Ph)N(Me$_2$Si(CH$_2$)$_2$SiMe$_2$) 12. A mixture of **1** (91 mg, 0.098 mmol) and K(18-crown-6)($C_{10}H_8$) (42 mg, 0.098 mmol) were suspended in Et_2O (12 mL), yielding a green mixture that turned red-purple as it was frozen in a –196 °C cold well (corresponding to N_2 binding to form **1-N$_2$**). A sample of **6** (43 mg, 0.098 mmol) was dissolved in Et_2O (2 mL), and this solution was frozen as well. Immediately upon thawing, the silane solution was added to the stirring red mixture of **1-N$_2$** and K(18-crown-6)($C_{10}H_8$) with a cooled pipet, leading to an immediate color change to brown. The reaction mixture warmed to ambient temperature and was stirred for 25 minutes. Volatile materials were removed under vacuum to give a reddish-brown residue, which was then extracted with pentane (10 mL) and filtered over Celite. The red filtrate was concentrated under vacuum to 3 mL and filtered over Celite once more, and the filter was washed with pentane (2 mL). The two filtrates were combined and concentrated under vacuum to 4 mL and and placed in a –35 °C freezer overnight yielding red crystals of **12** (32 mg, 45%). ^{1}H NMR (400 MHz, C_6D_6)

δ 61 (6H), 56 (6H), 48 (1H), 41 (1H), 39 (2H), 34 (6H), 28 (2H), –0.9 (2H), –4 (2H), –21 (6H), –28 (6H), –47 (2H), –70 (6H), –73 (6H), –91 (1H), –188 (2H) ppm. Four resonances corresponding to 4H, 1H, 1H, and 1H were not found. These resonances may be broadened or hidden under solvent peaks. There were small peaks corresponding to apparent impurities in the proton NMR spectrum. μ_{eff} (Evans, C_6D_6, 298 K) = 5.7(2) μ_B. Zero-field Mössbauer (solid, 80 K) δ = 0.68 mm s^{-1}, $|\Delta E_Q|$ = 1.40 mm s^{-1}, Γ_L = 0.40 mm s^{-1}, Γ_R = 0.46 mm s^{-1}. IR (ATR, neat, cm^{-1}): 3070 (w), 2969 (m), 2936 (w), 2918 (w), 2908 (w), 2879 (w), 2809 (w), 1607 (w), 1588 (w), 1566 (w), 1523 (m), 1488 (w), 1467 (m), 1440 (m), 1385 (s), 1364 (s), 1317 (s), 1299 (w), 1280 (w), 1249 (s), 1200 (w), 1177 (m), 1147 (w), 1110 (m), 1101 (m), 1071 (w), 1023 (m), 984 (w), 966 (w), 933 (s), 869 (m), 847 (s), 808 (m), 795 (s), 785 (s), 760 (s), 750 (s), 721 (m), 627 (m), 602 (w), 565 (w), 551 (w), 520 (w), 495 (w), 479 (w), 442 (m), 419 (w), 401 (s). UV-vis (hexanes, ε in $mM^{-1}cm^{-1}$): 493 (1.1), 452 (2.5), 378 (9.6), 328 (13), 300 (12), 269 (14) nm. Anal. Calcd for $C_{41}H_{62}FeN_4Si_2$: C, 68.11; H, 8.64; N, 7.75. Found: C, 67.63; H, 8.43; N, 7.29.

NMR Spectroscopy

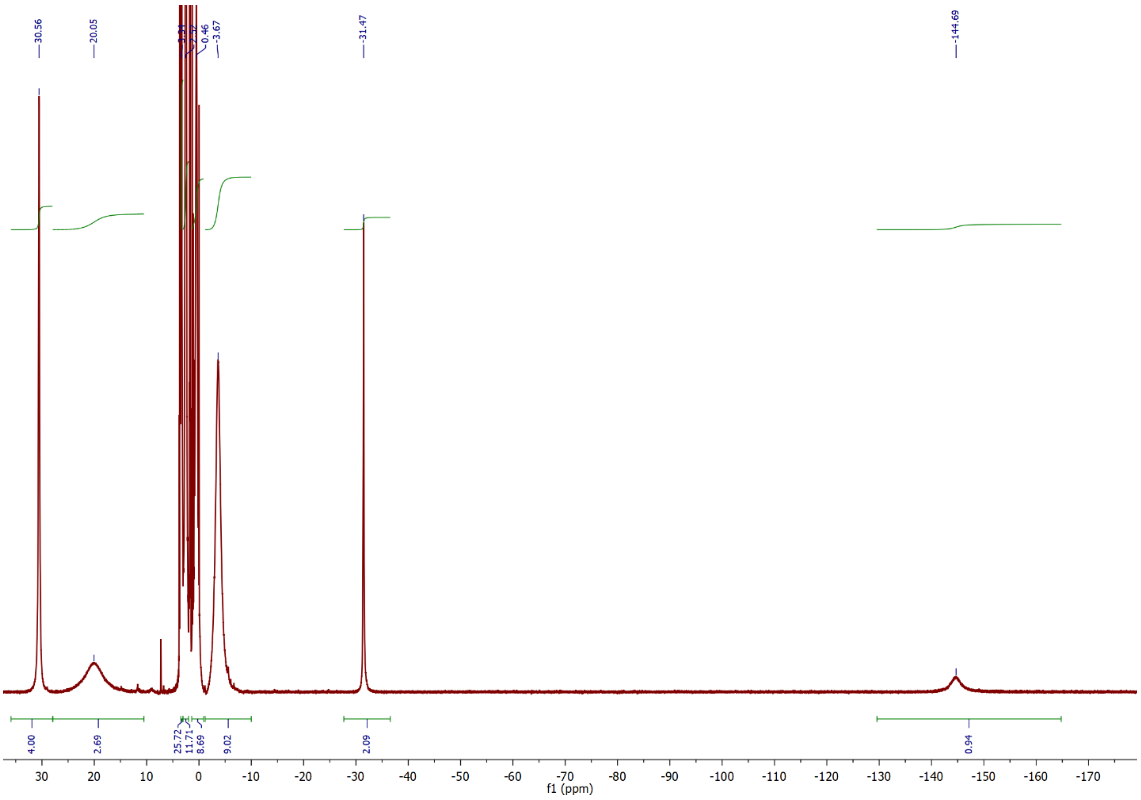

Figure S1. ^{1}H NMR spectrum of [K(18-crown-6)(THF)$_2$][LFeCH$_2$SiMe$_3$] (**5a**) in THF-d_8 at 298 K.

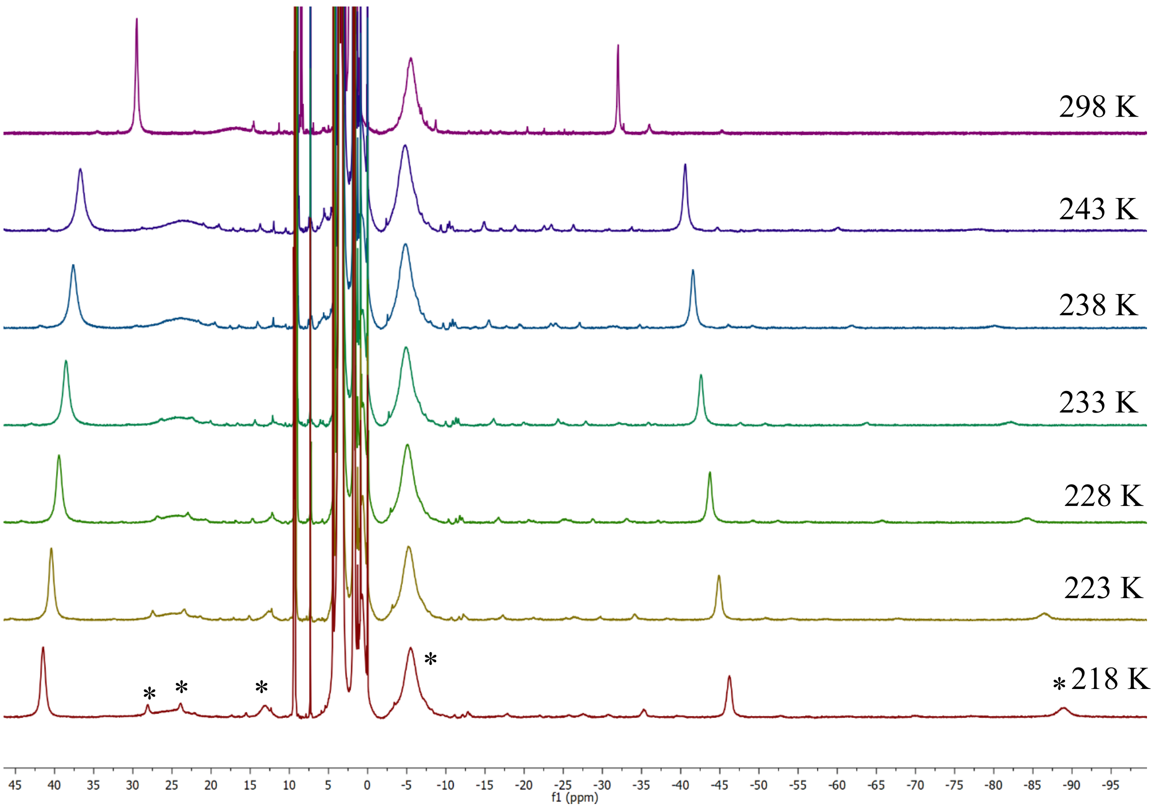

Figure S2. Variable-temperature ^1H NMR spectra of [K(18-crown-6)(THF)$_2$][LFeCH$_2$SiMe$_3$] (**5a**) in THF-d_8 under an N_2 atmosphere ranging from 298 K to 218 K. Peaks corresponding to the N_2 bound complex are denoted with an asterisk.

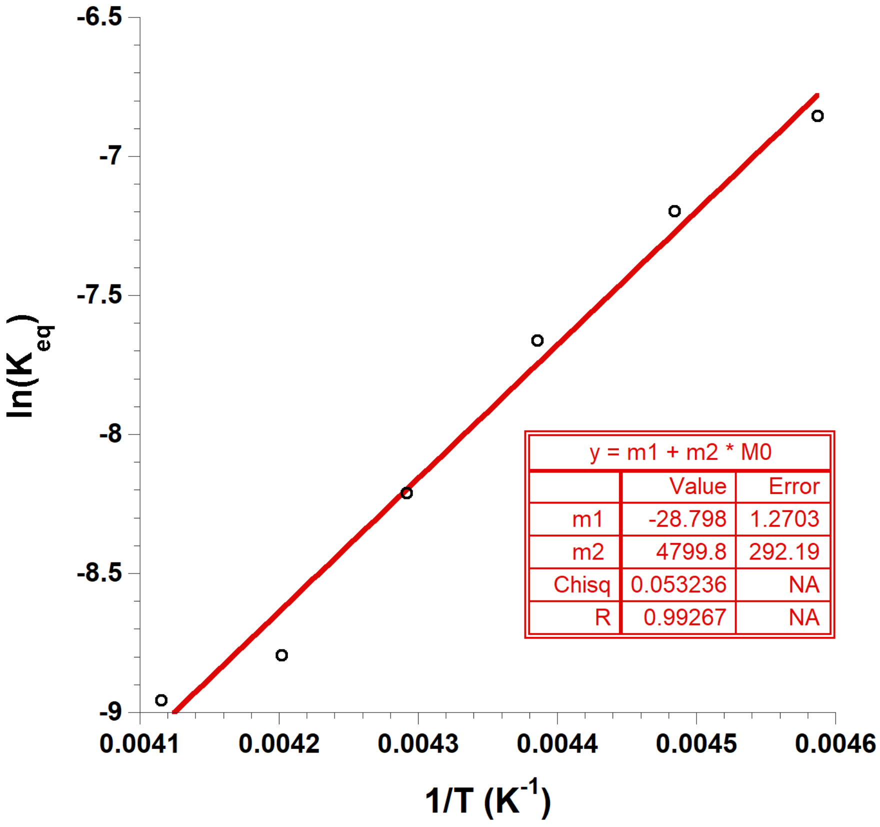

Figure S3. Van't Hoff plot for N_2 binding to [K(18-crown-6)(THF)$_2$][LFeCH$_2$SiMe$_3$] (**5a**) to form [K(18-crown-6)][LFe(N$_2$)CH$_2$SiMe$_3$] (**5a-N$_2$**) with points fit to a linear function. The solution concentration of N_2 was calculated using a literature equation,[11] additionally accounting for the pressure changes with temperature in the closed system. The concentrations of **5a** and **5a-N$_2$** were determined by integration relative to a nickelocene capillary using the ^{1}H NMR spectra shown in Figure S2.

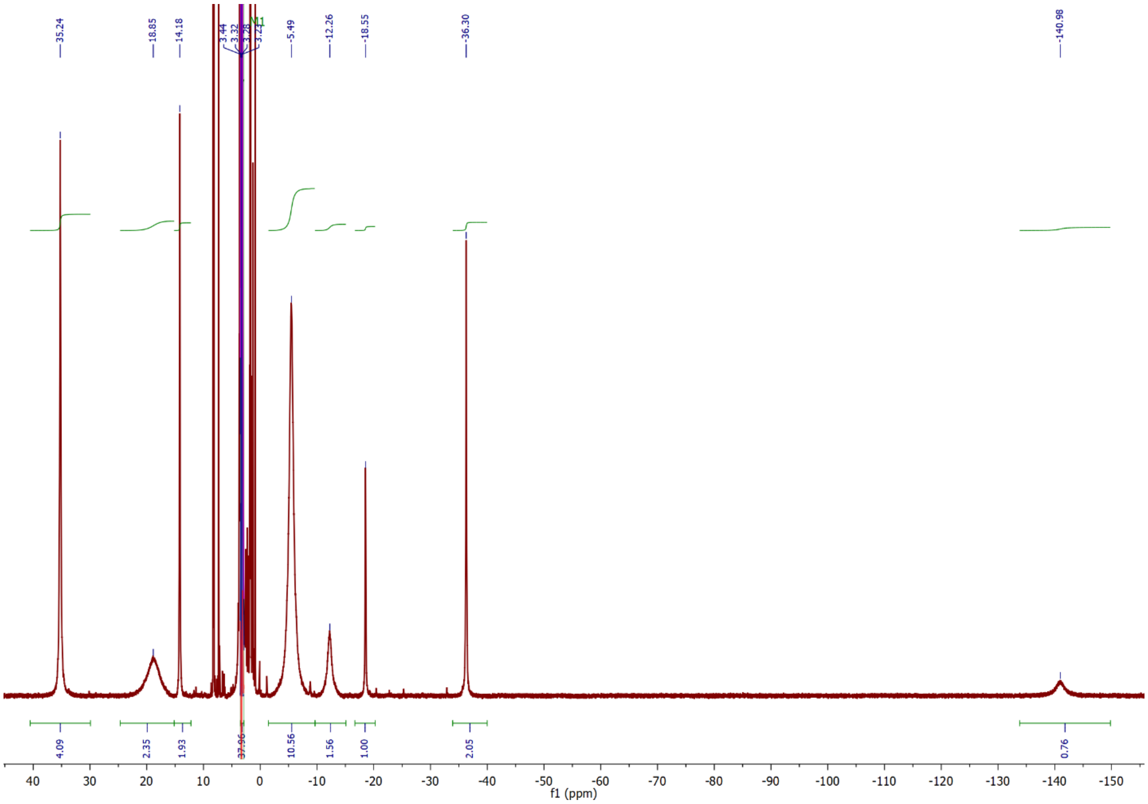

Figure S4. ^{1}H NMR spectrum of [K(18-crown-6)(THF)$_2$][LFeBn] (**5b**) in THF-d_8 at 298 K.

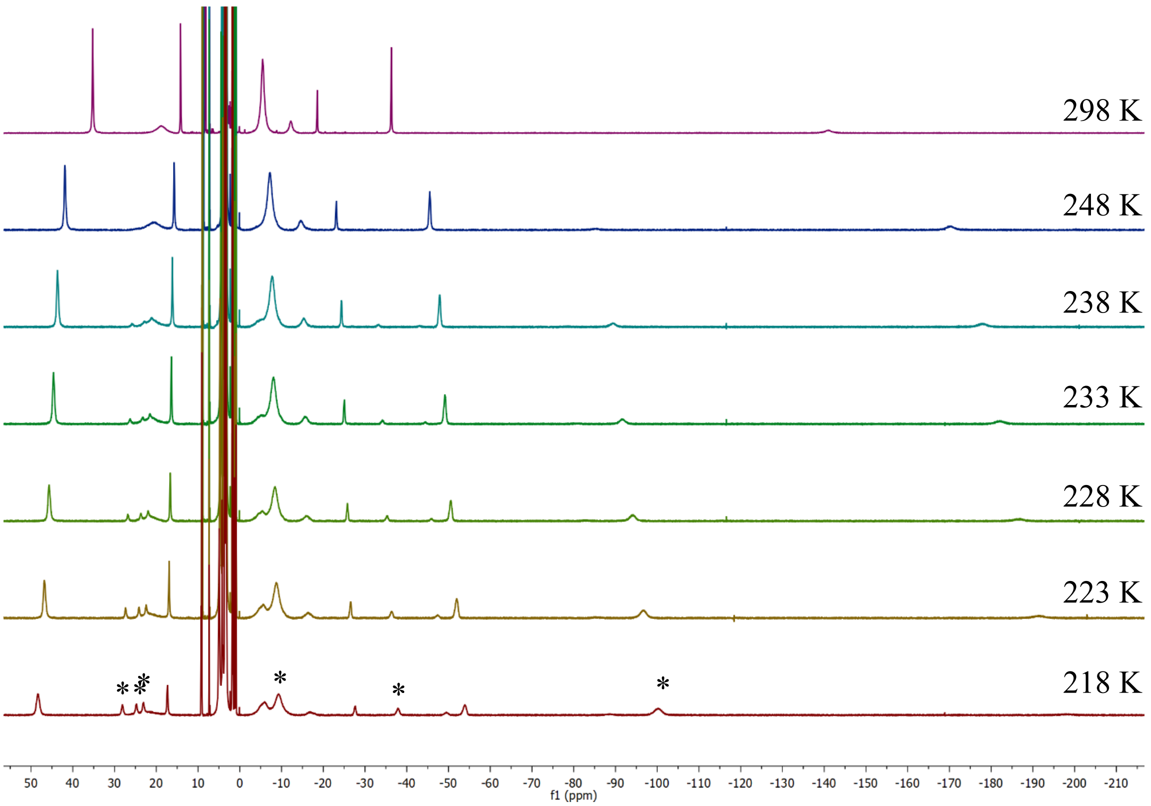

Figure S5. Variable-temperature ^{1}H NMR spectra of [K(18-crown-6)(THF)$_2$][LFeBn] (**5b**) in THF-d_8 under an N_2 atmosphere ranging from 298 K to 218 K. Peaks corresponding to the N_2 bound complex are denoted by an asterisk.

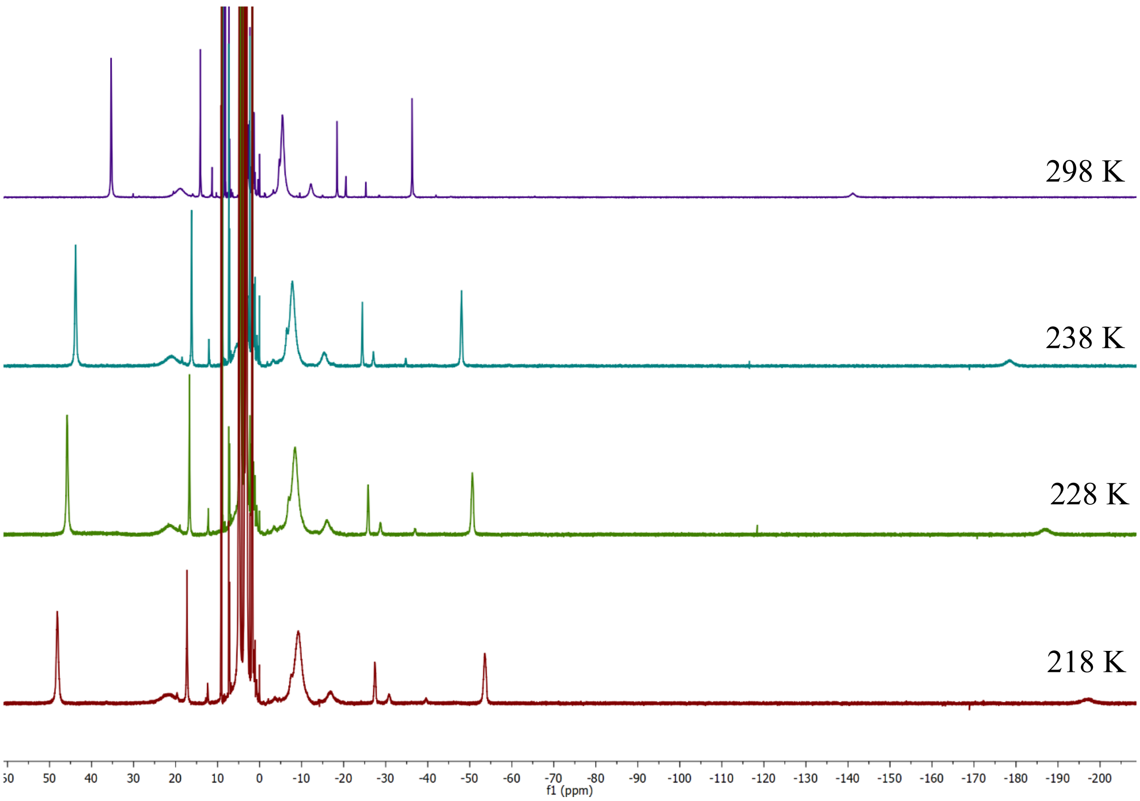

Figure S6. Variable-temperature ^{1}H NMR spectra of [K(18-crown-6)(THF)$_2$][LFeBn] (**5b**) in THF-d_8 under an Ar atmosphere ranging from 298 K to 218 K.

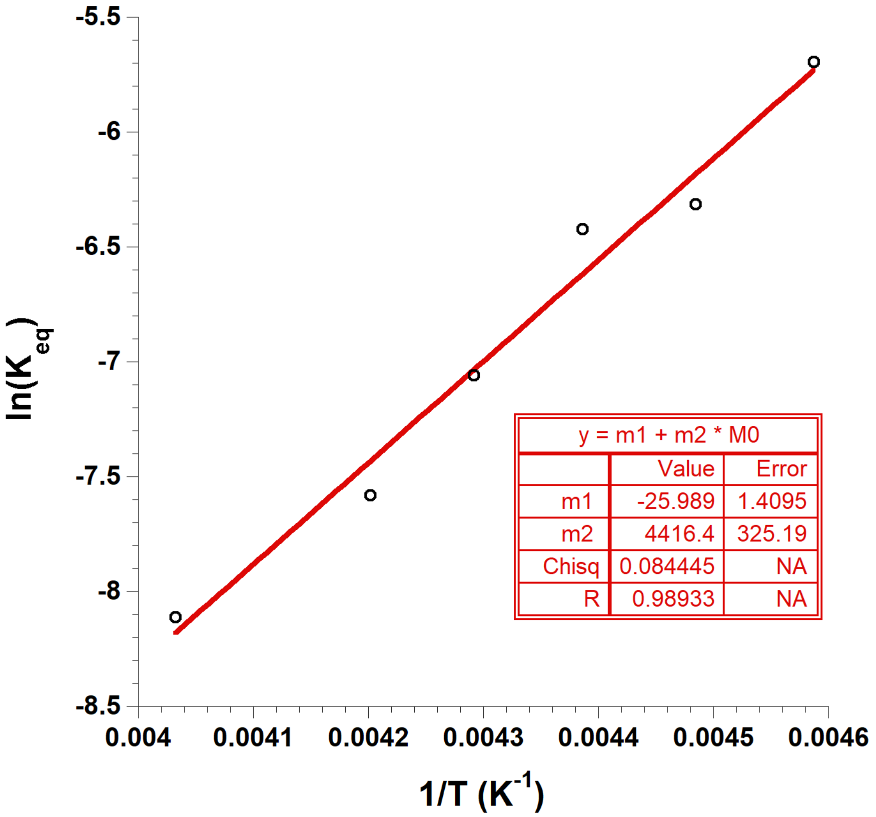

Figure S7. Van't Hoff plot for N_2 binding to [K(18-crown-6)(THF)$_2$][LFeBn] (**5b**) to form [K(18-crown-6)][LFe(N_2)Bn] (**5b-N$_2$**) with points fit to a linear function. The solution concentration of N_2 was calculated using a literature equation,[11] additionally accounting for the pressure changes with temperature in the closed system. The concentrations of **5b** and **5b-N$_2$** were determined by integration relative to a nickelocene capillary using the ^{1}H NMR spectra shown in Figure S5.

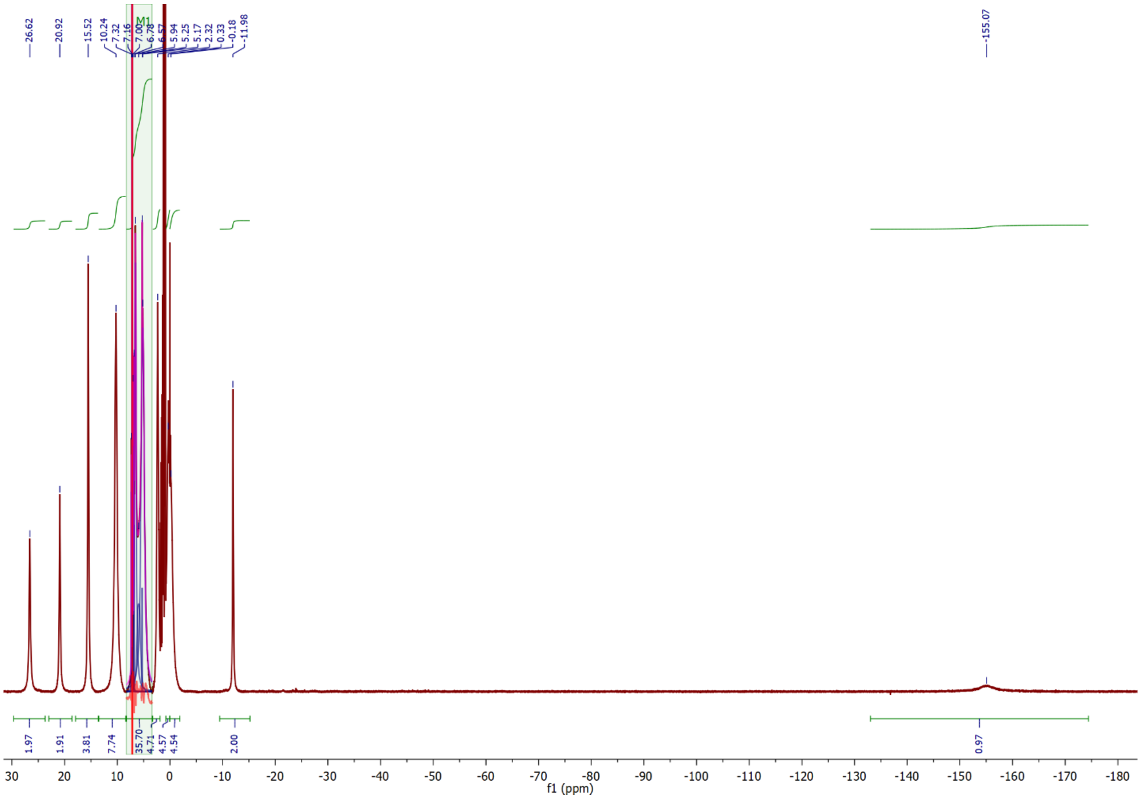

Figure S8. ^{1}H NMR spectrum of $LFe(CH_2SiMe_3)NN(Me_2Si(CH_2)_2SiMe_2)$ (**7a**) in C_6D_6 at 298 K.

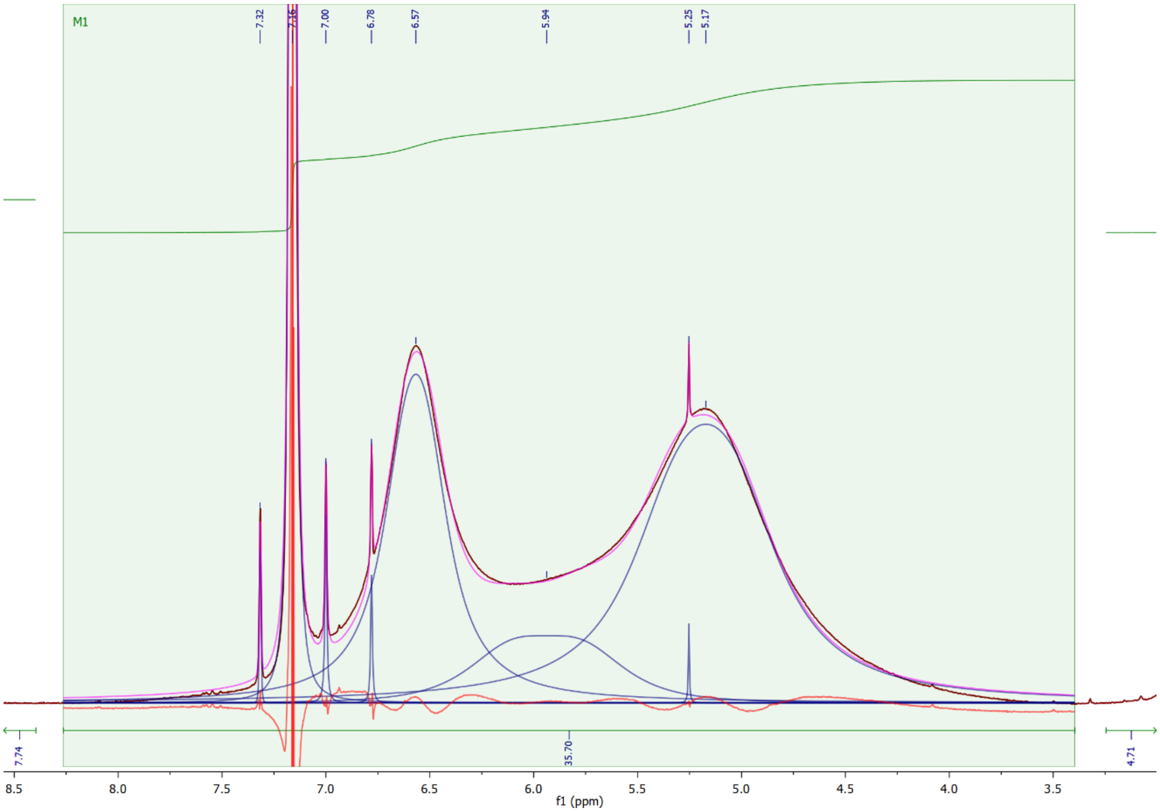

Figure S9. Inset of the ^{1}H NMR spectrum of **7a** in Figure S8 showing the region with overlapping peaks.

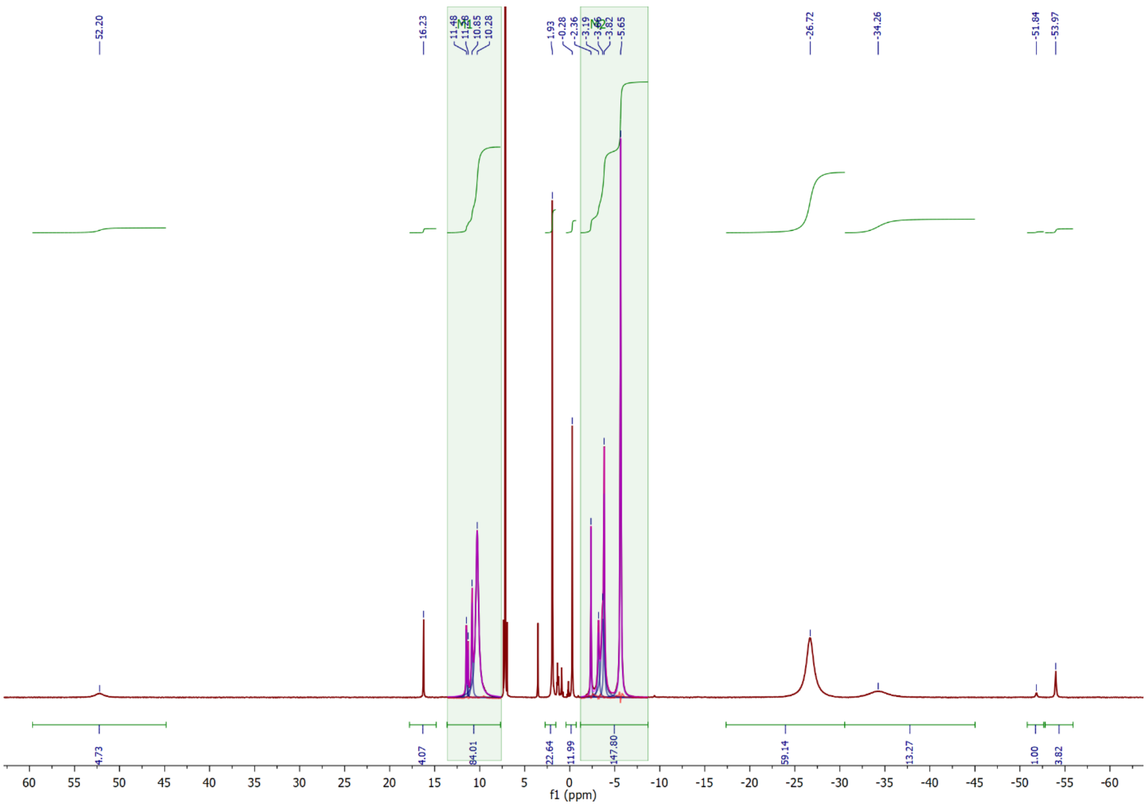

Figure S10. ^{1}H NMR spectrum of LFeNN($Me_2Si(CH_2)_2SiMe_2$) (**8**) in C_6D_6 at 298 K.

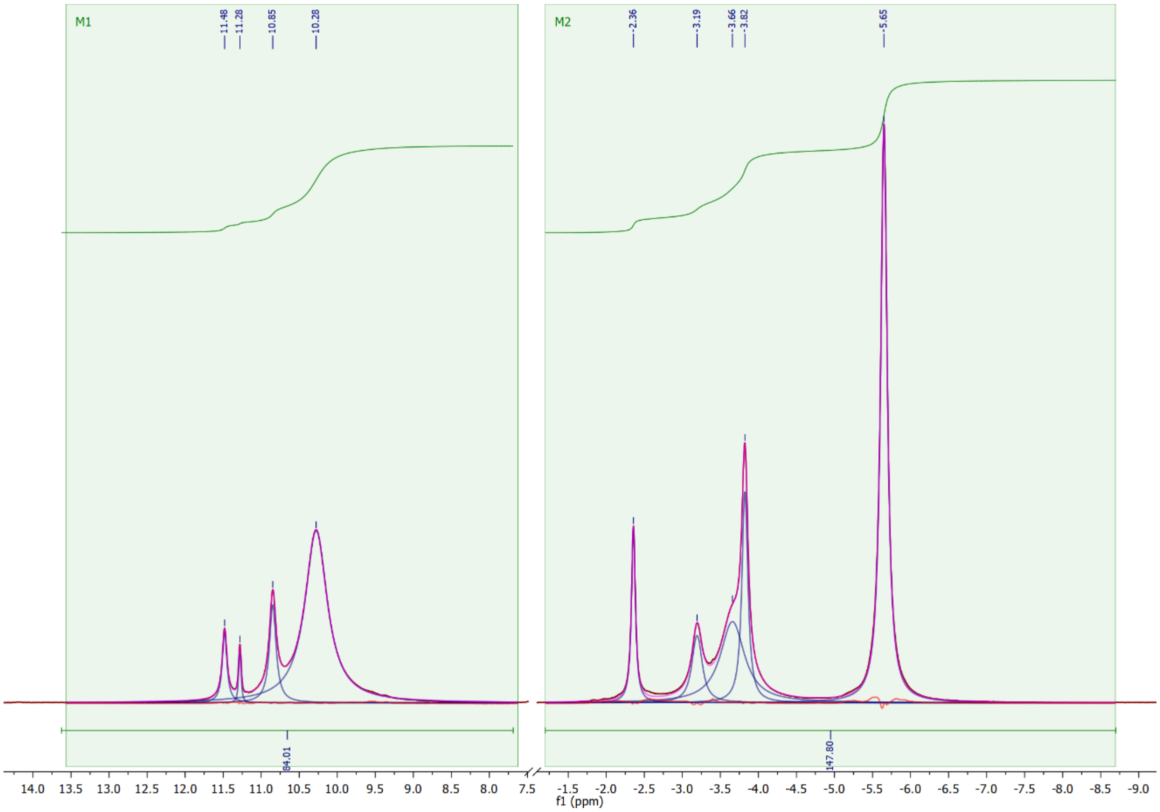

Figure S11. Inset of the ^{1}H NMR spectrum of **8** in Figure S10 showing the regions with overlapping peaks.

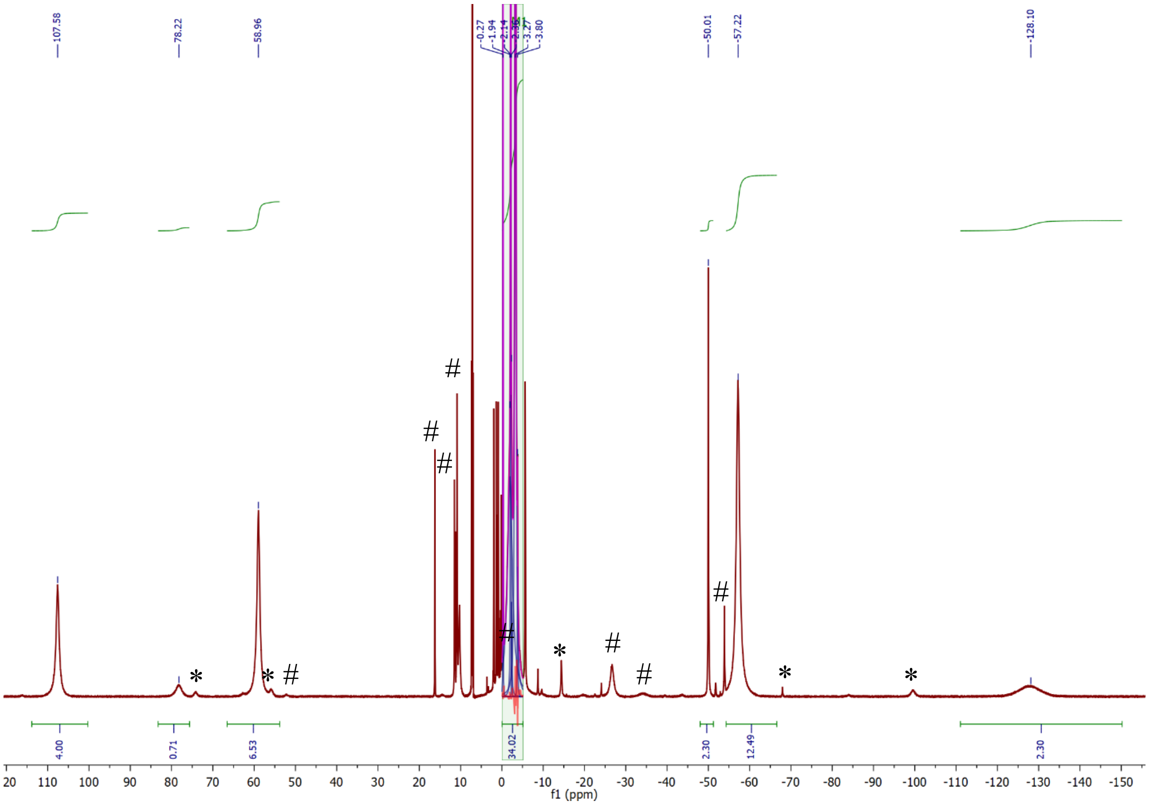

Figure S12. ^{1}H NMR of $LFeN(Me_2Si(CH_2)_2SiMe_2)$ in C_6D_6 at 298 K. Peaks corresponding to known impurities are denoted by * (**4a**) or # (**8**).

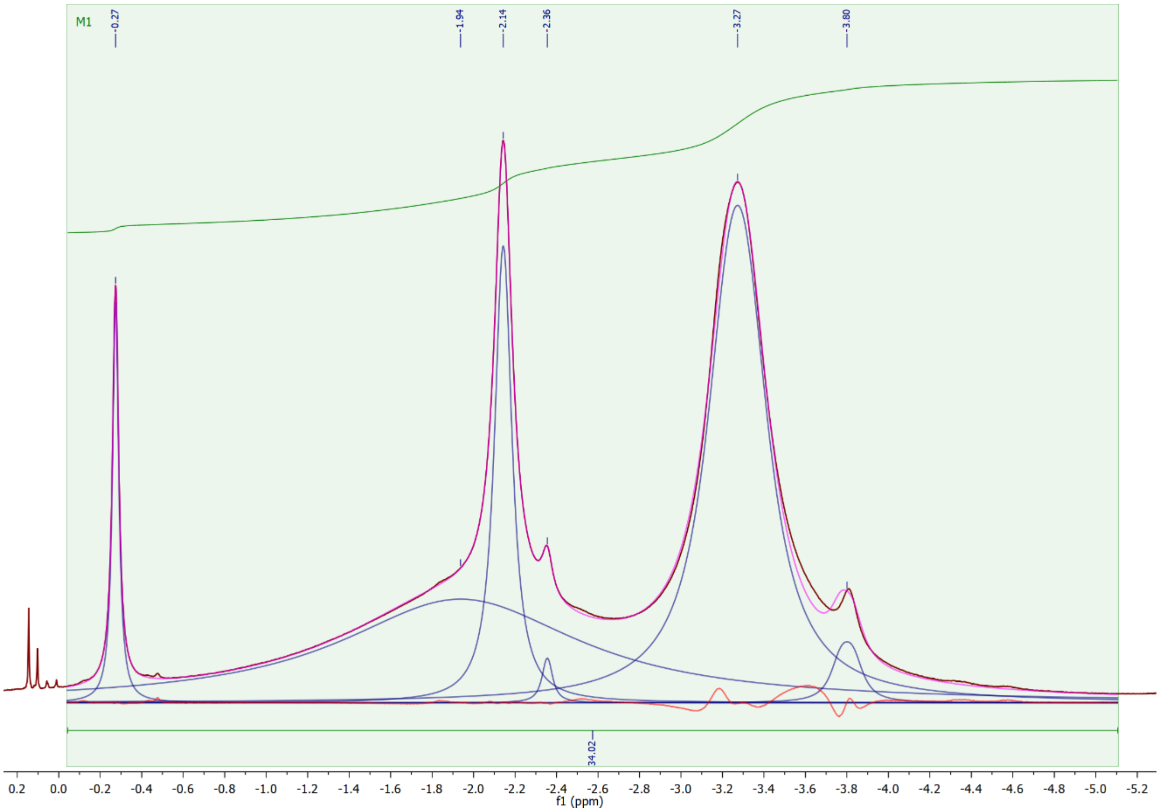

Figure S13. Inset of the ^{1}H NMR spectrum of LFeN($Me_2Si(CH_2)_2SiMe_2$) in Figure S12 showing the region with overlapping peaks.

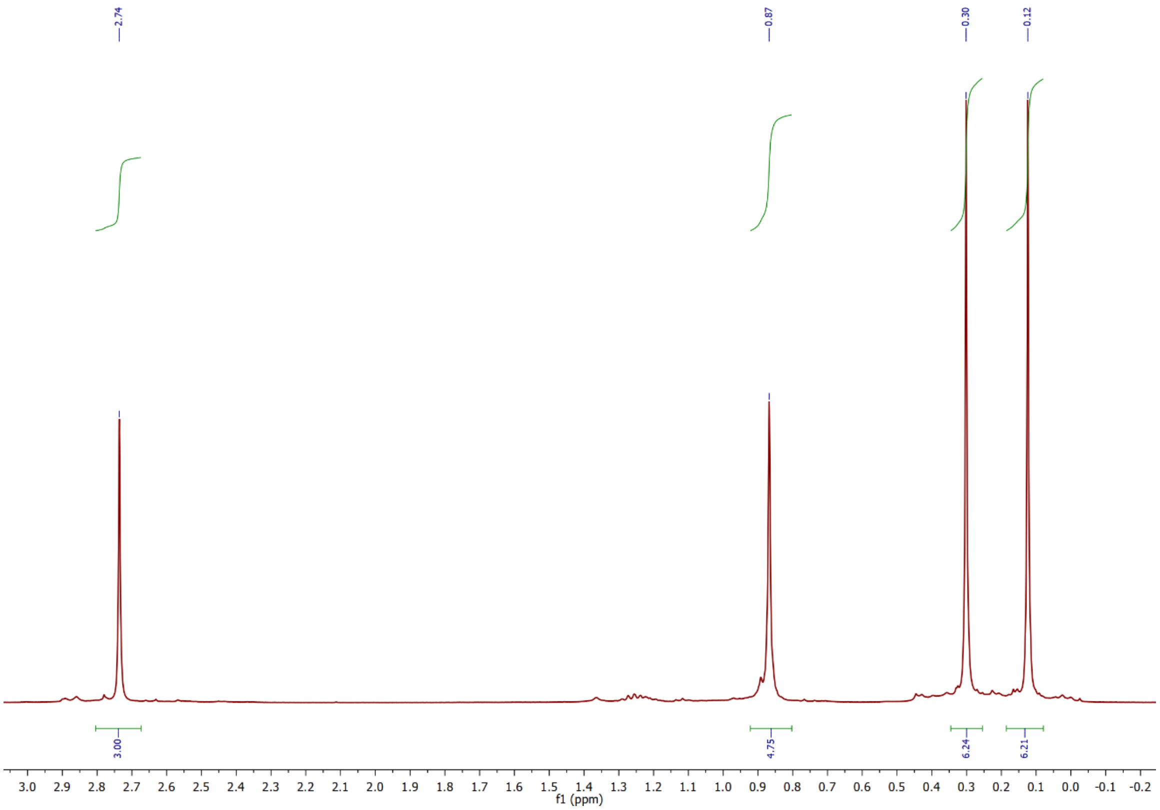

Figure S14. ^{1}H NMR spectrum of crude LiN($Me_2Si(CH_2)_2SiMe_2$)NMe in C_6D_6 at 298 K.

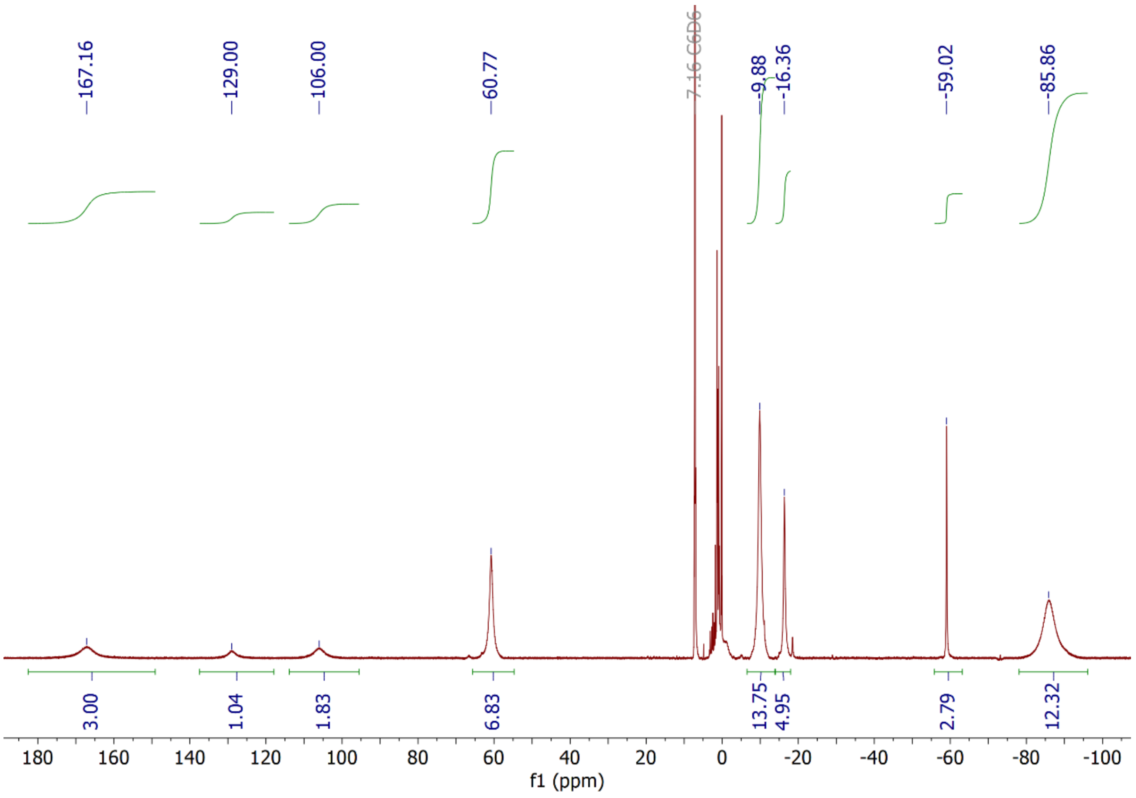

Figure S15. ^{1}H NMR spectrum of $LFeN(Me_2Si(CH_2)_2SiMe_2)NMe$ (**10**) in C_6D_6 at 298 K.

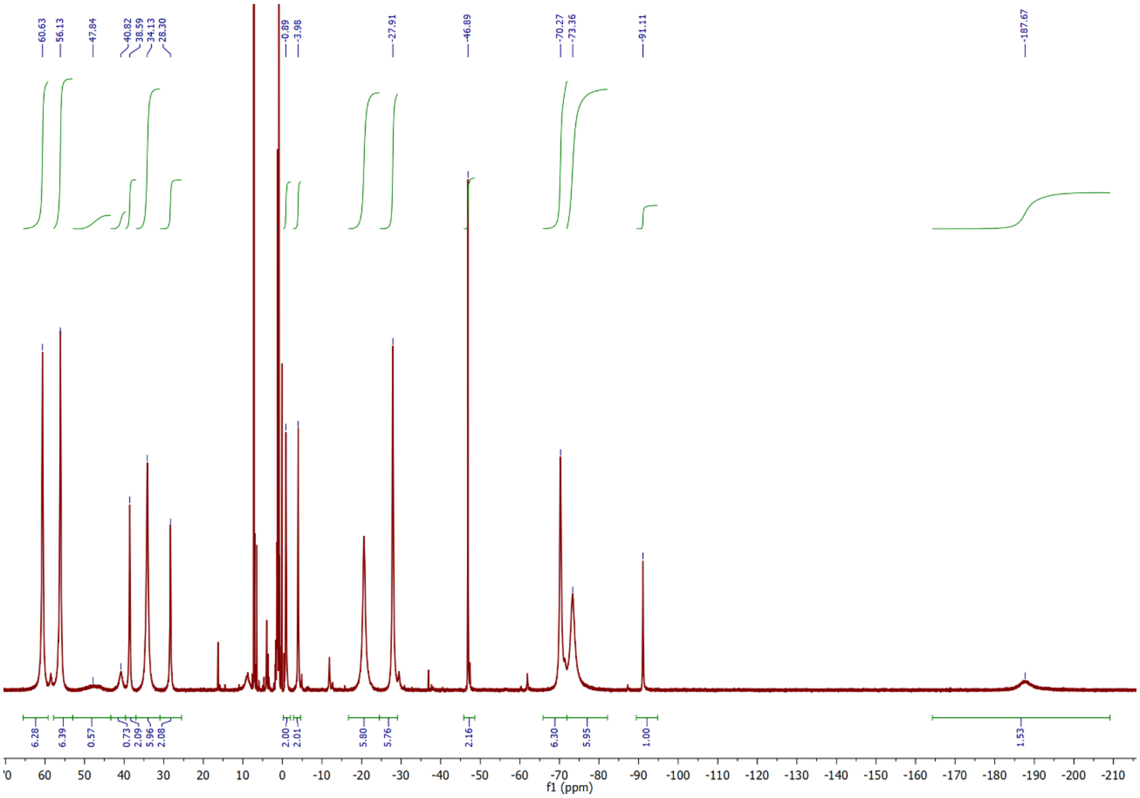

Figure S16. ^{1}H NMR spectrum of LFeN(Ph)N($Me_2Si(CH_2)_2SiMe_2$) (**12**) in C_6D_6 at 298 K.

Mössbauer Spectroscopy

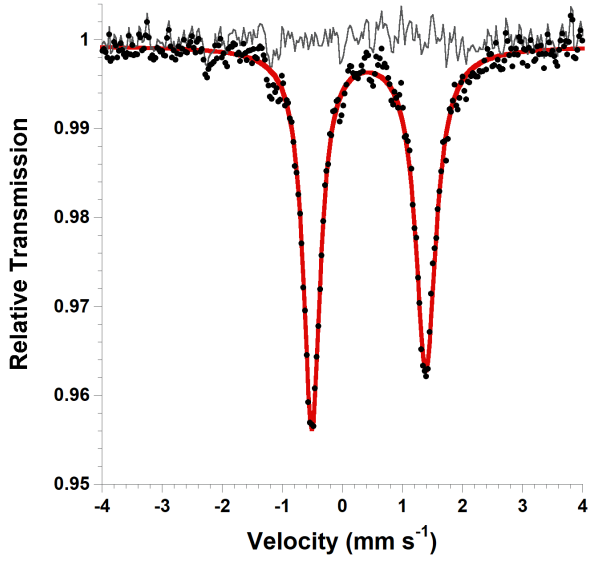

Figure S17. Solid state zero-field Mössbauer spectrum of [K(18-crown-6)(THF)$_2$][LFeCH$_2$SiMe$_3$] (**5a**) where the black circles are the data, the red line is a one-component simulation with parameters δ = 0.44 mm s^{-1}, $|\Delta E_Q|$ = 1.90 mm s^{-1}, Γ_L = 0.35 mm s^{-1}, Γ_R = 0.41 mm s^{-1}, and the gray line is the residual.

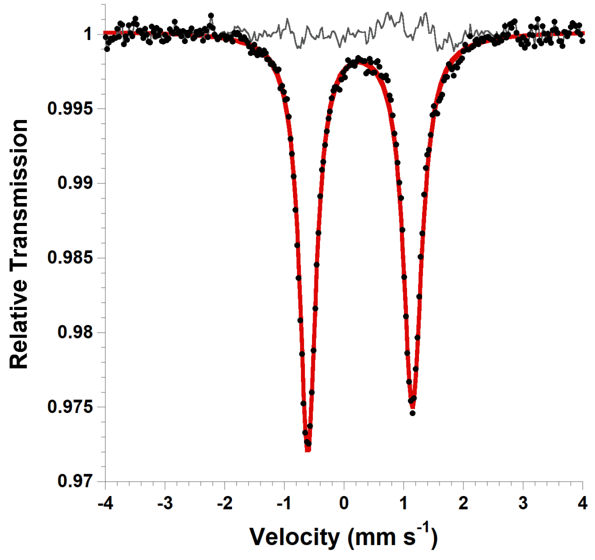

Figure S18. Solid state zero-field Mössbauer spectrum of [K(18-crown-6)(THF)$_2$][LFeBn] (**5b**) where the black circles are the data, the red line is a one-component simulation with parameters δ

$= 0.27$ mm s^{-1}, $|\Delta E_Q| = 1.75$ mm s^{-1}, $\Gamma_L = 0.38$ mm s^{-1}, $\Gamma_R = 0.34$ mm s^{-1}, and the gray line is the residual.

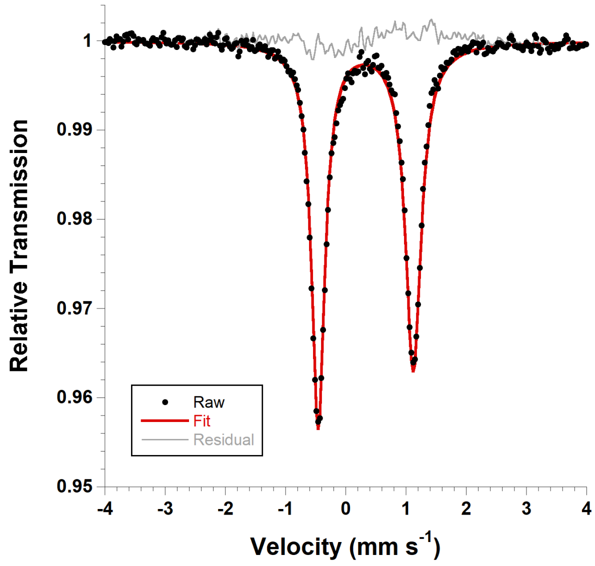

Figure S19. Solid state zero-field Mössbauer spectrum of $LFe(CH_2SiMe_3)NN(Me_2Si(CH_2)_2SiMe_2)$ (**7a**) where the black circles are the data, the red line is a one-component simulation with parameters $\delta = 0.33$ mm s^{-1}, $|\Delta E_Q| = 1.58$ mm s^{-1}, $\Gamma_L = 0.27$ mm s^{-1}, $\Gamma_R = 0.32$ mm s^{-1}, and the gray line is the residual.

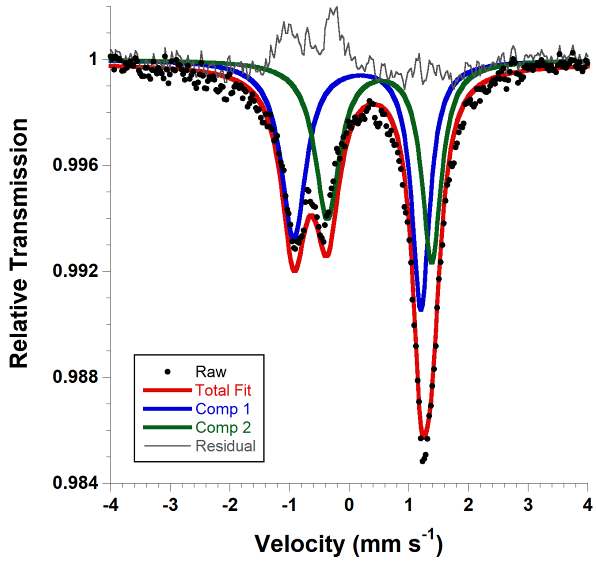

Figure S20. Solid state zero-field Mössbauer spectrum of $LFeNN(Me_2Si(CH_2)_2SiMe_2)$ (**8**) where the black circles are the data, the red line is a two-component simulation, and the gray line is the residual. The blue line is a simulation for Component 1 ($S = 1/2$) with parameters $\delta = 0.13$ mm s^{-1}, $|\Delta E_Q| = 2.13$ mm s^{-1}, $\Gamma_L = 0.50$ mm s^{-1}, $\Gamma_R = 0.36$ mm s^{-1}, and relative area = 53%. The green

line is a simulation for Component 2 (S = 3/2) with parameters δ = 0.51 mm s^{-1}, $|\Delta E_Q|$ = 1.75 mm s^{-1}, Γ_L = 0.50 mm s^{-1}, Γ_R = 0.39 mm s^{-1}, and relative area = 47%.

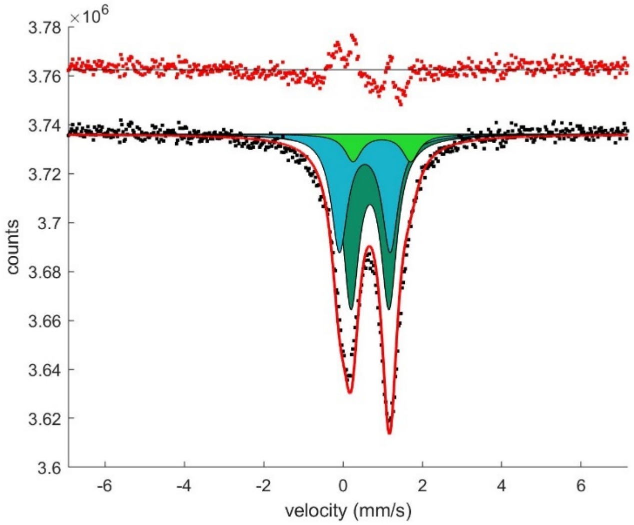

Figure S21. Solid state zero-field Mössbauer spectrum of LFeN(Me$_2$Si(CH$_2$)$_2$SiMe$_2$)NMe (**10**) where the black circles are the data, the red line is a two-component simulation, and the red circles are the residual. Fit parameters: dark green doublet (η^1 (S = 2), 54%): δ = 0.68 mm s^{-1}, $|\Delta E_Q|$ = 0.96 mm s^{-1}; light blue doublet (η^1 (S = 1), 37%): δ = 0.55 mm s^{-1}, $|\Delta E_Q|$ = 1.28 mm s^{-1}; light green doublet (η^2 (S = 2), 9%): δ = 0.98 mm s^{-1}, $|\Delta E_Q|$ = 1.45 mm s^{-1}. See Table S12 caption below for details regarding component assignment and fitting constraints.

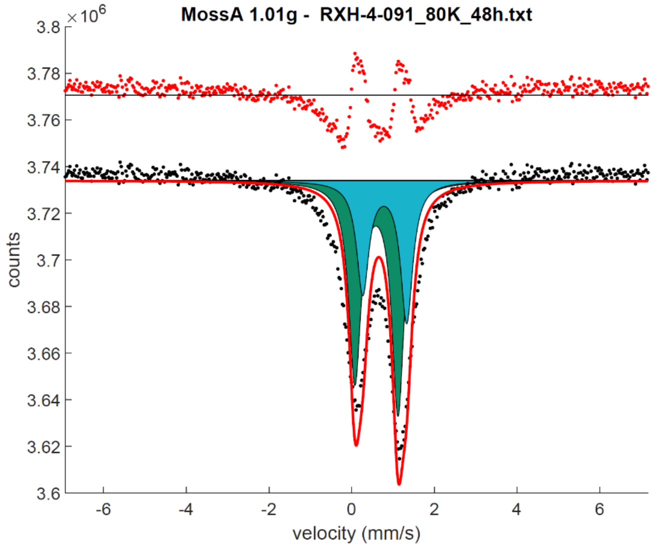

Figure S22: The same experimental data as S21 for **10** but with an alternative two-component fit. Fit parameters: light blue doublet (η^1 (S = 2), 63%): δ = 0.60 mm s^{-1}, $|\Delta E_Q|$ = 1.04 mm s^{-1}; dark green doublet (η^2 (S = 2), 37%): δ = 0.80 mm s^{-1}, $|\Delta E_Q|$ = 1.06 mm s^{-1}.

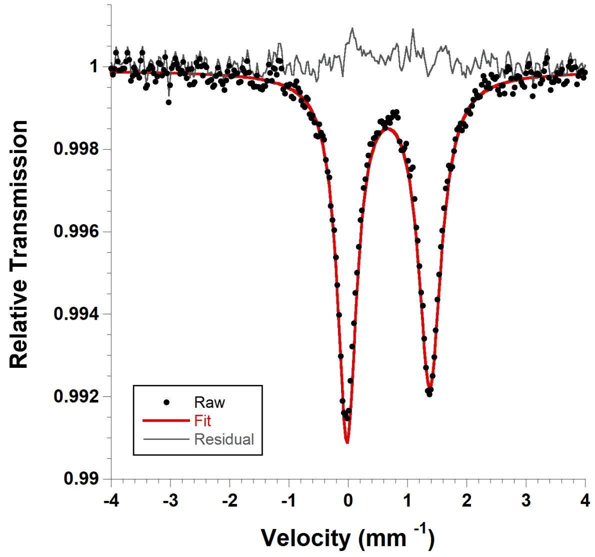

Figure S23. Zero-field Mössbauer spectrum of LFeN(Ph)N($Me_2Si(CH_2)_2SiMe_2$) (**12**) where the black circles are the data, the red line is a one-component simulation with parameters $\delta = 0.68$ mm s^{-1}, $|\Delta E_Q| = 1.40$ mm s^{-1}, $\Gamma_L = 0.40$ mm s^{-1}, $\Gamma_R = 0.46$ mm s^{-1}, and the gray line is the residual.

IR Spectroscopy

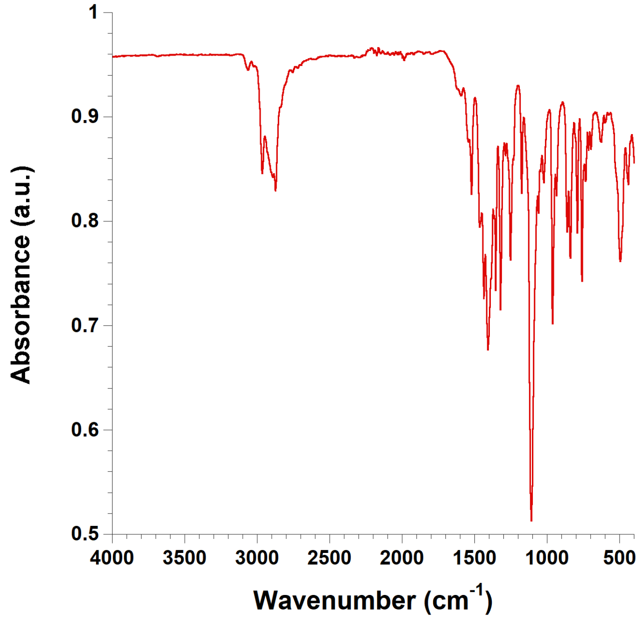

Figure S24. IR spectrum of solid [K(18-crown-6)(THF)$_2$][LFeCH$_2$SiMe$_3$] (**5a**).

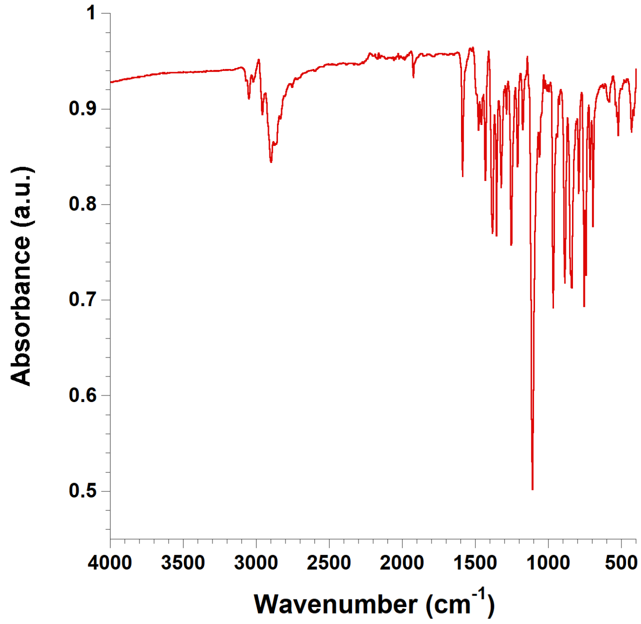

Figure S25. IR spectrum of solid [K(18-crown-6)(THF)$_2$][LFeBn] (**5b**).

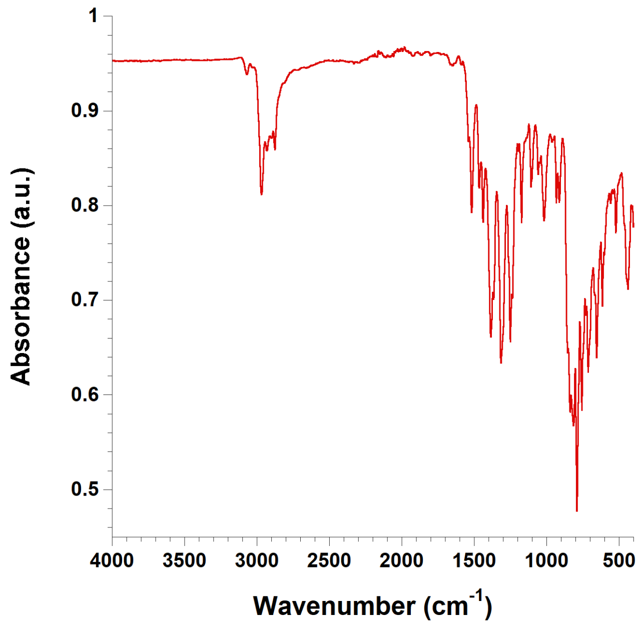

Figure S26. IR spectrum of solid LFe(CH_2SiMe_3)NN($Me_2Si(CH_2)_2SiMe_2$) (**7a**).

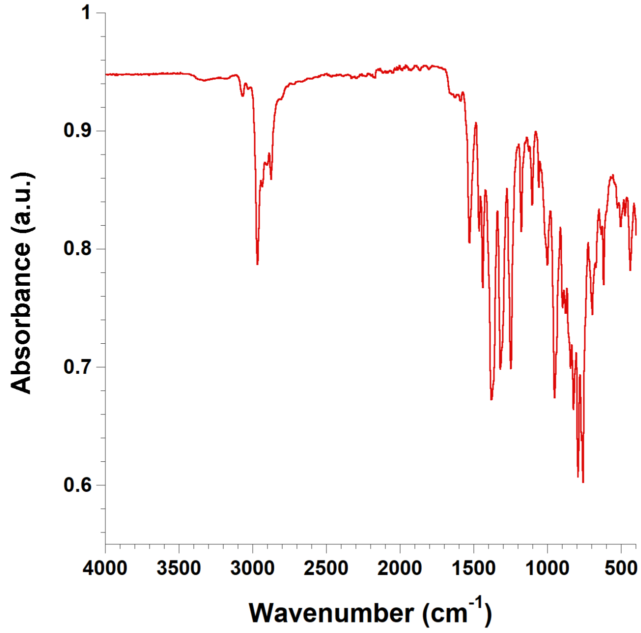

Figure S27. IR spectrum of solid LFeNN($Me_2Si(CH_2)_2SiMe_2$) (**8**).

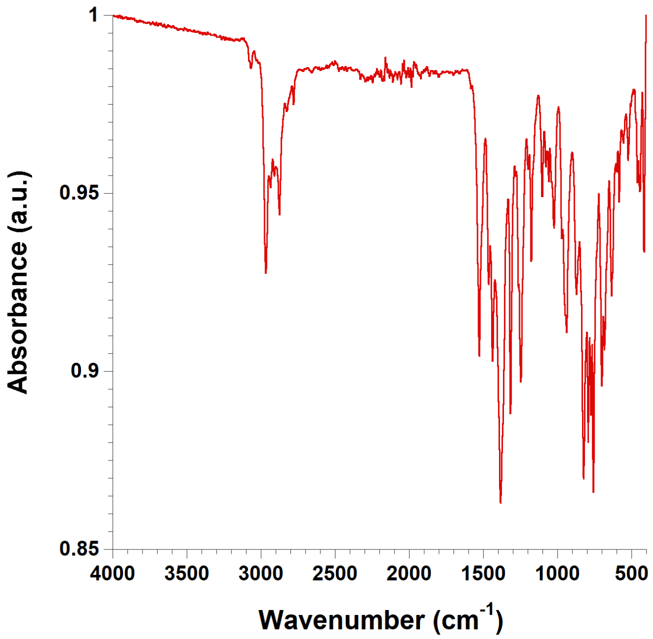

Figure S28. IR spectrum of solid LFeN($Me_2Si(CH_2)_2SiMe_2$)NMe (**10**).

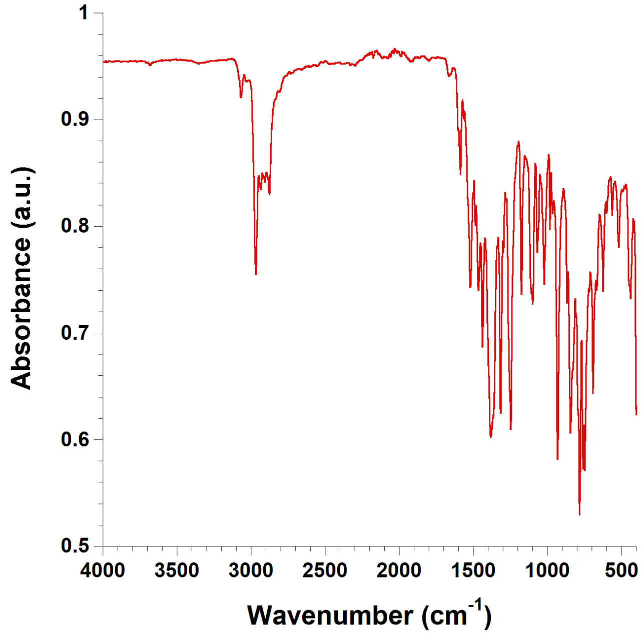

Figure S29. IR spectrum of solid LFeN(Ph)N($Me_2Si(CH_2)_2SiMe_2$) (**12**).

UV-Visible Spectroscopy

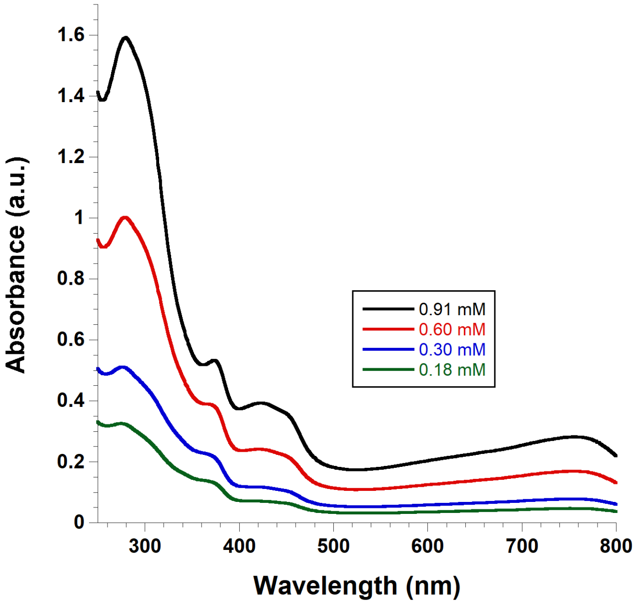

Figure S30. UV-visible spectra of [K(18-crown-6)(THF)$_2$][LFeCH$_2$SiMe$_3$] (**5a**) in THF solution with concentrations ranging from 0.91 to 0.18 mM.

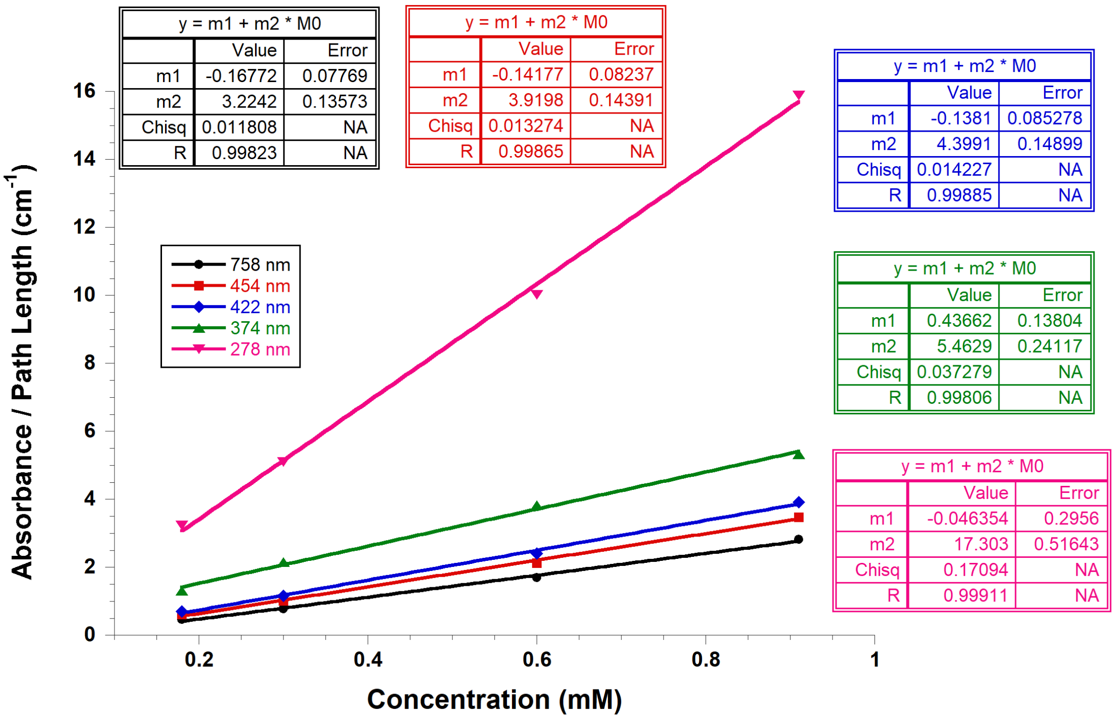

Figure S31. Beer–Lambert Law plot of selected UV-vis spectral features for [K(18-crown-6)(THF)$_2$][LFeCH$_2$SiMe$_3$] (**5a**) in THF solutions (0.91-0.18 mM). Trendline equations are in the boxes around the plot.

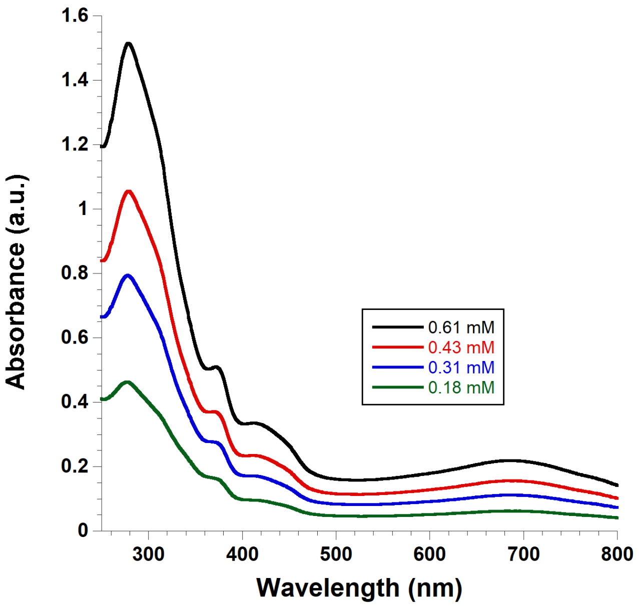

Figure S32. UV-visible spectra of [K(18-crown-6)(THF)$_2$][LFeBn] (**5b**) in THF solution with concentrations ranging from 0.61 to 0.18 mM.

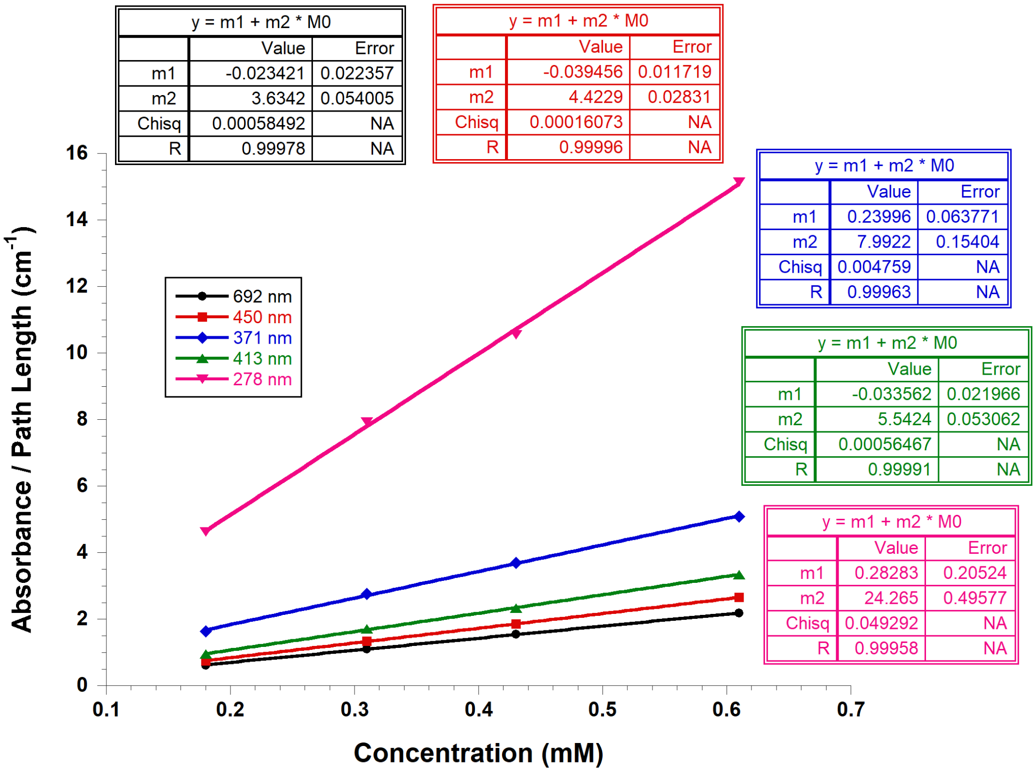

Figure S33. Beer–Lambert Law plot of selected UV-vis spectral features for [K(18-crown-6)(THF)$_2$][LFeBn] (**5b**) in THF solutions (0.61-0.18 mM). Trendline equations are in the boxes around the plot.

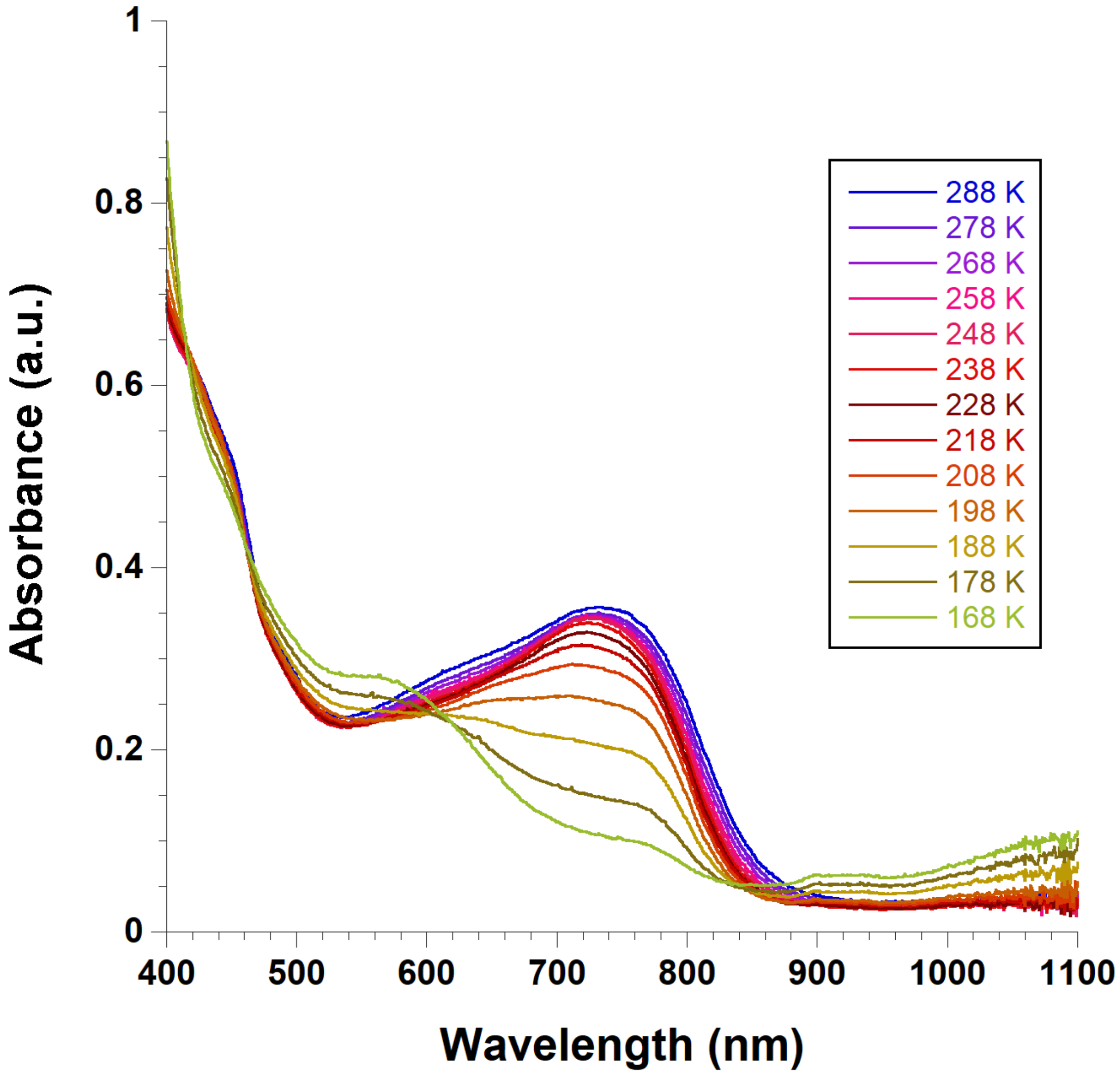

Figure S34. Variable-temperature UV-vis spectrum of [K(18-crown-6)(tol)][LFeMe] (**5c**) in THF under an atmosphere of N_2. Concentration = 0.192 mM.

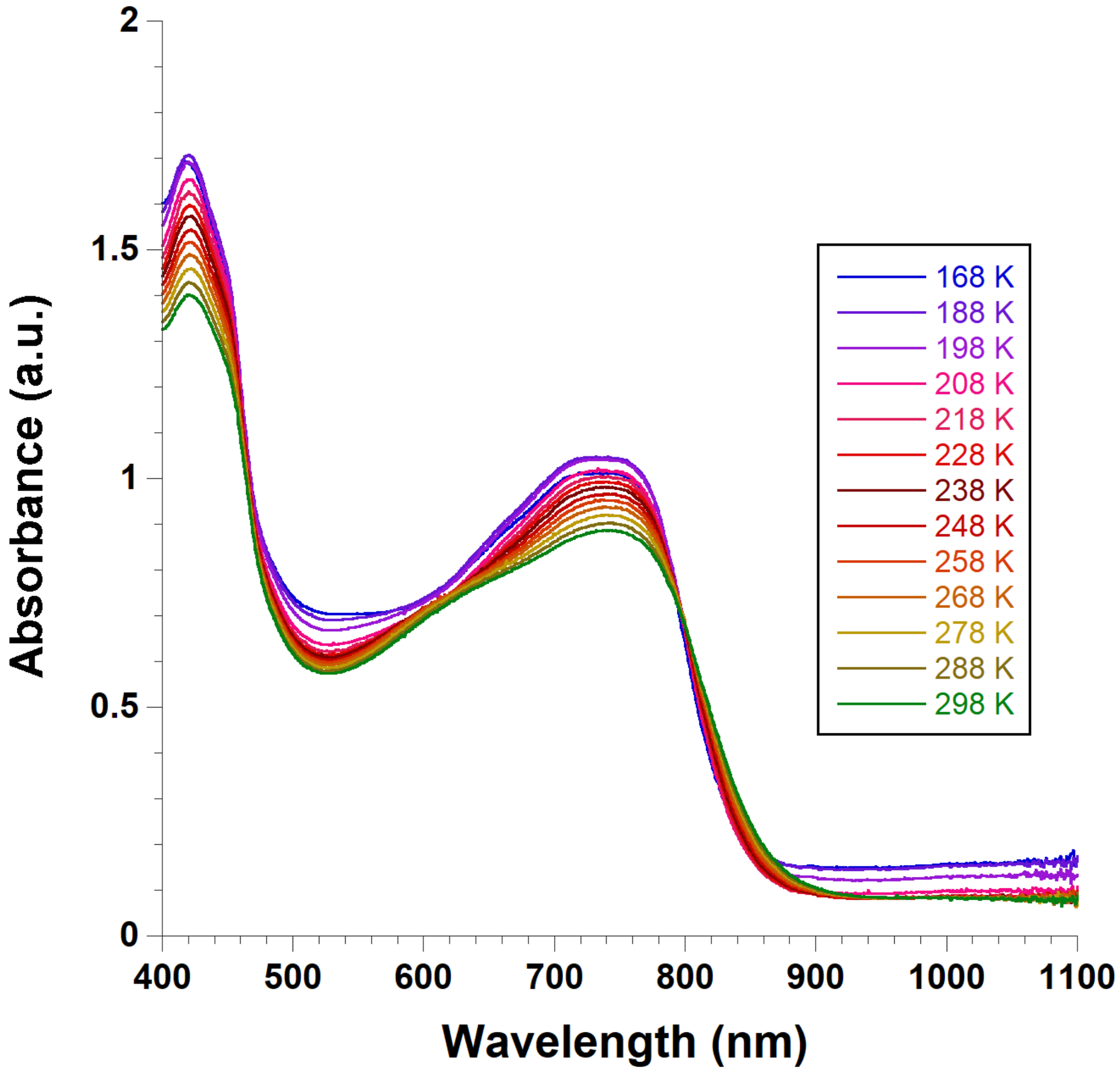

Figure S35. Variable-temperature UV-vis spectrum of [K(18-crown-6)(tol)][LFeMe] (**5c**) in THF under an atmosphere of Ar. Concentration = 0.351 mM.

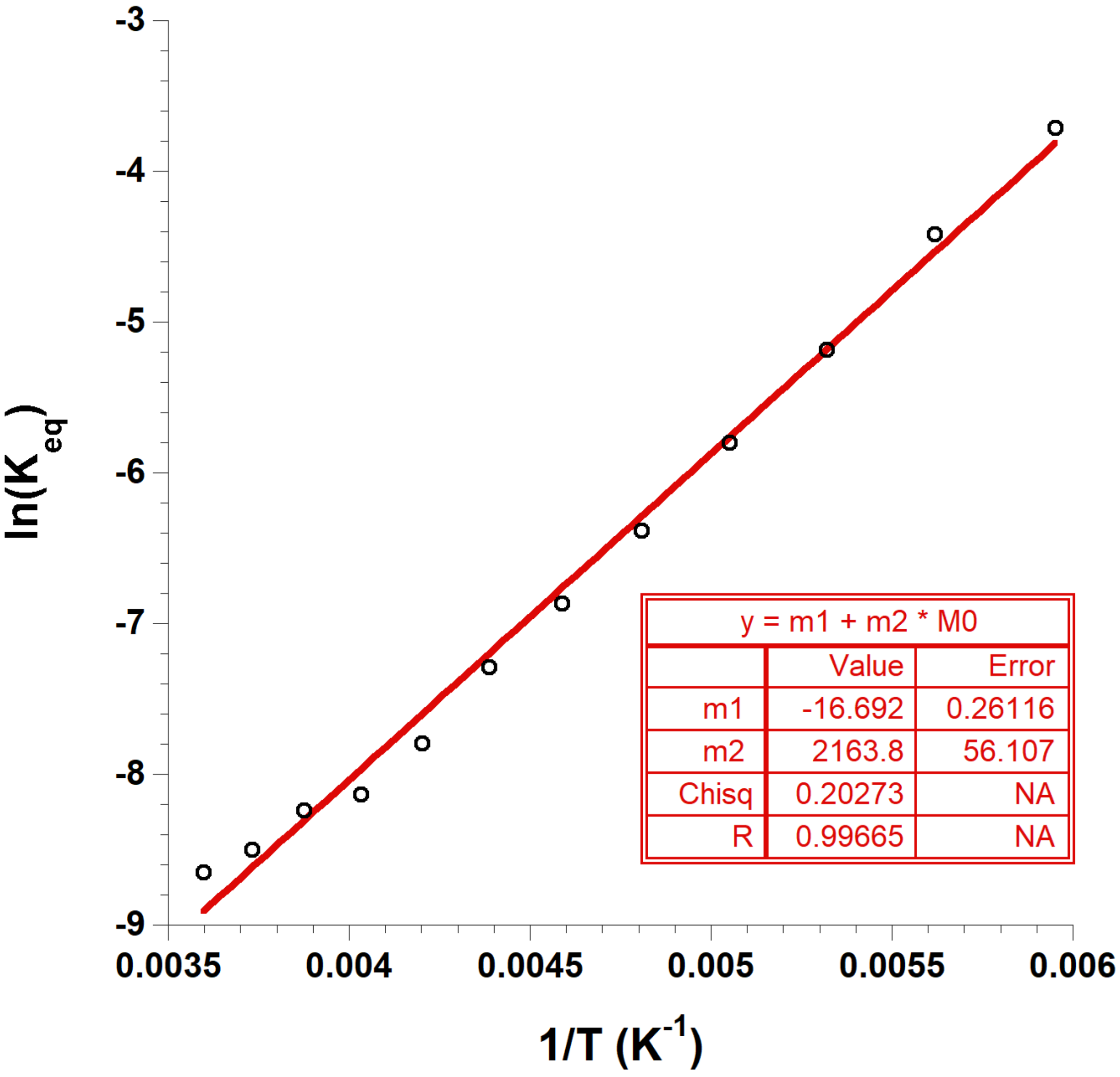

Figure S36. Van't Hoff plot for N_2 binding to [K(18-crown-6)(tol)][LFeMe] (**5c**) to form [K(18-crown-6)][LFe(N_2)Me] (**5c-N$_2$**) with points fit to a linear function. The solution concentration of N_2 was calculated using a literature equation,[11] additionally accounting for the pressure changes with temperature in the closed system. Equilibrium constants were calculated from the absorbance change at 731 nm in the UV-vis spectra in Fig. S28. The concentration of **5c** was assumed to account for the majority of the absorbance at this wavelength at 288 K and the decrease in absorbance was attributed to formation of **5c-N$_2$**. Conversion to **5c-N$_2$** was incomplete even at 168 K, so it was necessary to estimate its absorbance at 731 nm as 0.05. The wide uncertainty values given in the text are chosen to generously account for this potential source of error.

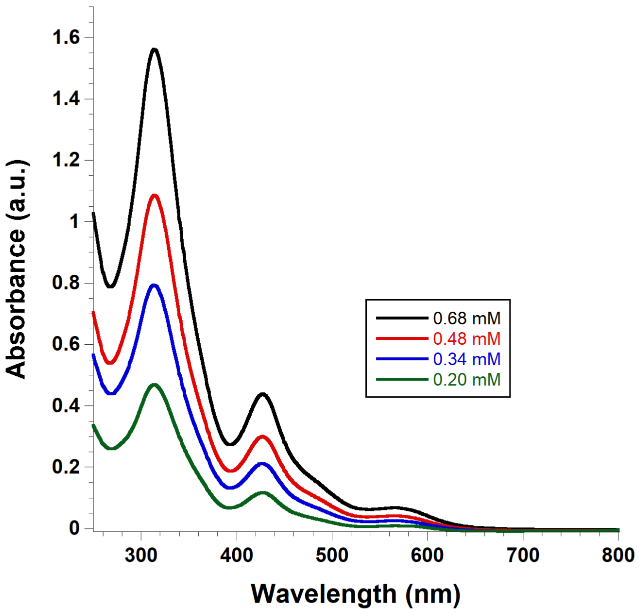

Figure S37. UV-visible spectra of $LFe(CH_2SiMe_3)NN(Me_2Si(CH_2)_2SiMe_2)$ (**7a**) in hexanes solution with concentrations ranging from 0.68 to 0.20 mM.

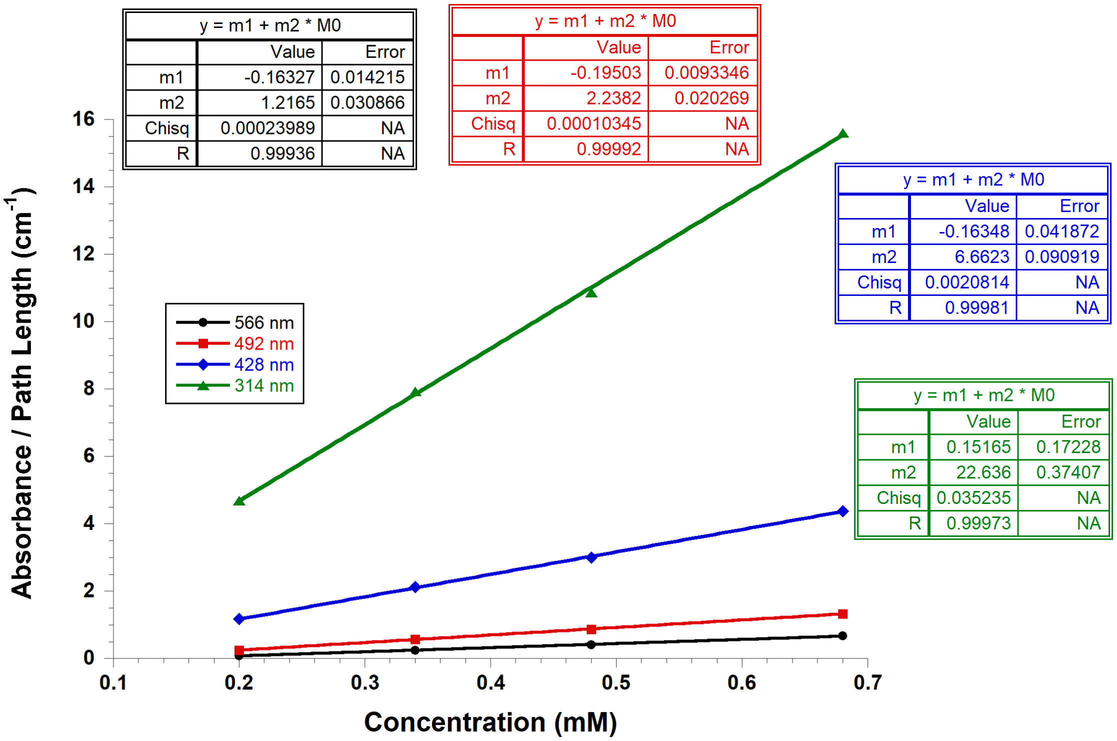

Figure S38. Beer–Lambert Law plot of selected UV-vis spectral features for $LFe(CH_2SiMe_3)NN(Me_2Si(CH_2)_2SiMe_2)$ (**7a**) in hexanes solutions (0.68-0.20 mM). Trendline equations are in the boxes around the plot.

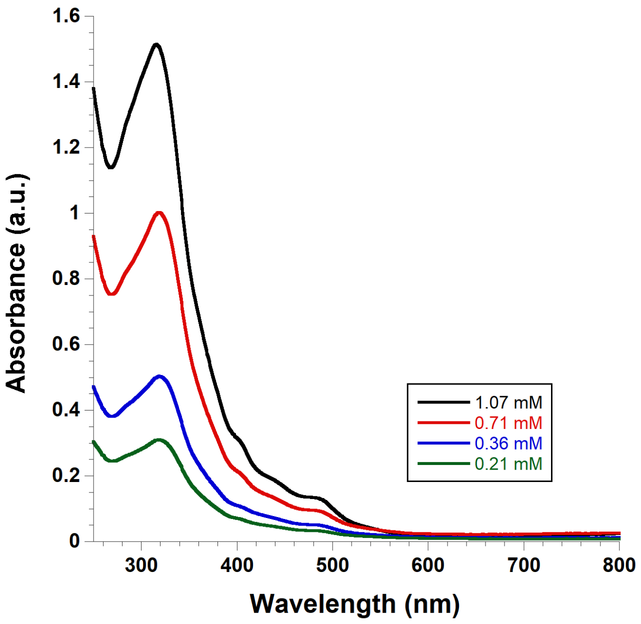

Figure S39. UV-visible spectra of LFeNN($Me_2Si(CH_2)_2SiMe_2$) (**8**) in hexanes solution with concentrations ranging from 1.07 to 0.21 mM.

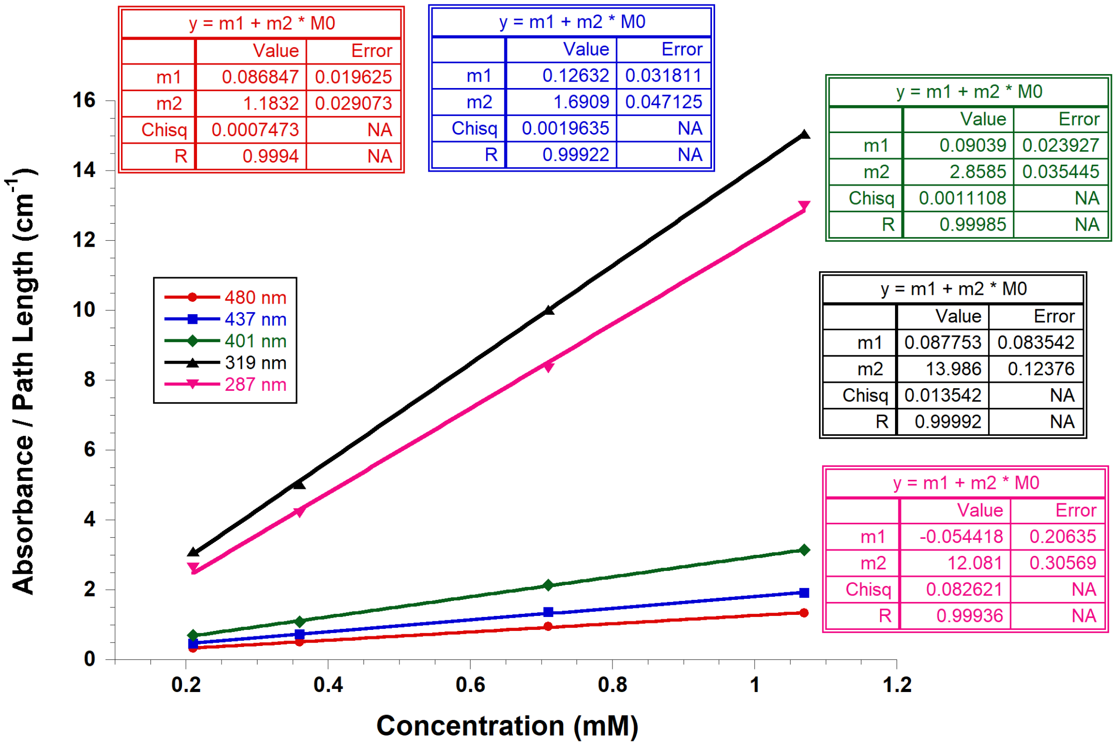

Figure S40. Beer–Lambert Law plot of selected UV-vis spectral features for LFeNN($Me_2Si(CH_2)_2SiMe_2$) (**8**) in hexanes solutions (1.07-0.21 mM). Trendline equations are in the boxes around the plot.

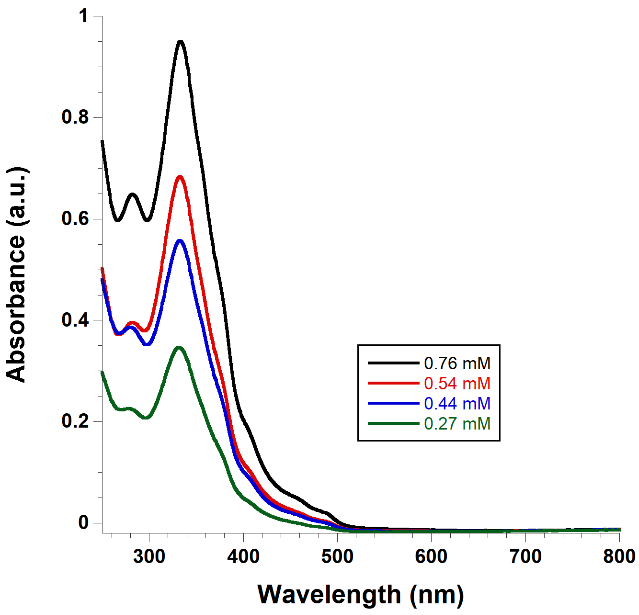

Figure S50. UV-visible spectra of LFeN($Me_2Si(CH_2)_2SiMe_2$)Me (**10**) in hexanes solution with concentrations ranging from 0.76 to 0.27 mM.

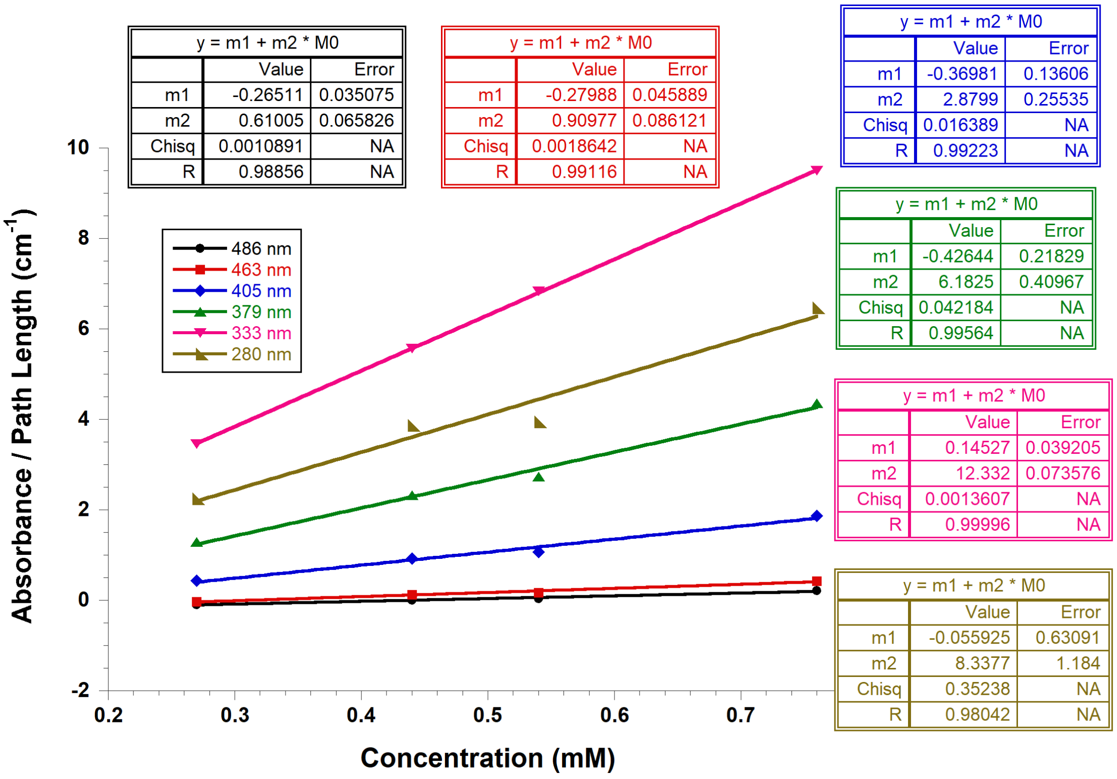

Figure S51. Beer–Lambert Law plot of selected UV-vis spectral features for LFeN($Me_2Si(CH_2)_2SiMe_2$)Me (**10**) in hexanes solutions (0.76-0.27 mM). Trendline equations are in the boxes around the plot.

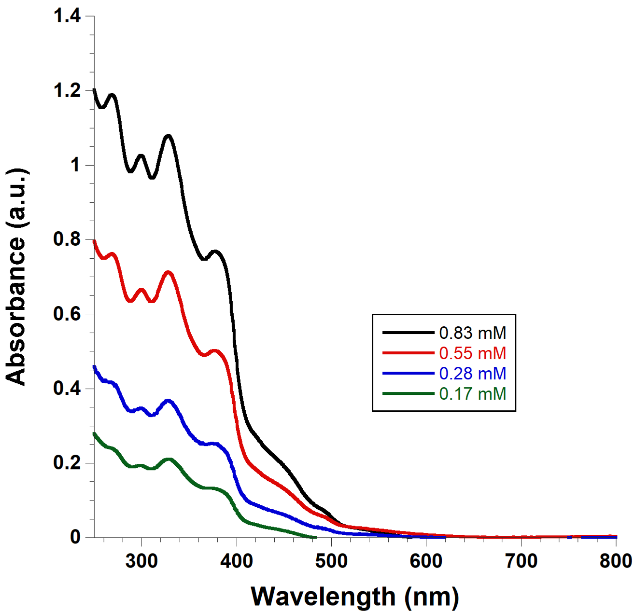

Figure S52. UV-visible spectra of LFeN(Ph)N($Me_2Si(CH_2)_2SiMe_2$) (**12**) in hexanes solution with concentrations ranging from 0.83 to 0.17 mM.

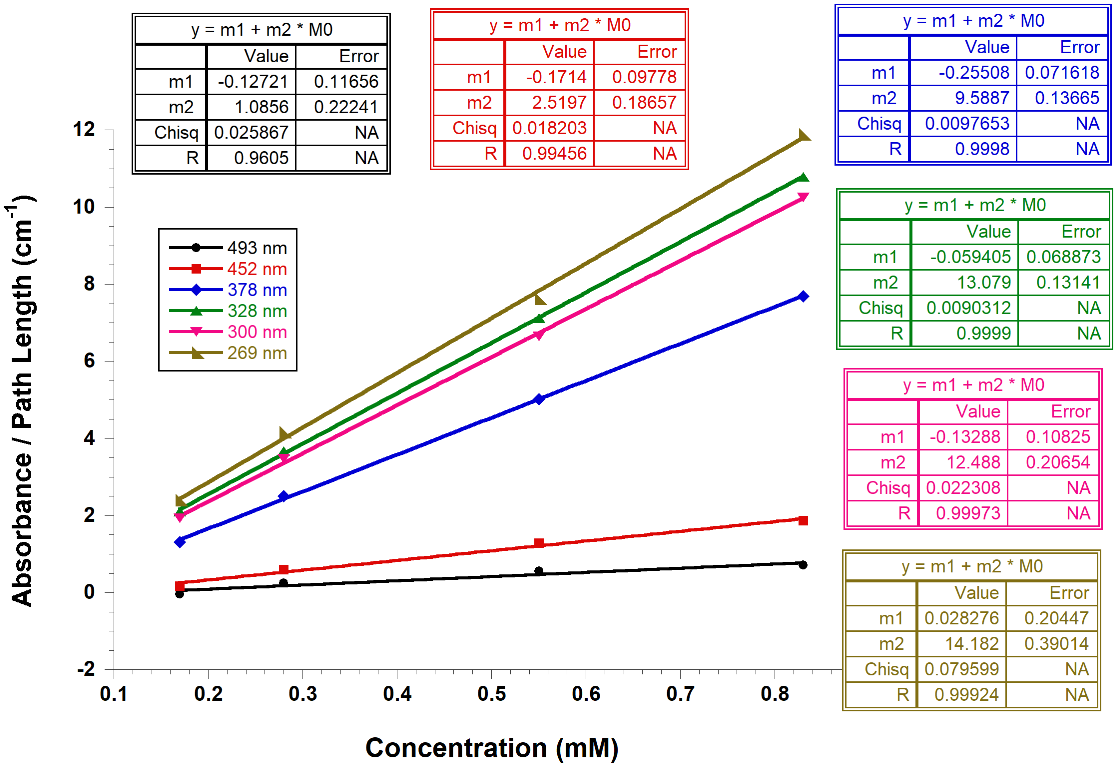

Figure S53. Beer–Lambert Law plot of selected UV-vis spectral features for LFeN(Ph)N($Me_2Si(CH_2)_2SiMe_2$) (**12**) in hexanes solutions (0.83-0.17 mM). Trendline equations are in the boxes around the plot.

Crystallography

[K(18-crown-6)$(THF)_2$][$LFeCH_2SiMe_3$] (5a)

Low-temperature diffraction data (ω-scans) were collected on a Rigaku SCX Mini diffractometer coupled to a Rigaku Mercury275R CCD with Mo Kα radiation (λ = 0.71073 Å) for the structure of **5a**. The diffraction images were processed and scaled using Rigaku Oxford Diffraction software (CrysAlisPro; Rigaku OD: The Woodlands, TX, 2015). The structure was solved with SHELXT and was refined against F^2 on all data by full-matrix least squares with SHELXL.[12] All non-hydrogen atoms were refined anisotropically. Hydrogen atoms were included in the model at geometrically calculated positions and refined using a riding model. The isotropic displacement parameters of all hydrogen atoms were fixed to 1.2 times the U value of the atoms to which they are linked (1.5 times for methyl groups). The full numbering scheme of compound **5a** can be found in the full details of the X-ray structure determination (CIF), which is included as Supporting Information. CCDC number 2226338 contains the supplementary crystallographic data for this paper. These data can be obtained free of charge from The Cambridge Crystallographic Data Center via www.ccdc.cam.ac.uk/data_request/cif.

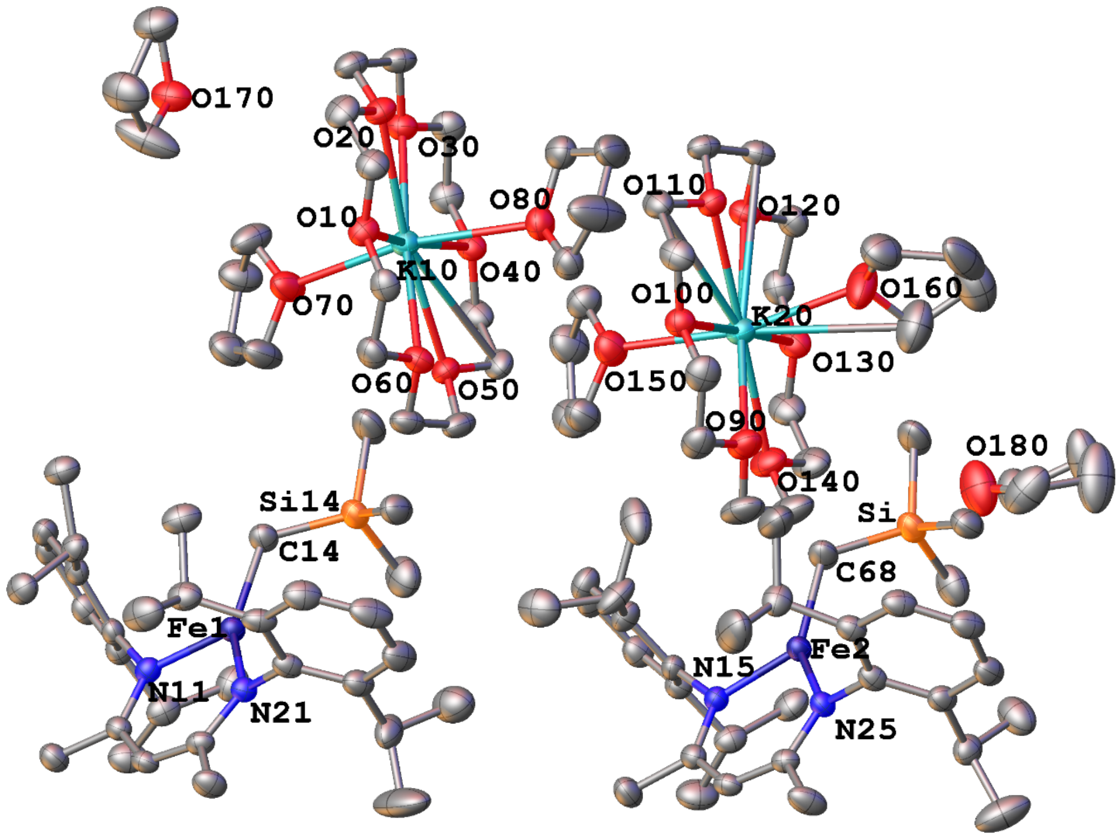

Figure S54. A partial numbering scheme of **5a** with 50% thermal ellipsoid probability levels. The hydrogen atoms are omitted for clarity.

Table S1. Crystal data and structure refinement for **5a**.

Identification code	mini-21065	
CCDC Number	2226338	
Empirical formula	C57 H100 Fe K N2 O9 Si	
Formula weight	1080.42	
Temperature	93(2) K	
Wavelength	0.71073 Å	
Crystal system	Monoclinic	
Space group	$P2_1/n$	
Unit cell dimensions	a = 24.2280(7) Å	α= 90°.
	b = 19.8535(4) Å	β= 114.199(4)°.
	c = 27.1355(9) Å	γ = 90°.
Volume	11905.5(7) $Å^3$	
Z	8	
Density (calculated)	1.206 g/cm^3	
Absorption coefficient	0.396 mm^{-1}	
F(000)	4696	
Crystal size	0.200 x 0.200 x 0.100 mm^3	
Crystal color and habit	Black Plate	
Diffractometer	Rigaku Mercury275R CCD	
Theta range for data collection	2.740 to 26.372°.	
Index ranges	-30<=h<=30, -24<=k<=24, -33<=l<=33	
Reflections collected	189898	
Independent reflections	24308 [R(int) = 0.0713]	
Observed reflections (I > 2sigma(I))	20016	
Completeness to theta = 25.242°	99.9 %	
Absorption correction	Semi-empirical from equivalents	
Max. and min. transmission	1.00000 and 0.84720	
Solution method	SHELXT-2014/5 (Sheldrick, 2014)	
Refinement method	SHELXL-2014/7 (Sheldrick, 2014)	
Data / restraints / parameters	24308 / 0 / 1305	
Goodness-of-fit on F^2	1.107	
Final R indices [I>2sigma(I)]	R1 = 0.0725, wR2 = 0.1680	
R indices (all data)	R1 = 0.0889, wR2 = 0.1809	
Largest diff. peak and hole	0.967 and -0.597 e.$Å^{-3}$	

[K(18-crown-6)(THF)$_2$][LFeBn] (5b)

Low-temperature diffraction data (ω-scans) were collected on a Rigaku MicroMax-007HF diffractometer coupled to a Saturn994+ CCD detector with Cu Kα (λ = 1.54178 Å). The diffraction images were processed and scaled using Rigaku Oxford Diffraction software (CrysAlisPro; Rigaku OD: The Woodlands, TX, 2015). The structure was solved with SHELXT and was refined against F^2 on all data by full-matrix least squares with SHELXL.[12] All non-hydrogen atoms were refined anisotropically. Hydrogen atoms were included in the model at geometrically calculated positions and refined using a riding model. The isotropic displacement parameters of all hydrogen atoms were fixed to 1.2 times the U value of the atoms to which they are linked (1.5 times for methyl groups). CCDC number 2226339 contains the supplementary crystallographic data for this paper.

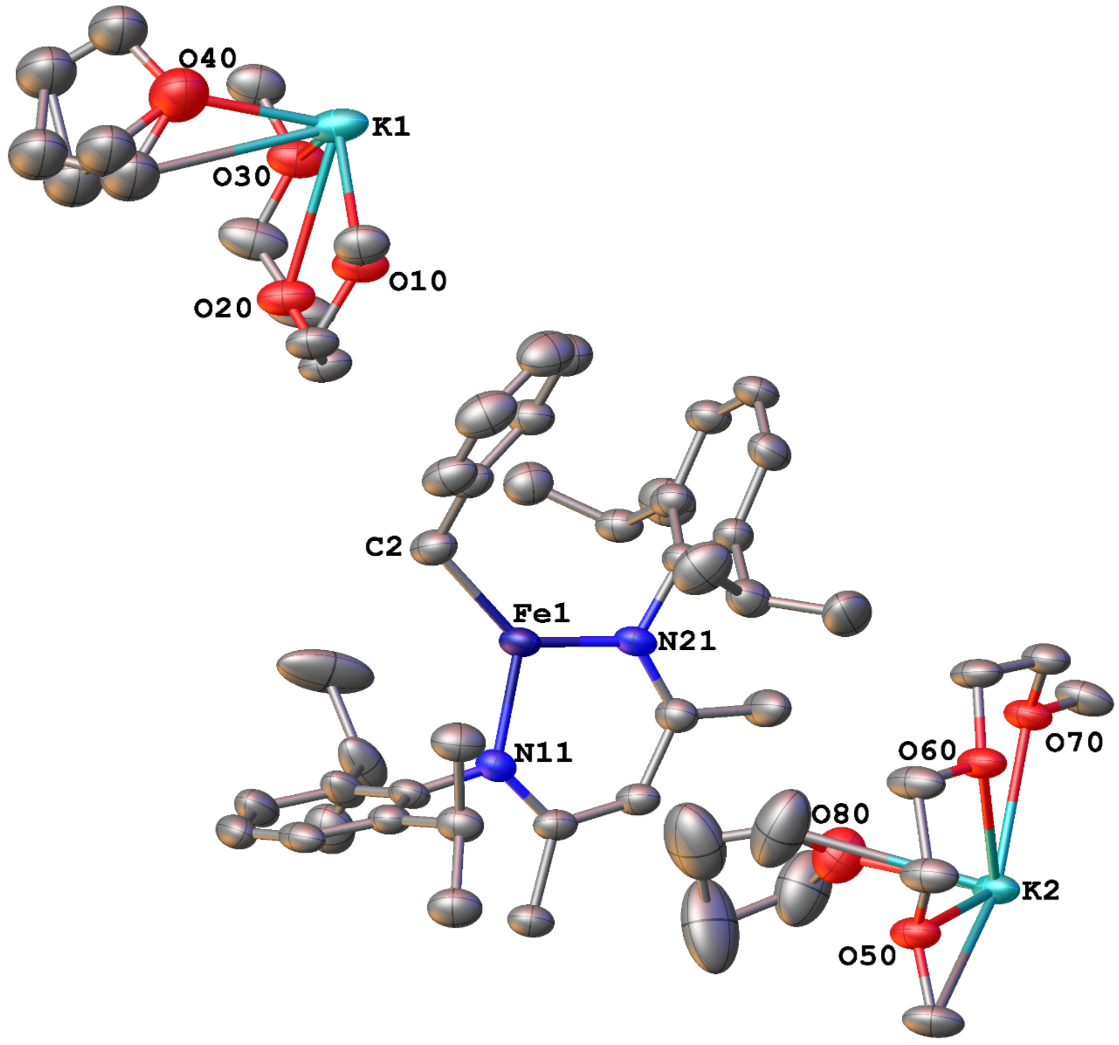

Figure S55. A partial numbering scheme of **5b** with 50% thermal ellipsoid probability levels. The hydrogen atoms are omitted for clarity. The potassium ions lie on crystallographic inversion centers.

Table S2. Crystal data and structure refinement for **5b**.

Identification code	007a-21112	
CCDC Number	2226339	
Empirical formula	C56 H88 Fe K N2 O8	
Formula weight	1012.23	
Temperature	93(2) K	
Wavelength	1.54184 Å	
Crystal system	Triclinic	
Space group	P-1	
Unit cell dimensions	a = 13.0503(6) Å	α= 83.732(3)°.
	b = 13.4395(6) Å	β= 71.934(4)°.
	c = 20.4096(7) Å	γ = 66.707(4)°.
Volume	3125.4(3) Å^3	
Z	2	
Density (calculated)	1.076 g/cm^3	
Absorption coefficient	2.897 mm^{-1}	
F(000)	1094	
Crystal size	0.200 x 0.200 x 0.100 mm^3	
Crystal color and habit	Black Plate	
Diffractometer	Rigaku Saturn 944+ CCD	
Theta range for data collection	2.277 to 66.559°.	
Index ranges	-15<=h<=15, -15<=k<=15, -24<=l<=24	
Reflections collected	112250	
Independent reflections	10888 [R(int) = 0.0757]	
Observed reflections (I > 2sigma(I))	8540	
Completeness to theta = 66.559°	98.6 %	
Absorption correction	Semi-empirical from equivalents	
Max. and min. transmission	1.00000 and 0.75467	
Solution method	SHELXT-2014/5 (Sheldrick, 2014)	
Refinement method	SHELXL-2014/7 (Sheldrick, 2014)	
Data / restraints / parameters	10888 / 75 / 645	
Goodness-of-fit on F^2	1.045	
Final R indices [I>2sigma(I)]	R1 = 0.0734, wR2 = 0.1986	
R indices (all data)	R1 = 0.0901, wR2 = 0.2118	
Largest diff. peak and hole	1.585 and -0.710 e.Å^{-3}	

LFe(CH_2SiMe_3)NN($Me_2Si(CH_2)_2SiMe_2$) (7a)

Low-temperature diffraction data (ω-scans) were collected on a Rigaku MicroMax-007HF diffractometer coupled to a Saturn994+ CCD detector with Cu Kα (λ = 1.54178 Å). The diffraction images were processed and scaled using Rigaku Oxford Diffraction software (CrysAlisPro; Rigaku OD: The Woodlands, TX, 2015). The structure was solved with SHELXT and was refined against F^2 on all data by full-matrix least squares with SHELXL.[12] All non-hydrogen atoms were refined anisotropically. Hydrogen atoms were included in the model at geometrically calculated positions and refined using a riding model. The isotropic displacement parameters of all hydrogen atoms were fixed to 1.2 times the U value of the atoms to which they are linked (1.5 times for methyl groups). The program SQUEEZE[13] was used to compensate for the contribution of disordered solvents contained in voids within the crystal lattice from the diffraction intensities. This procedure was applied to the data file and the submitted model is based on the solvent removed data. Based on the total electron density found in the voids (47 e/Å^3), it is likely that a hexane molecule is present in the unit cell. See "_platon_squeeze_details" in this .cif for more information. CCDC number 2226340 contains the supplementary crystallographic data for this paper.

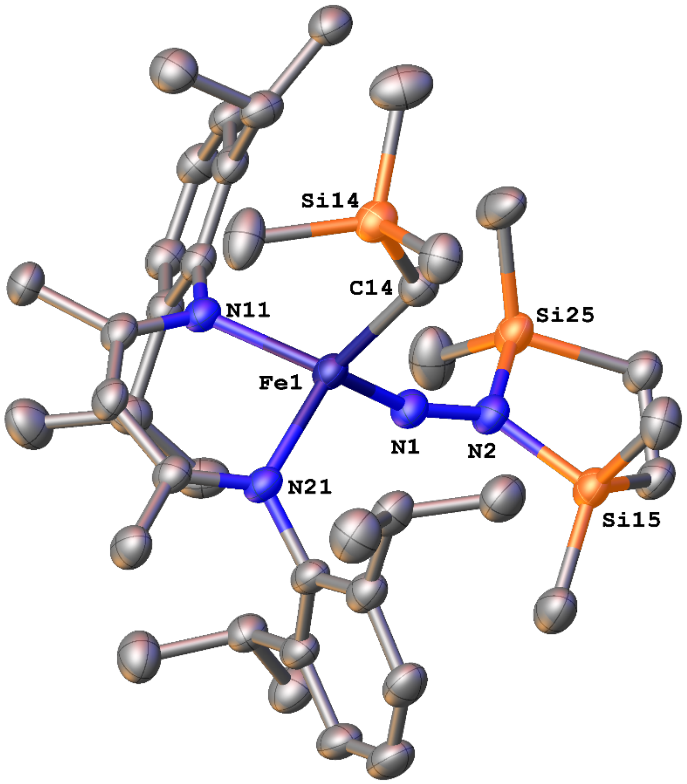

Figure S56. A partial numbering scheme of **7a** with 50% thermal ellipsoid probability levels. The hydrogen atoms are omitted for clarity.

Table S3. Crystal data and structure refinement for **7a**.

Identification code	007a-22088	
CCDC Number	2226340	
Empirical formula	C39 H68 Fe N4 Si3	
Formula weight	733.09	
Temperature	93(2) K	
Wavelength	1.54184 Å	
Crystal system	Triclinic	
Space group	P-1	
Unit cell dimensions	a = 10.3800(4) Å	α= 76.000(3)°.
	b = 11.5329(4) Å	β= 82.547(3)°.
	c = 20.2705(6) Å	γ = 72.992(3)°.
Volume	2247.01(14) $Å^3$	
Z	2	
Density (calculated)	1.084 g/cm^3	
Absorption coefficient	3.663 mm^{-1}	
F(000)	796	
Crystal size	0.200 x 0.200 x 0.100 mm^3	
Crystal color and habit	Black Plate	
Diffractometer	Rigaku Saturn 944+ CCD	
Theta range for data collection	2.251 to 66.600°.	
Index ranges	-12<=h<=12, -13<=k<=13, -24<=l<=23	
Reflections collected	66616	
Independent reflections	7797 [R(int) = 0.0490]	
Observed reflections (I > 2sigma(I))	7241	
Completeness to theta = 66.600°	98.0 %	
Absorption correction	Semi-empirical from equivalents	
Max. and min. transmission	1.00000 and 0.61604	
Solution method	SHELXT-2014/5 (Sheldrick, 2014)	
Refinement method	SHELXL-2014/7 (Sheldrick, 2014)	
Data / restraints / parameters	7797 / 0 / 441	
Goodness-of-fit on F^2	1.051	
Final R indices [I>2sigma(I)]	R1 = 0.0455, wR2 = 0.1182	
R indices (all data)	R1 = 0.0488, wR2 = 0.1211	
Largest diff. peak and hole	0.542 and -0.586 e.$Å^{-3}$	

LFeN(Me$_2$Si(CH$_2$)$_2$SiMe$_2$)

Low-temperature diffraction data (ω-scans) were collected on a Rigaku MicroMax-007HF diffractometer coupled to a Saturn994+ CCD detector with Cu Kα (λ = 1.54178 Å). The diffraction images were processed and scaled using Rigaku Oxford Diffraction software (CrysAlisPro; Rigaku OD: The Woodlands, TX, 2015). The structure was solved with SHELXT and was refined against F^2 on all data by full-matrix least squares with SHELXL.[12] All non-hydrogen atoms were refined anisotropically. Hydrogen atoms were included in the model at geometrically calculated positions and refined using a riding model. The isotropic displacement parameters of all hydrogen atoms were fixed to 1.2 times the *U* value of the atoms to which they are linked (1.5 times for methyl groups). Carbon 4 was disordered over two equally occupied positions. No additional restraints or constraints were needed for the disordered model to converge. CCDC number 2226341 contains the supplementary crystallographic data for this structure.

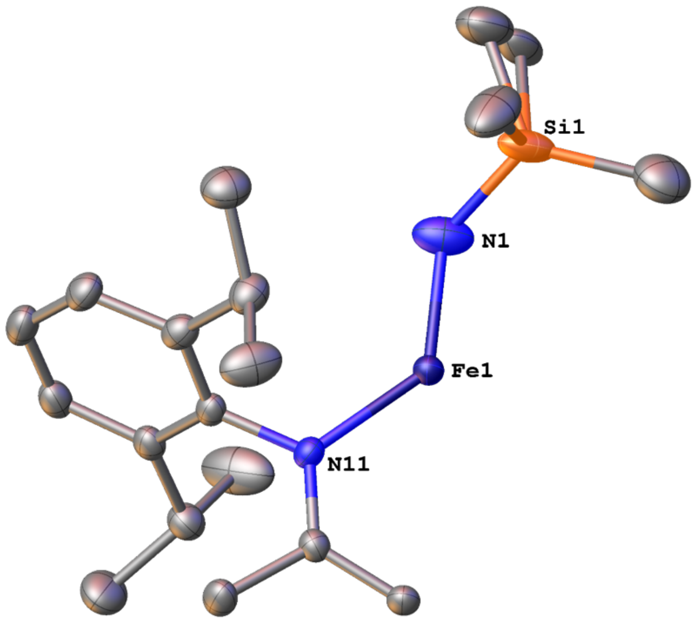

Figure S57. A partial numbering scheme of **LFeN(Me$_2$Si(CH$_2$)$_2$SiMe$_2$)** with 50% thermal ellipsoid probability levels. The hydrogen atoms are omitted for clarity. Only the asymmetric unit is shown; the molecule lies on a crystallographic 2-fold rotation axis.

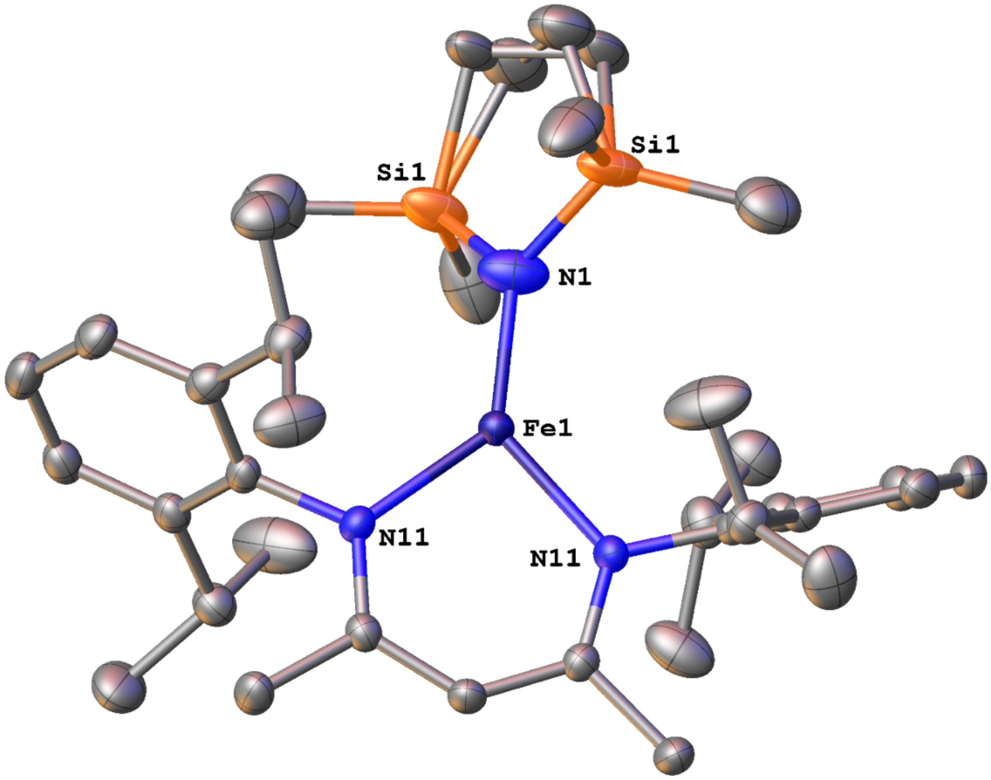

Figure S58. A partial numbering scheme showing the full model of **LFeN(Me$_2$Si(CH$_2$)$_2$SiMe$_2$)** with 50% thermal ellipsoid probability levels. The hydrogen atoms are omitted for clarity. In this diagram, the symmetry-related atoms (two-fold rotation axis) are shown with the same label.

Table S4. Crystal data and structure refinement for **LFeN($Me_2Si(CH_2)_2SiMe_2$)**.

Identification code	007a-22067	
CCDC Number	2226341	
Empirical formula	C35 H57 Fe N3 Si2	
Formula weight	631.86	
Temperature	93(2) K	
Wavelength	1.54184 Å	
Crystal system	Orthorhombic	
Space group	Pbcn	
Unit cell dimensions	a = 10.11510(10) Å	α= 90°.
	b = 17.6923(2) Å	β= 90°.
	c = 20.5843(3) Å	γ = 90°.
Volume	3683.75(8) $Å^3$	
Z	4	
Density (calculated)	1.139 g/cm^3	
Absorption coefficient	4.087 mm^{-1}	
F(000)	1368	
Crystal size	0.200 x 0.200 x 0.020 mm^3	
Crystal color and habit	Black Plate	
Diffractometer	Rigaku Saturn 944+ CCD	
Theta range for data collection	5.000 to 66.583°.	
Index ranges	-12<=h<=12, -21<=k<=21, -24<=l<=24	
Reflections collected	107553	
Independent reflections	3248 [R(int) = 0.1035]	
Observed reflections (I > 2sigma(I))	2998	
Completeness to theta = 66.583°	99.7 %	
Absorption correction	Semi-empirical from equivalents	
Max. and min. transmission	1.00000 and 0.66668	
Solution method	SHELXT-2014/5 (Sheldrick, 2014)	
Refinement method	SHELXL-2014/7 (Sheldrick, 2014)	
Data / restraints / parameters	3248 / 6 / 203	
Goodness-of-fit on F^2	1.100	
Final R indices [I>2sigma(I)]	R1 = 0.0488, wR2 = 0.1297	
R indices (all data)	R1 = 0.0521, wR2 = 0.1324	
Largest diff. peak and hole	0.982 and -0.735 e.$Å^{-3}$	

LFeNN($Me_2Si(CH_2)_2SiMe_2$) (8)

Low-temperature diffraction data (ω-scans) were collected on a Rigaku MicroMax-007HF diffractometer coupled to a Saturn994+ CCD detector with Cu Kα (λ = 1.54178 Å). The diffraction images were processed and scaled using Rigaku Oxford Diffraction software (CrysAlisPro; Rigaku OD: The Woodlands, TX, 2015). The structure was solved with SHELXT and was refined against F^2 on all data by full-matrix least squares with SHELXL.[12] All non-hydrogen atoms were refined anisotropically. Hydrogen atoms were included in the model at geometrically calculated positions and refined using a riding model. The isotropic displacement parameters of all hydrogen atoms were fixed to 1.2 times the U value of the atoms to which they are linked (1.5 times for methyl groups). The reflections were refined as a 2-component twin. The disilylhydrazido group is present in partial occupancy. The site occupancy factors (SOFs) for atoms Si1A, Si2A, N1A, N2A, C30A, C31A were freely refined and converged near a value of 0.75. These SOFs were subsequently fixed at 0.75. The minor component was modeled with SOFs of 0.25, and the chemical composition of this second species is not yet known. CCDC number 2226342 contains the supplementary crystallographic data for this structure.

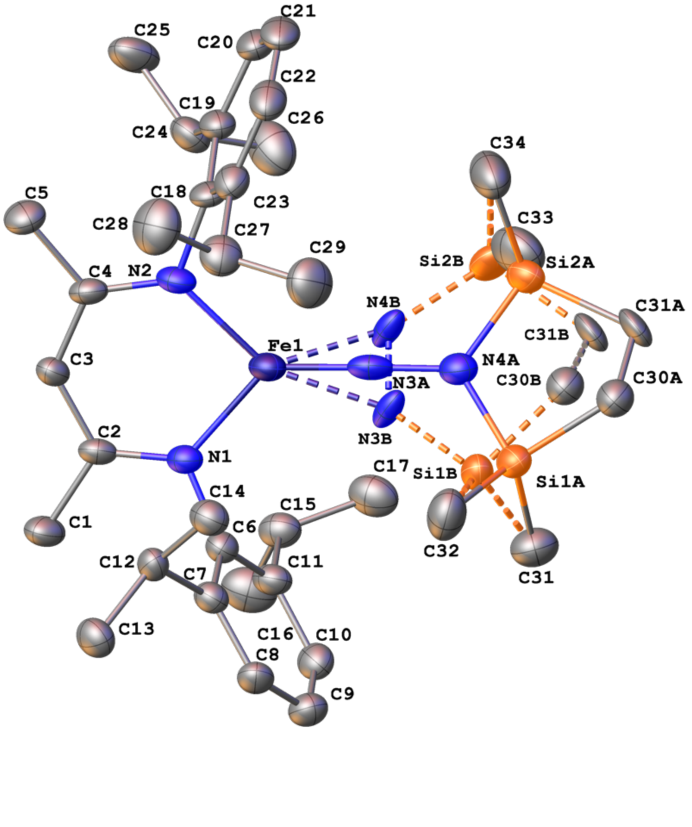

Figure S59. A complete numbering scheme of the crystallographic model of **8** with 50% thermal ellipsoid probability levels. The major component (0.75 occupancy) is atoms labeled as A, and the minor component (0.25 occupance) is atoms labeled as B. The identity of the minor component is not known. The hydrogen atoms are omitted for clarity.

Table S5. Crystal data and structure refinement for **8**.

Identification code	007a-22141	
CCDC Number	2226342	
Empirical formula	C35 H57 Fe N4 Si2	
Formula weight	645.87	
Temperature	93(2) K	
Wavelength	1.54184 Å	
Crystal system	Monoclinic	
Space group	$P2_1/n$	
Unit cell dimensions	a = 10.6104(5) Å	α= 90°.
	b = 20.3504(8) Å	β= 95.909(4)°.
	c = 17.3268(8) Å	γ = 90°.
Volume	3721.4(3) $Å^3$	
Z	4	
Density (calculated)	1.153 g/cm^3	
Absorption coefficient	4.065 mm^{-1}	
F(000)	1396	
Crystal size	0.200 x 0.200 x 0.020 mm^3	
Crystal color and habit	Black Plate	
Diffractometer	Rigaku Saturn 944+ CCD	
Theta range for data collection	3.360 to 66.652°.	
Index ranges	-12<=h<=12, -24<=k<=24, -16<=l<=20	
Reflections collected	6549	
Independent reflections	6549 [R(int) = 0.1175]	
Observed reflections (I > 2sigma(I))	4992	
Completeness to theta = 66.652°	99.5 %	
Absorption correction	Semi-empirical from equivalents	
Max. and min. transmission	1.00000 and 0.84505	
Solution method	SHELXT-2014/5 (Sheldrick, 2014)	
Refinement method	SHELXL-2014/7 (Sheldrick, 2014)	
Data / restraints / parameters	6549 / 6 / 436	
Goodness-of-fit on F^2	1.087	
Final R indices [I>2sigma(I)]	R1 = 0.0819, wR2 = 0.2318	
R indices (all data)	R1 = 0.1047, wR2 = 0.2464	
Largest diff. peak and hole	0.843 and -0.675 e.$Å^{-3}$	

LFeN(Me$_2$Si(CH$_2$)$_2$SiMe$_2$)NMe (10)

Low-temperature diffraction data (ω-scans) were collected on a Rigaku MicroMax-007HF diffractometer coupled to a Saturn994+ CCD detector with Cu Kα (λ = 1.54178 Å). The diffraction images were processed and scaled using Rigaku Oxford Diffraction software. The structure was solved with SHELXT and was refined against F^2 on all data by full-matrix least squares with SHELXL.[12] All non-hydrogen atoms were refined anisotropically. Hydrogen atoms were included in the model at geometrically calculated positions and refined using a riding model. The isotropic displacement parameters of all hydrogen atoms were fixed to 1.2 times the U value of the atoms to which they are linked (1.5 times for methyl groups). The Si-N-N-CH$_3$ coordinated to Fe1 is disordered over two positions. The site occupancies were freely refined and converged near a population distribution of 0.75/0.25. The major component has atom suffixes "4", the minor has "B". Due to the low occupancies, the thermal parameters of the minor component were constrained to be similar to their major counterpart with a small uncertainty (0.002). The disordered Si-N distances were restrained to be similar with a small uncertainty (0.002). No additional restraints were needed for a stable refinement. Two reflections were omitted from the least squares refinement due to detector artifacts. The full numbering scheme of compound 007-a23018 can be found in the full details of the X-ray structure determination (CIF), which is included as Supporting Information. CCDC number 2303073 contains the supplementary crystallographic data for this structure.

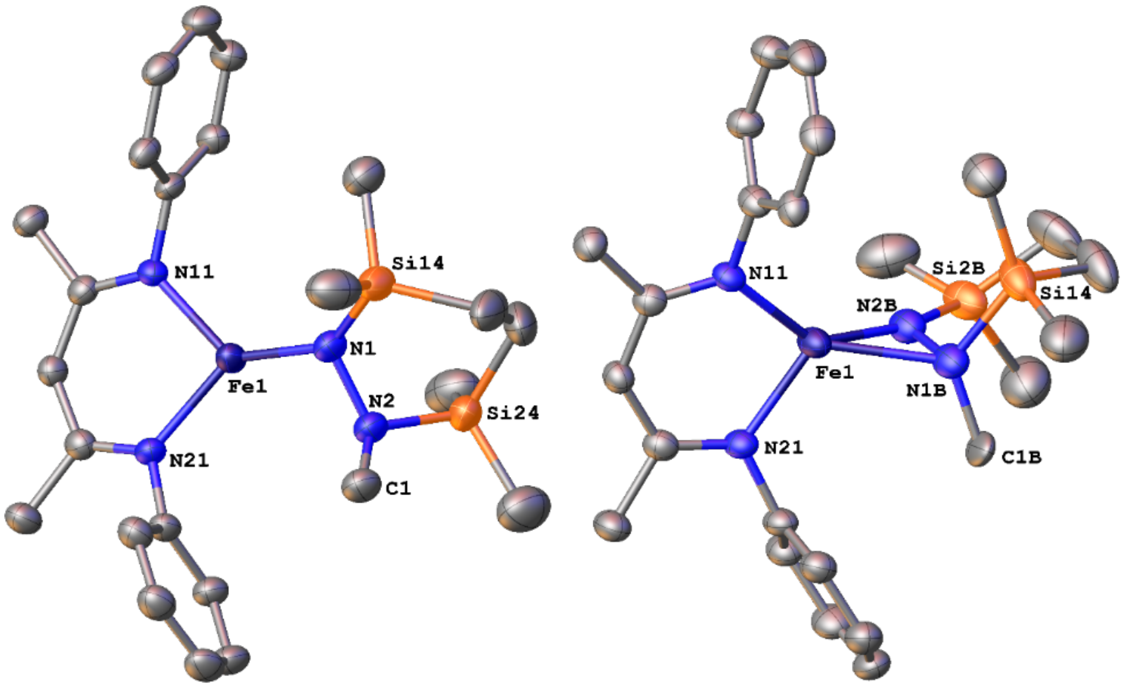

Figure S60. A complete numbering scheme of **10** with 50% thermal ellipsoid probability levels. The hydrogen atoms are omitted for clarity. The η^1 isomer (**10-1**, 75%) is shown on the left, and the η^2 isomer (**10-2**, 25%) is shown on the right.

Table S6. Crystal data and structure refinement for **10**.

Identification code	007a-23018	
Empirical formula	C36 H60 Fe N4 Si2	
Formula weight	660.91	
Temperature	93(2) K	
Wavelength	1.54184 Å	
Crystal system	Triclinic	
Space group	P-1	
Unit cell dimensions	a = 8.6993(4) Å	α= 88.974(3)°.
	b = 12.0566(4) Å	β= 82.392(3)°.
	c = 18.0449(6) Å	γ = 83.828(3)°.
Volume	1865.07(12) $Å^3$	
Z	2	
Density (calculated)	1.177 g/cm^3	
Absorption coefficient	4.065 mm^{-1}	
F(000)	716	
Crystal size	0.400 x 0.400 x 0.050 mm^3	
Crystal color and habit	brown plate	
Diffractometer	Rigaku Saturn 944+ CCD	
Theta range for data collection	2.470 to 66.587°.	
Index ranges	-10<=h<=9, -14<=k<=14, -21<=l<=21	
Reflections collected	57657	
Independent reflections	6463 [R(int) = 0.0834]	
Observed reflections (I > 2sigma(I))	5788	
Completeness to theta = 66.587°	97.9 %	
Absorption correction	Semi-empirical from equivalents	
Max. and min. transmission	1.00000 and 0.48700	
Solution method	SHELXT-2014/5 (Sheldrick, 2014)	
Refinement method	SHELXL-2014/7 (Sheldrick, 2014)	
Data / restraints / parameters	6463 / 48 / 440	
Goodness-of-fit on F^2	1.160	
Final R indices [I>2sigma(I)]	R1 = 0.0533, wR2 = 0.1468	
R indices (all data)	R1 = 0.0600, wR2 = 0.1613	
Extinction coefficient	n/a	
Largest diff. peak and hole	0.394 and -0.717 e.$Å^{-3}$	

LFeN(Ph)N(Me$_2$Si(CH$_2$)$_2$SiMe$_2$) (12)

Low-temperature diffraction data (ω-scans) were collected on a Rigaku MicroMax-007HF diffractometer coupled to a Saturn994+ CCD detector with Cu Kα (λ = 1.54178 Å). The diffraction images were processed and scaled using Rigaku Oxford Diffraction software (CrysAlisPro; Rigaku OD: The Woodlands, TX, 2015). The structure was solved with SHELXT and was refined against F^2 on all data by full-matrix least squares with SHELXL.[12] All non-hydrogen atoms were refined anisotropically. Hydrogen atoms were included in the model at calculated positions and refined using a riding model. The isotropic displacement parameters of all hydrogen atoms were fixed to 1.2 times the U value of the atoms to which they are linked (1.5 times for methyl groups). CCDC number 2226344 contains the supplementary crystallographic data for this structure.

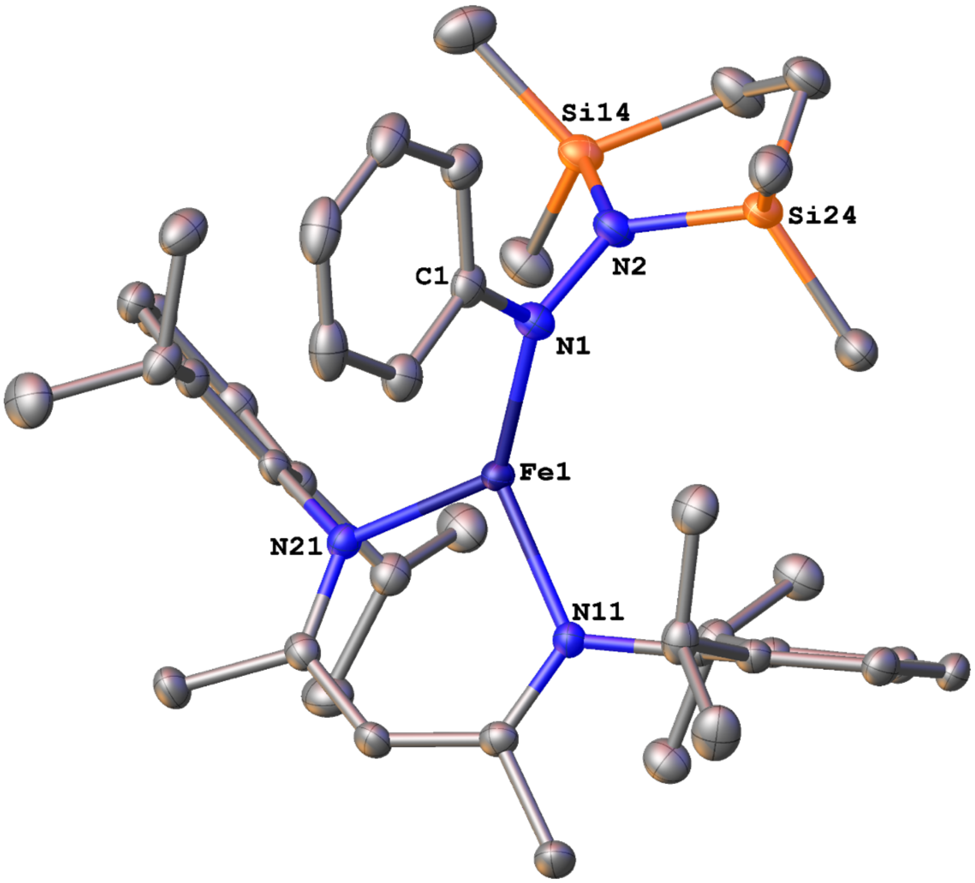

Figure S61. A partial numbering scheme of **12** with 50% thermal ellipsoid probability levels. The hydrogen atoms are omitted for clarity.

Table S7. Crystal data and structure refinement for **12**.

Identification code	007a-22007	
CCDC Number	2226344	
Empirical formula	C41 H62 Fe N4 Si2	
Formula weight	722.97	
Temperature	93(2) K	
Wavelength	1.54184 Å	
Crystal system	Monoclinic	
Space group	$P2_1/n$	
Unit cell dimensions	a = 11.9946(2) Å	α= 90°.
	b = 19.7217(3) Å	β= 95.2570(10)°.
	c = 17.1523(3) Å	γ = 90°.
Volume	4040.38(12) $Å^3$	
Z	4	
Density (calculated)	1.189 Mg/m^3	
Absorption coefficient	3.798 mm^{-1}	
F(000)	1560	
Crystal size	0.200 x 0.200 x 0.020 mm^3	
Crystal color and habit	Black Plate	
Diffractometer	Rigaku Saturn 944+ CCD	
Theta range for data collection	3.423 to 66.601°.	
Index ranges	-14<=h<=14, -23<=k<=23, -20<=l<=20	
Reflections collected	126800	
Independent reflections	7123 [R(int) = 0.0574]	
Observed reflections (I > 2sigma(I))	6762	
Completeness to theta = 66.601°	99.8 %	
Absorption correction	Semi-empirical from equivalents	
Max. and min. transmission	1.00000 and 0.79405	
Solution method	SHELXT-2014/5 (Sheldrick, 2014)	
Refinement method	SHELXL-2014/7 (Sheldrick, 2014)	
Data / restraints / parameters	7123 / 0 / 447	
Goodness-of-fit on F^2	1.037	
Final R indices [I>2sigma(I)]	R1 = 0.0295, wR2 = 0.0733	
R indices (all data)	R1 = 0.0313, wR2 = 0.0744	
Largest diff. peak and hole	0.379 and -0.318 $e.Å^{-3}$	

Computations

DFT calculations were performed using ORCA version 5.0.3. Structures were optimized using the B3LYP functional (unless otherwise noted) and ZORA-def2-TZVP basis set. All computations used the D3BJ dispersion correction and an implicit continuum solvation model. Minima were confirmed by the presence of only real frequencies. Transition states were confirmed by the presence of one imaginary frequency corresponding to N–C bond formation. Mössbauer calculations were performed on the optimized structures, and isomer shifts were derived using our established correlation for diketiminate complexes.[14]

Example input file for geometry optimization and frequency calculation:

```
! UKS B3LYP TightOpt ZORA-def2-TZVP ZORA RIJCOSX SARC/J
! TightSCF D3BJ CPCMC(toluene) NumFreq

%pal nprocs 20 end

%rel OneCenter true
end

%output
Print [ P_Basis ] 2
Print [ P_MOs ] 1
end

%scf MaxIter 800
 shift shift 0.3 erroff 0.0 end
 end

* xyzfile 0 5 LFeCH2TMSNNSiSi.xyz
```

Example input file which generated QRO files for plotting:

```
! UKS B3LYP UNO UCO NormalPrint ZORA-def2-TZVP ZORA RIJCOSX SARC/J
! TightSCF D3BJ CPCMC(toluene)

%pal nprocs 20 end

%rel OneCenter true
end

%output
Print [ P_Basis ] 2
Print [ P_MOs ] 1
end

%scf MaxIter 800
 shift shift 0.3 erroff 0.0 end
 end

* xyzfile 0 5 LFeCH2TMSNNSiSi_0_m5_B3LYP_def2-TZVP_OptHessFreq.xyz
```

Example input file which generated Mössbauer parameters:

```
! UKS B3LYP ZORA-def2-TZVP ZORA
! TightSCF D3BJ CPCMC(toluene)

%Method SpecialGridAtoms 26
 SpecialGridIntAcc 7 end

%pal nprocs 20 end

%scf MaxIter 800
 shift shift 0.3 erroff 0.0 end
 end

* xyzfile 0 2 LFeNNSiSi_0_m2_B3LYP_def2-TZVP_OptHessFreq.xyz


%eprnmr
 nuclei = all Fe {fgrad,rho} end
```

Example input file for NEB calculations:

```
! BP86 D3BJ ZORA ZORA-def2-TZVP
! UKS RI SARC/J
! NOPRINTMOS
! NEB-CI

%scf maxiter 800
end

%pal nprocs 20
end

%neb
NEB_End_XYZFile "LFeNMeNSiSi-m3-NEB.xyz"
Nimages 8
preopt_ends false
end

* xyzfile 0 3 LFeMeNNSiSi-m3-NEB.xyz
```

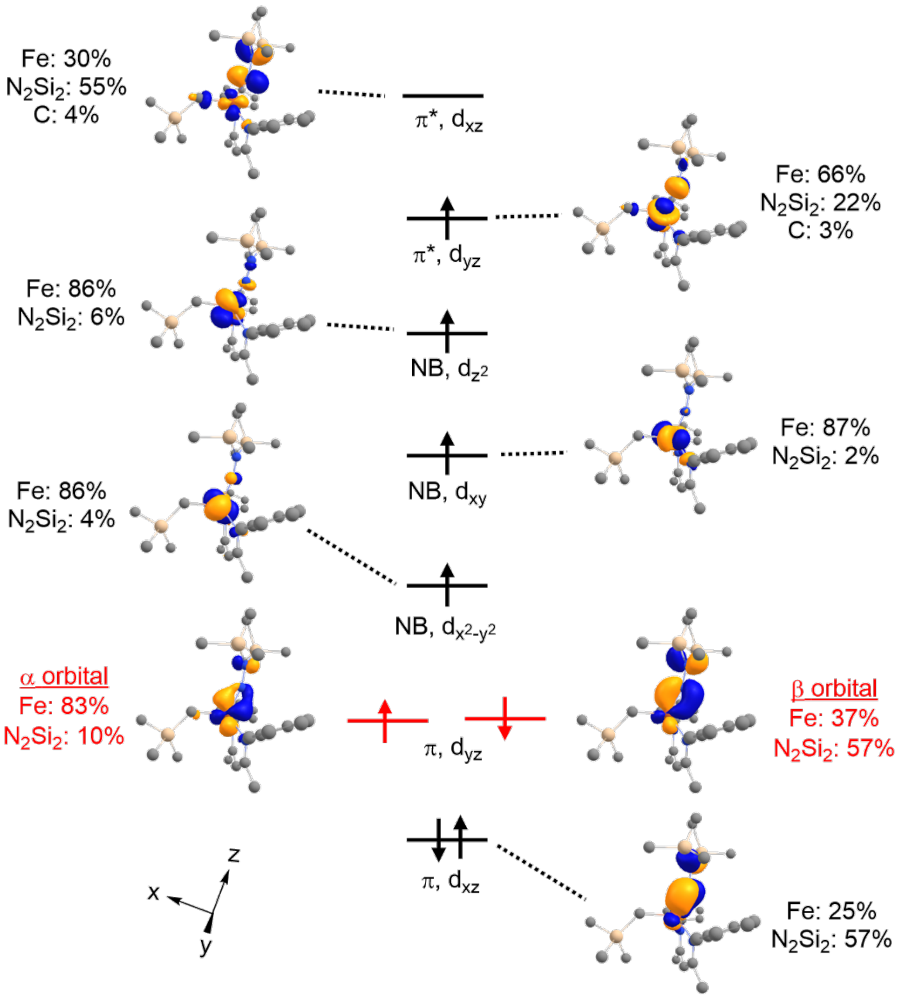

Figure S62. Qualitative molecular orbital diagram showing the QROs of **7a** (S = 2) with QRO plots shown at an isovalue of 0.05 au. In red are the one-electron orbitals for the corresponding pair in the spin-polarized Fe−N π-bond.

Table S8. Comparison of experimental and DFT-calculated bond metrics and Mössbauer parameters of complex **7a** (S = 1 and 2), as well as previously reported iron(IV) hydrazido(2–) complexes **2** and **7-CC^{t}Bu**.[15]

	**7a (Expt)**	**7a (Calcd S = 1)**	**7a (Calcd S = 2)**	**2**	**7-CC^{t}Bu**
Spin State	S = 2	S = 1	S = 2	S = 1	S = 2
Fe–N_{hyd} (Å)	1.749(2)	1.690	1.770	1.672(3)	1.7561(15)
Ave. Fe–N_L (Å)	2.051(1)	1.993	2.039	1.971(2)	2.028(1)
Fe–C (Å)	2.065(2)	2.042	2.069	1.999(4)	1.9877(17)
N–N (Å)	1.326(3)	1.300	1.292	1.340(4)	1.305(2)
τ_4	0.88	0.82	0.94	0.75	0.93
δ (mm s^{-1})	0.33	0.27	0.37	0.17	0.36
$\|\Delta E_Q\|$ (mm s^{-1})	1.58	2.30	1.63	1.49	1.83

Table S9. Comparison of bond lengths and Mössbauer parameters between the experimental structures of **8** and $LFeNN(SiMe_3)_2$ versus DFT computed parameters using BP86 and B3LYP. All computations used the def2-TZVP basis set.

Compound	Functional	Spin state	$Fe–N_2$ (Å)	Ave. $Fe–N_{nacnac}$ (Å)	Rel. Energy (kcal mol^{-1})	δ (mm s^{-1})	$\lvert \Delta E_Q \rvert$ (mm s^{-1})
**8**	Exp.	1/2				0.13	2.13
		3/2				0.51	1.75
	BP86	1/2	1.627	1.872	0	0.12	2.04
		3/2	1.689	1.911	9	0.48	1.83
	B3LYP	1/2	1.653	1.914	2	0.24	1.76
		3/2	1.731	1.980	0.0	0.67	1.36
$LFeNN(SiMe_3)_2$ (ref 5)	Exp.	1/2	1.638(2)	1.924(1)		0.22	1.99
		3/2	1.671(2)	1.948(1)		0.46	1.16
	BP86	1/2	1.628	1.885	0.0	0.14	1.93
		3/2	1.690	1.919	9	0.51	1.72
		5/2	1.774	1.962	31	0.61	3.69
	B3LYP	1/2	1.660	1.924	1		
		3/2	1.725	1.972	0		
		5/2	1.850	2.002	15		

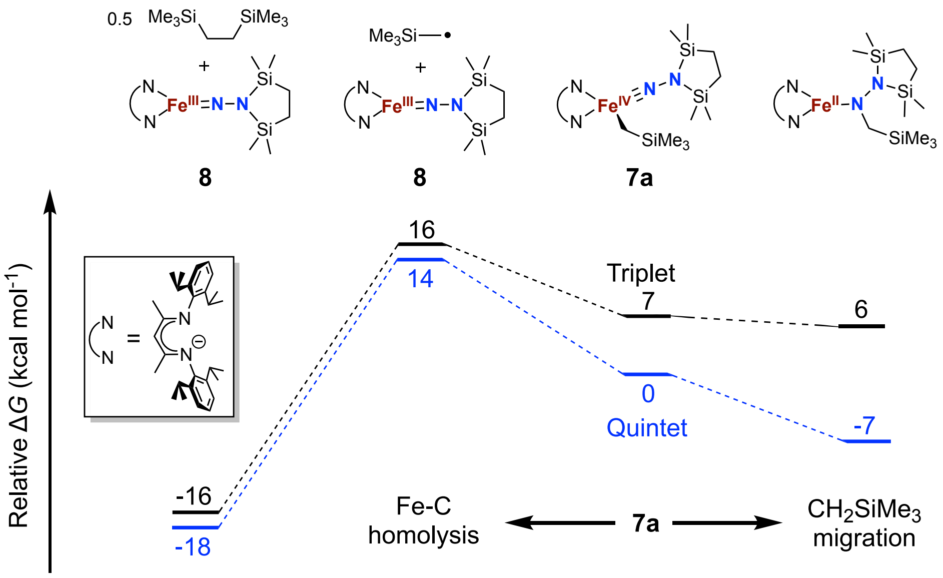

Figure S67. B3LYP/def2-TZVP calculated potential energy surface showing the competing reaction pathways of Fe–C bond homolysis and CH_2SiMe_3 migration starting from **7a**. Transition states for the migration pathway could not be found.

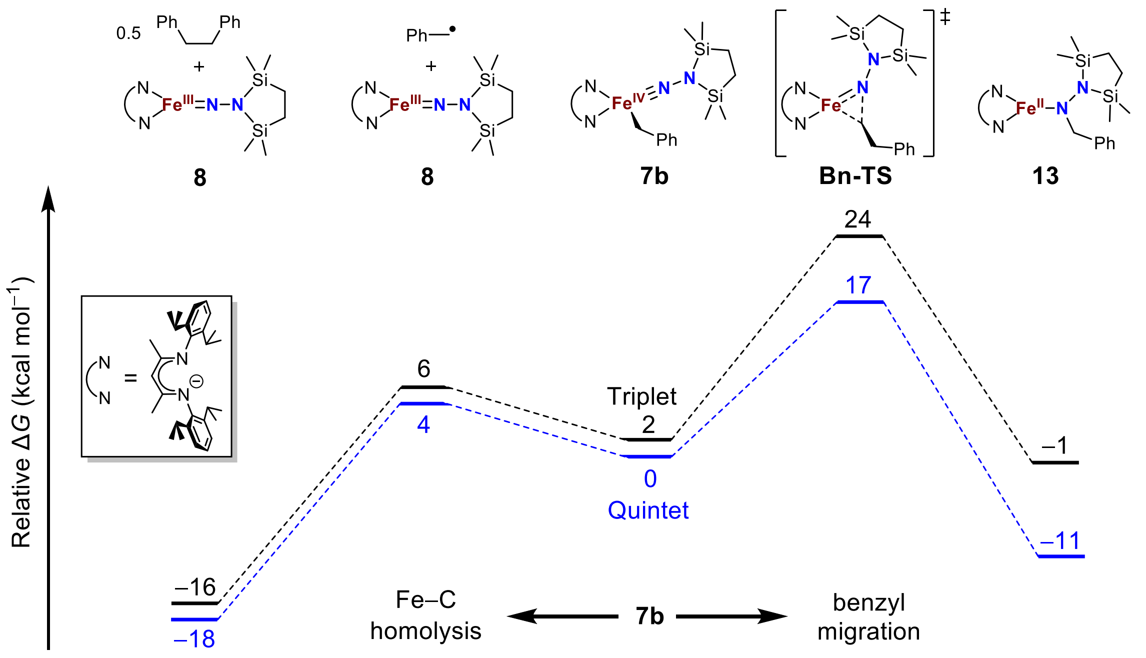

Figure S68. B3LYP/def2-TZVP calculated potential energy surface showing the competing reaction pathways of Fe–C bond homolysis and benzyl migration starting from **7b**.

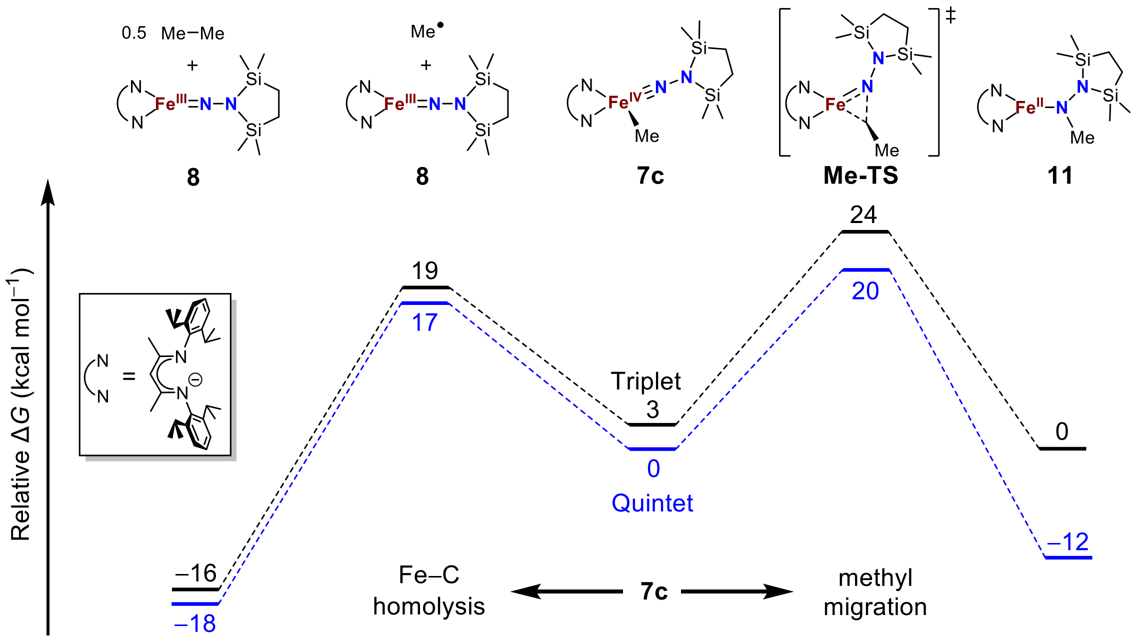

Figure S69. B3LYP/def2-TZVP calculated potential energy surface showing the competing reaction pathways of Fe–C bond homolysis and methyl migration starting from **7c**.

Table S10. Comparison between experimental and calculated bond lengths for **10**. The calculated distances for the triplet η^2 structure are given only for the lowest-energy conformer. We were unable to optimize the η^1 isomer in the triplet ground state.

	Atom Pair	Experimental Distance (Å)	DFT-Optimized Distance, $S = 2$ (Å)	DFT-Optimized Length, $S = 1$ (Å)
η^1	Fe1–N1	1.900(3)	1.88	
	Fe1···N2	2.639	2.64	
	N1–Si14	1.745(3)	1.74	
	N2–Si24	1.734(5)	1.75	
η^2	Fe1–N1B	2.208(12)	2.26	2.04
	Fe1–N2B	1.983(10)	1.91	1.85
	N1B–Si14	1.770(10)	1.78	1.80
	N2B–Si2B	1.760(13)	1.73	1.73

Table S11. Calculated ΔG and Mössbauer parameters for optimized η^1 and η^2 isomers of **10** with $S = 1$ and 2, along with the experimental Mössbauer spectrum fit parameters assigned to each species. The table corresponds to the three-component fit in Figure S22. The fit was obtained by first inputting the calculated Mössbauer parameters for the three lowest-energy isomers. The percent composition, δ, and $|\Delta E_Q|$ of each doublet was then allowed to vary, while Γ was fixed to 0.50 mm s^{-1}. Enabling Γ to float always resulted in at least one doublet broadening to unphysical values of $\Gamma > 3$ mm s^{-1}. Thus, a Mössbauer doublet line width comparable to hydrazido species **12** was selected for each component.

Species	ΔG (DFT, kcal mol^{-1})	Percent of exp. fit (%)	δ exp. fit (mm s^{-1})	δ calcd (mm s^{-1})	$\|\Delta E_Q\|$ exp. fit (mm s^{-1})	$\|\Delta E_Q\|$ calcd (mm s^{-1})
η^1 ($S = 2$)	0	54	0.68	0.60	0.96	0.95
η^2 ($S = 1$)	4	37	0.55	0.60	1.28	1.16
η^2 ($S = 2$)	3	9	0.98	0.91	1.45	1.49
η^2 ($S = 1$)*	17	0	n/a	0.69	n/a	1.08

* We also obtained a second conformer, which has a different conformation in the six-membered ligand ring. Since it was calculated to have a higher energy and was not needed to explain the Mössbauer fit, it is not discussed further here. We were unable to optimize the η^1 isomer in the triplet ground state.

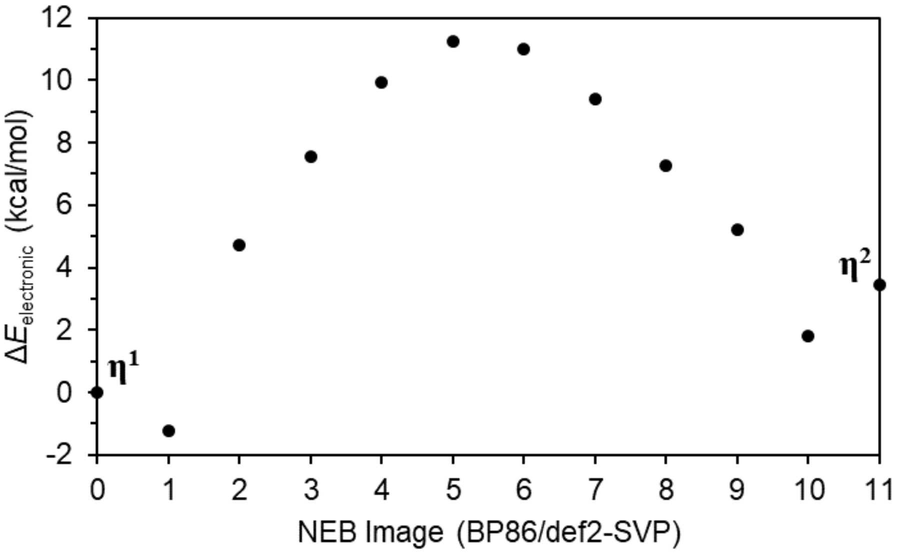

Figure S70. Electronic energy during isomerization of **10** between η^1 and η^2 structures on the quintet surface. The plot was generated using the NEB method at the BP86/def2-SVP level of theory. The starting and ending structures were obtained from optimization of $S = 2$ η^1 and η^2 structures using B3LYP/ZORA-def2-TZVP, and they were not re-optimized at the NEB level of theory during the energy scan (hence the starting and ending structures are not minima on this surface). This scan demonstrates that there is a low-energy pathway between isomers, and they could be interconverting readily in solution and during crystallization.

CASSCF Computations

Complete active space self-consistent field (CASSCF) calculations were performed on a single root to interrogate whether a multiconfigurational nature of the triplet ground state of compound **5a** may be present. The active space consists of 8 electrons in 7 orbitals (CASSCF(8,7)), which were chosen from the DFT-generated QROs to include all Fe-based orbitals and N-N pi system. The def2-TZVP basis set was used,[16] and relativistic effects added with the zeroth order regular approximation (ZORA).[17] The RIJCOSX approximation was implemented to decrease computational cost,[18] with the accompanying auxiliary basis formulated using the AutoAux keyword. All CASSCF-derived orbital plots show the natural orbitals.

The dynamic electron correlation energies of the triplet and quintet ground states were then added using strongly-contracted second-order *n*-electron valence state perturbation theory (NEVPT2), and computational cost was improved with the resolution of identity (RI) approximation.[19] Thermal corrections from the B3LYP/def2-TZVP DFT calculations were added to the single-point CASSCF/NEVPT2 energies.

Occupation number is listed at the bottom left of each orbital.

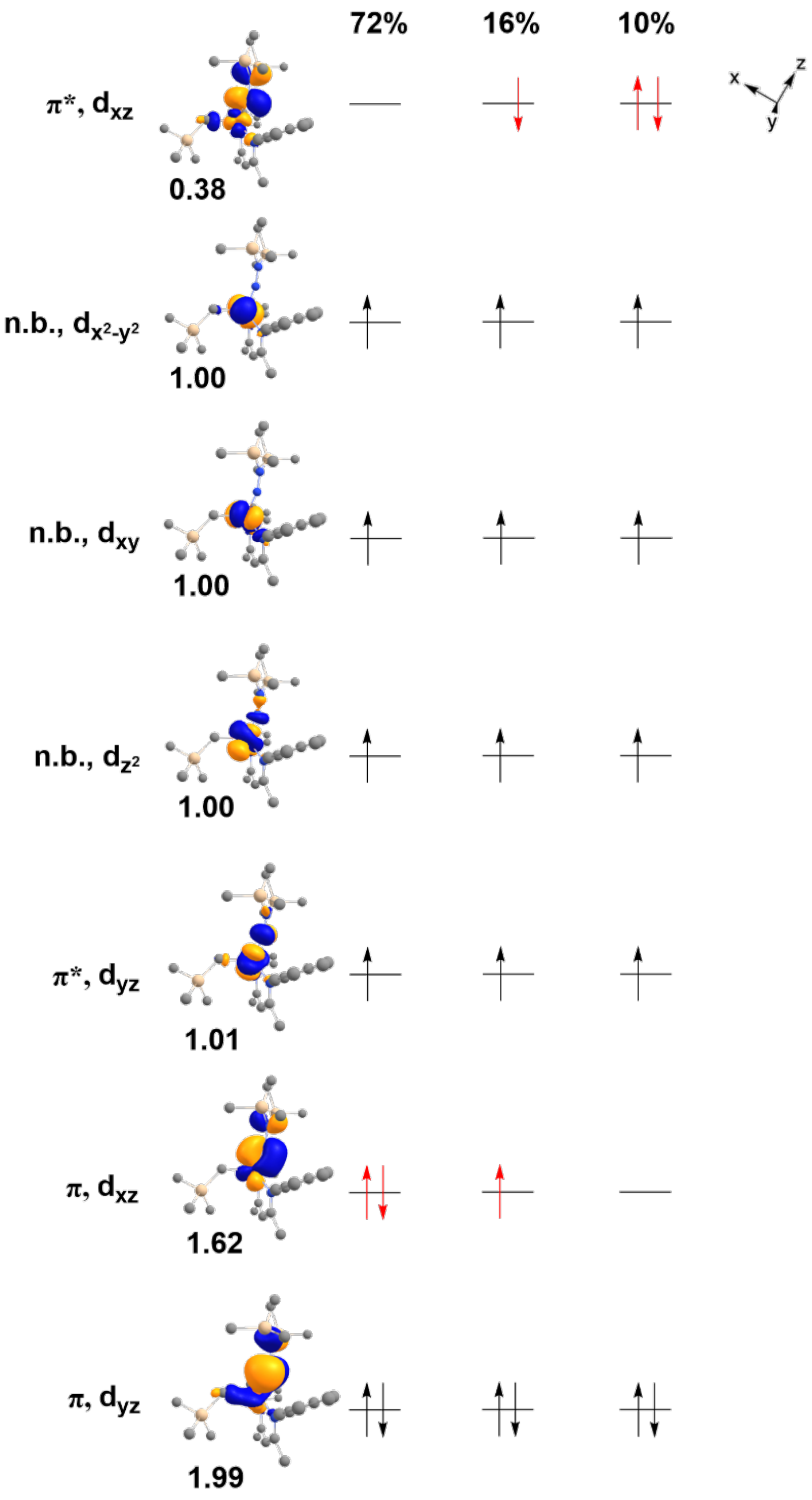

Figure S71. Plots of key frontier orbitals from the CASSCF(8,7) calculations on quintet **7a**, along with the occupation numbers of correlated orbitals in the top three configurations.

Atomic coordinates of the optimized structure of **7a** ($S = 2$)

Fe	6.14975634046805	7.28191140615222	14.69290731168636
Si	4.55759919836630	4.50397051253663	17.19289914659202
Si	5.88576929119330	6.46939070356343	19.05226436405086
Si	3.78212448115726	8.42057050259401	12.32194951521427
N	6.07868528190991	6.58217436695021	16.31691727714335
N	5.60703057975934	5.96038579739666	17.34708205333941
N	7.47032159990921	6.32929907831148	13.47026718673451
N	7.23416818604997	9.01154395901461	14.70600007754025
C	8.21871445625720	10.79950806699117	13.34776498592620
H	8.80703370113272	11.20004791580857	14.17070502855379
H	7.30905547770071	11.40141069995987	13.28867705799551
H	8.77648486594074	10.91942371223113	12.42216429604050
C	7.84605572370108	9.35490835271089	13.57807003678211
C	8.17476163624739	8.44867296506037	12.55932710049112
H	8.65236007961213	8.88397354748371	11.69449972613551
C	8.11347479243200	7.04229068466203	12.55765976828127
C	8.84106011748391	6.34982538301311	11.42970733496456
H	9.32069300244817	5.43345698198728	11.76794642786030
H	9.58885000063912	7.00639634944964	10.99088572066432
H	8.12961775944599	6.07400702136843	10.64877480958236
C	7.27806199088506	9.89459693785237	15.82434869003840
C	8.46040562648152	9.96430824216270	16.59075180202407
C	8.50976763029806	10.83917994483635	17.67324599900125
H	9.41764168665058	10.90591472949785	18.25970498539981
C	7.41802240223039	11.62016374355878	18.01984419960034
H	7.47472142292096	12.29494948831941	18.86488679228584
C	6.24958520502809	11.51564532022734	17.28433850124937
H	5.38968957746402	12.11108403949954	17.56447441004447
C	6.15583705111409	10.65989645682398	16.18792385849271
C	9.67109506638143	9.09721521784792	16.29137313299279
H	9.41697971848833	8.44226558996031	15.46037149989131
C	10.00164041648972	8.20871473637747	17.49412559527727
H	10.25407369183973	8.80442181107647	18.37337261139907
H	10.85380479109300	7.56519313910246	17.27186013759689
H	9.15124592414877	7.57740016030250	17.74618411117786
C	10.89863311901950	9.92014090539386	15.88115814023920
H	10.70282494889146	10.52276804072679	14.99405512063955
H	11.73823478492509	9.25787729762152	15.65812432983007
H	11.20721229814104	10.59360174140336	16.68384505461473
C	4.84829417881328	10.58346705646871	15.42764791358540
H	4.99298438722279	9.89393938142199	14.60065618182840
C	3.72781739056518	10.02105402652801	16.31053570381249
H	3.53808715074633	10.67299656846374	17.16599511146023
H	3.98975585773452	9.03278370842853	16.68918517136276
H	2.80083774392604	9.93155628545505	15.74143687657726

C	4.44988870429490	11.94126776357914	14.83855332004483
H	3.55063668928955	11.84093443500449	14.22773313080271
H	5.24407988564822	12.34726363325323	14.20966600580999
H	4.24161734406878	12.66999875590693	15.62495964096123
C	7.63466832785539	4.91120731313182	13.52522241963713
C	8.59195861487968	4.38258300845809	14.41563313051496
C	8.80094722258367	3.00648581707792	14.43513176747858
H	9.54526754116028	2.58956417087868	15.09945287577627
C	8.06681370583667	2.15457087959948	13.62347910598671
H	8.24661000807115	1.08695497378963	13.64910889945779
C	7.08572049723024	2.67886163608347	12.80125196491887
H	6.48794652723946	2.01133646254055	12.19372760344572
C	6.84350088358936	4.05179566488564	12.74253587778327
C	9.39654174420405	5.28793938857464	15.32971146007295
H	8.79781274441918	6.18316719327476	15.49795738039753
C	9.66280372865144	4.64786550041972	16.69454430411954
H	8.73469886376649	4.30202577474977	17.15166200824522
H	10.12011866881958	5.37321549824402	17.36608161055679
H	10.34487330507640	3.79871785557032	16.62162359931159
C	10.71003047197708	5.72182306884492	14.66491359330801
H	11.32483495490344	4.85045232618245	14.42599187818887
H	11.28408924340621	6.36885527132285	15.33077739890881
H	10.52771267773203	6.27099069331871	13.74148612172818
C	5.70686544562645	4.55164905559246	11.87319733954496
H	5.68479288648957	5.63705905039883	11.94601255519156
C	4.36660585240857	4.01467646442263	12.39540495952714
H	4.32573091521626	2.92672587121836	12.30983126677727
H	3.53640553380024	4.42859694029832	11.82195454608831
H	4.21898480348802	4.27152655274489	13.44366571181592
C	5.88208423578739	4.17290851312110	10.39762833702495
H	6.83540452880182	4.52224994476405	9.99986340385794
H	5.08145435000118	4.61023210335027	9.79816578516201
H	5.84414478041149	3.08976822035448	10.26398744592203
C	4.35814929730328	4.15476221429760	19.03557822908775
H	3.42201209823178	3.63442445313984	19.25444226414653
H	5.16464821048687	3.48973486456712	19.35866945567641
C	4.45046023775732	5.50712227850557	19.79824097872708
H	4.55469610374276	5.35466743201238	20.87549794239919
H	3.53083461982475	6.08145464111089	19.64912131022663
C	5.45730987075662	3.18756305638802	16.23534425844540
H	4.82492453867241	2.30962900202030	16.08290235014054
H	6.35887622623630	2.87318379076533	16.76388547331815
H	5.75996305414928	3.55882187929976	15.25598322435458
C	2.95137878829265	5.00704740890885	16.38777845163796
H	2.43511546315005	5.75642465258531	16.99170234206321
H	2.29115754718432	4.14326431917188	16.27576695673443

H	3.12864421076530	5.43541390776234	15.40143721501159
C	7.51411925840611	5.78055315197824	19.66100330483008
H	7.54769762294050	4.69549032230871	19.54463545901853
H	7.64365784150025	6.01203376661956	20.72176677578517
H	8.36002022885985	6.20261591713433	19.11942002874925
C	5.82485135571224	8.32061827440845	19.19009255798129
H	6.55638780116301	8.77959053852995	18.52335971705647
H	6.05377000406584	8.63774359106707	20.21035316262970
H	4.84037674531544	8.70624389222230	18.92345364853455
C	4.27237743312655	7.45109788904338	13.83984182650616
H	3.98036969021216	6.41165871848801	13.65839476212125
H	3.64110608564235	7.79058418513078	14.66716139888232
C	2.09898637695297	9.23280815945281	12.61050496420924
H	1.76119709389174	9.77328817297986	11.72166540413785
H	2.13995264351590	9.94569370204107	13.43767289437726
H	1.34209712112643	8.48366832302903	12.85790199216187
C	4.95097872855761	9.78980594214441	11.76278266548210
H	5.92717636648895	9.38368205211248	11.49534134548670
H	5.10527072569436	10.56042158694764	12.51676900335971
H	4.52924748241180	10.27381766164785	10.87688450095627
C	3.59897962662767	7.30672484911987	10.80810700863313
H	4.55956980389038	6.89888206163854	10.48850097619572
H	3.19268612239011	7.88503308595571	9.97355293526583
H	2.92306225486240	6.47002087790136	10.99529661129281

Atomic coordinates of the optimized structure of **7b** (S = 1)

Fe	6.55554711535792	7.15188362495727	14.81328469704951
Si	4.37179993099119	4.86810194480583	17.21590404789410
Si	5.89701222531039	6.63806618222308	19.10624181124444
N	6.21730418139824	6.84849406636859	16.46020702541862
N	5.56426727541311	6.19768697093522	17.39577039710260
N	7.70827623315142	6.40320311975524	13.33130650832411
N	7.30061448981514	9.01101016261967	14.55112794340397
C	8.20593897972830	10.87585026850610	13.23703113056351
H	7.28654969331081	11.44661640355537	13.37462378101457
H	8.58900979545864	11.05482391114590	12.23462017015381
H	8.92805657438445	11.26145357307524	13.95766275556390
C	7.94307115084584	9.40393252714096	13.45412670342887
C	8.44498589374560	8.52522111106225	12.49055958179177
H	9.00117881859746	8.98039817142420	11.68327556654406
C	8.46092914966555	7.12020813341656	12.51414757922074
C	9.42512298632669	6.46837172621792	11.54884161067089
H	10.43260282168759	6.84458011916548	11.73316358243209
H	9.16451596543484	6.73077019339345	10.52228207619720
H	9.43666430228969	5.38670130139648	11.63929745595431
C	7.24342201778281	9.92102142206590	15.65013741623720

C	8.39868325744983	10.08153901185962	16.44422548461618
C	8.35955885091624	10.96799534964855	17.51700029536274
H	9.24054107410965	11.09344107488159	18.13398203275783
C	7.21055116329446	11.68641364025463	17.81397041013158
H	7.19736375900691	12.37530148048136	18.64951472857553
C	6.07474245235143	11.49836548372087	17.04454999939959
H	5.17099949857480	12.04241998854029	17.28909763177440
C	6.06566092804280	10.61581263851734	15.96452563932502
C	9.66108426261622	9.27303399477287	16.19610742676122
H	9.54626540094827	8.74582118123145	15.25156876774238
C	9.82287313912534	8.21066874606149	17.28921119436732
H	9.94864484663376	8.67486470036142	18.27023726896409
H	10.69654823481495	7.58500789066017	17.09418624603488
H	8.94208361159281	7.57130444100891	17.32583182866778
C	10.91743384015769	10.14312534964472	16.08666848803068
H	10.81568708529617	10.89467046883789	15.30172261915526
H	11.78442181365596	9.52218994864916	15.84980902078307
H	11.12974209216963	10.66360469224668	17.02284721995206
C	4.78417985359390	10.41423689925701	15.18365225850699
H	4.99836592655832	9.74047403077002	14.35845617920691
C	3.72588709259459	9.74562827638590	16.07071652469963
H	3.51399425478153	10.35397623698837	16.95318587059846
H	4.07116718792501	8.76723640753302	16.40656208067916
H	2.79479099404490	9.61098463035529	15.52028538000648
C	4.25619696754023	11.72027229286478	14.58144741982833
H	3.36973955982845	11.52142267731655	13.97636322446330
H	5.00546511329570	12.18748238723859	13.93928333623097
H	3.98305116573977	12.44054650825221	15.35617400026270
C	7.75306733219918	4.97426078670308	13.32582666650914
C	8.48484242406500	4.32229165349104	14.33962756293126
C	8.47501044902228	2.93111561434967	14.38979240207874
H	9.04365660392786	2.42361367353585	15.15778678021022
C	7.75318053147011	2.18327881305679	13.47309066332288
H	7.75038573016138	1.10190663167086	13.53102361886566
C	7.04298422229308	2.83117275502762	12.47668788512260
H	6.49078272885155	2.24575843199721	11.75266686767507
C	7.03030523749080	4.22224721992393	12.37434313170209
C	9.33025094573780	5.09780855526754	15.33194881583463
H	9.00827192475262	6.13734241246770	15.30199530429184
C	9.14427850374319	4.60743166892142	16.76832552276327
H	8.09487156250364	4.65267491348669	17.05763688773430
H	9.71103786214737	5.23494385912349	17.45604106686299
H	9.49284486181636	3.58106232989602	16.89817489744775
C	10.80832376904186	5.06714949676559	14.92073940220040
H	11.19748011398931	4.04625166828276	14.94385863695580
H	11.40774132425473	5.67540097957777	15.60195013490373

H	10.94466621278719	5.45406690068881	13.90980965535818
C	6.28493395217367	4.85137525874202	11.20820825293275
H	6.30200014244495	5.92957909988865	11.34233560306552
C	4.81576034670020	4.41457547971750	11.12693268527503
H	4.73318237712229	3.34984209987421	10.89919967134105
H	4.30974715624773	4.96471706989885	10.33118908673790
H	4.27921879123533	4.60158794995366	12.05571724680222
C	6.97547023875148	4.53161156828903	9.87246221012832
H	8.02395782789364	4.82356548855369	9.87557353549712
H	6.47648649885724	5.05755786195899	9.05539950795758
H	6.92709629474420	3.46108583223759	9.66066974130214
C	4.38415337654552	4.33231130069710	19.02092529152714
H	3.49115740657005	3.75865698063380	19.28271186375243
H	5.24176464391116	3.67526673073715	19.19471163596622
C	4.51880655929932	5.61712101185307	19.88065734768413
H	4.71797961253807	5.38496030588609	20.93013091302079
H	3.58451703502316	6.18581086174644	19.85551953998291
C	5.01550049335455	3.59496539289694	16.01769809315824
H	4.31192101643930	2.76217453897084	15.93687730532334
H	5.97593455259563	3.19993064323733	16.35173472122232
H	5.15835228274750	4.01117538648270	15.02081149752130
C	2.64830619033011	5.47013303856956	16.77687914150271
H	2.47005813163888	6.46843135299415	17.18155010665443
H	1.91165307288245	4.79285382593211	17.21828835383706
H	2.46828336069182	5.50323960903967	15.70277212534495
C	7.56256126755463	6.02708599411512	19.69217652440868
H	7.64915095013217	4.94440078137264	19.58701862275953
H	7.68018509238945	6.27586132236917	20.75081041553799
H	8.38293554865294	6.48994522616301	19.14594316487962
C	5.75942664876177	8.48312672827154	19.29035020083212
H	6.49237754000050	8.98692861143206	18.65848957575396
H	5.94031712317067	8.78534019613765	20.32496018014637
H	4.76944231309707	8.83496395017706	18.99611122260129
C	4.78143200379876	6.60487030069407	14.02547493518718
H	5.06691991626136	5.68054167785917	13.52845907705391
H	4.05483077995691	6.38141002656614	14.79542323060497
C	4.23447279153829	7.61902749029854	13.09231603335165
C	4.98415685201778	8.17142082217607	12.04511942799116
C	2.91732491948065	8.07218517708190	13.24842613751038
C	4.44743649442681	9.13619542011858	11.20462853816537
C	2.37485580342441	9.03307667377873	12.40623532615678
C	3.13836028374748	9.57475459519374	11.37791207497251
H	6.01250353100534	7.87571626600865	11.90948031418292
H	2.31509349137168	7.66161244526870	14.04931469324546
H	5.05953783292756	9.55314602287807	10.41382635400626
H	1.35376967367766	9.36302156223460	12.55579953655545

H	2.72103619024986	10.33035449420034	10.72438916467603

Atomic coordinates of the optimized structure of **7b** ($S = 2$)

Fe	6.37660110739378	7.21852381430866	14.77171292895447
Si	5.03909789887598	4.20495662122528	17.33636244748605
Si	5.21145221812418	6.82927131421234	18.80208865990971
N	6.26783087401309	6.43328906073313	16.32866473193725
N	5.66960738318425	5.88710278864666	17.34145224258016
N	7.59573225932146	6.34505259082147	13.37594640448937
N	7.36820380112614	9.00083702156295	14.72957325709037
C	8.73038614083564	10.71302693689540	13.59894680946320
H	8.22197705410914	11.44605384306179	14.21815356578768
H	8.75055911170506	11.06296300710350	12.56752514805322
H	9.76419001001084	10.64388460839726	13.94287882754294
C	8.08418718757377	9.34820919283937	13.67076265934240
C	8.36597431125181	8.49519648427536	12.59156594907242
H	8.90382840954892	8.95783255197907	11.77654539755137
C	8.24293041787480	7.10031508780486	12.49752852636445
C	8.92540654449990	6.47868252686619	11.29896536329779
H	9.13513938242016	5.42291280034732	11.44387565121830
H	9.85421028514184	7.00083112847387	11.07542779261217
H	8.27553286920852	6.57652283287096	10.42684559420577
C	7.35223831270096	9.83555646761413	15.88596091571134
C	8.43865278348200	9.79542495614112	16.77794966248143
C	8.38952187737842	10.58146331168781	17.92878242816392
H	9.21750269503845	10.55333704747157	18.62613652408568
C	7.30068091103140	11.39288143183002	18.19604395748928
H	7.27902615903436	12.00014624656611	19.09251846663234
C	6.23384028952061	11.42319205038732	17.30866385223813
H	5.38533426573110	12.05454883331044	17.53019286899097
C	6.22939139393576	10.64839650237529	16.15103556010607
C	9.65366193366700	8.91562075732873	16.54007793388335
H	9.54451865904139	8.43880556862923	15.56761102598009
C	9.74008498314160	7.80111998730500	17.58736240860135
H	9.79878472723467	8.21513850991779	18.59597943381025
H	10.63451149619137	7.19912212731291	17.42126500553872
H	8.87022000000358	7.14647323944004	17.53829429792418
C	10.95666299910889	9.72629105782363	16.52076092102756
H	10.91070769650180	10.54414992221803	15.80089646807667
H	11.79644522483685	9.08155304291867	16.25228227082210
H	11.17018693747888	10.15780747808184	17.50099238028709
C	5.04697641879134	10.68335198969490	15.19673040885923
H	4.86488177414787	9.65681321851885	14.88035703654723
C	3.75456352314540	11.19893055207868	15.83197285455360
H	3.81552005626225	12.26598301968043	16.05949858375604
H	3.50727833347199	10.66943014153799	16.75238151682355

H	2.93174991618048	11.06193168028995	15.13019902695378
C	5.34006654575135	11.49204657181460	13.92502243874615
H	4.44812872743197	11.53078223612734	13.29880788899030
H	6.13175591663524	11.04310485146104	13.33260777146557
H	5.63123357749848	12.51488905883512	14.17820091458005
C	7.72602423991152	4.92240431172670	13.33353664818818
C	8.72748225809523	4.32155790779570	14.12682304825899
C	8.89703287396962	2.94191512441222	14.05522967985882
H	9.66766325924401	2.46766092179707	14.64639901927780
C	8.08201179719563	2.15498388293280	13.25438927451841
H	8.22844830099652	1.08269550012062	13.21535305078736
C	7.06846776399135	2.74885015446650	12.52379155418880
H	6.42011967116458	2.13294984343473	11.91349031929739
C	6.86934296380939	4.12997857881560	12.54703217994409
C	9.61048631724007	5.15733531888146	15.03814046536482
H	9.01092083516817	6.00812135886140	15.36302138081879
C	10.05432202095199	4.40143197074930	16.29314264502660
H	9.20124387021763	3.98834453500488	16.83176092380087
H	10.58317054696106	5.07668994408793	16.96496333549011
H	10.73605484359778	3.58222916546160	16.05640546924405
C	10.83789876427492	5.70927735314866	14.29948949677268
H	11.44703205710564	4.89263998041187	13.90397856570085
H	11.45781572651592	6.29477222783587	14.98250486246538
H	10.55585908163085	6.35605955787026	13.47091491276011
C	5.74864387499713	4.71594219445965	11.70980206537476
H	5.69227594725898	5.77745835928510	11.93339855393016
C	4.39106905144376	4.08970169799270	12.05651768902347
H	4.33716293173116	3.05293605479864	11.71756684600953
H	3.58547721166052	4.64377266352100	11.57069620621114
H	4.20785110571982	4.09630052168175	13.13088063606157
C	6.01305185683580	4.56823687255111	10.20505096325915
H	6.96295856080958	5.01449390605223	9.91583949647107
H	5.21916323628111	5.05429359099118	9.63339608976971
H	6.03826495343909	3.51460425903967	9.91767008171468
C	4.75309725263424	4.08154030274671	19.19175835689499
H	4.06204473044266	3.28041761963392	19.46558327755300
H	5.70981216131283	3.84660261738636	19.66904769801235
C	4.24367562510006	5.46659417598464	19.68089486426209
H	4.30824244638395	5.55546253520605	20.76853485105682
H	3.18584407583201	5.58026920358070	19.42529081729721
C	6.27896610610983	3.02526775393829	16.61425988830067
H	5.87549402649079	2.00965794597763	16.60617048381522
H	7.20724048459840	3.01945474417142	17.18657682438339
H	6.51770079957720	3.29917112617927	15.58600864237925
C	3.43406065469736	4.16094289590018	16.37793092878993
H	2.70566692682241	4.85792576339042	16.79725479461700

H	3.00006857918707	3.15783810149786	16.40053889326256
H	3.59842194432512	4.43593799799906	15.33530807197891
C	6.72988008271683	7.39117327218194	19.72633223230883
H	7.40249686845504	6.55859787874659	19.94093963114195
H	6.44067102707206	7.85125029943599	20.67512807947006
H	7.27583213014607	8.13596711924024	19.14623234568871
C	4.18183255674454	8.28686398849542	18.27535360299202
H	4.78574069161913	8.95656363933653	17.66320406824281
H	3.82755292827761	8.85350886434045	19.14000376084174
H	3.31591160921493	7.96946006378481	17.69155456239771
C	4.40165567608839	7.11264626493473	14.16625098329542
H	4.44195204878454	6.10274246169681	13.75288757068153
H	3.73282607130845	7.08702526555464	15.02686130072462
C	3.93266689292311	8.08354996435559	13.14749071798716
C	4.65904481186672	8.34745914316214	11.97564634290985
C	2.73794350079373	8.79492784439028	13.32789531549956
C	4.20370573996437	9.25263431529886	11.02759614750104
C	2.28258519044968	9.70787445253309	12.38546989564162
C	3.00980924526009	9.94041671444283	11.22311351090080
H	5.61046862725612	7.85783947077433	11.81656705481547
H	2.15851963833819	8.62095267740934	14.22714084085478
H	4.79308031798094	9.43244424264666	10.13626179591084
H	1.35437942543170	10.23949893118338	12.55934448579666
H	2.65776155897598	10.65165471244564	10.48671567010910

Atomic coordinates of the optimized structure of **8** ($S = 1/2$) using BP86

Fe	3.89519131489873	15.06709620413389	11.25859593882010
Si	3.80250664729205	13.59054215067689	15.19771026720429
Si	3.56285977137170	16.60832506788962	15.14023710221821
N	3.82729590803603	15.07324843475921	12.88400404053038
N	4.12761911837188	16.40818193761775	9.97255305485637
N	3.77054128020090	13.71741092848608	9.96935809507577
N	3.76876412171365	15.08688893274013	14.19659429531699
C	4.21239415968312	16.28829371097367	8.63499386224348
C	3.83166069267565	13.82038076445669	8.62929900145897
C	2.33281027002284	12.01566646687095	10.99283876770495
C	2.22552351859220	10.81826547709176	11.71072887532208
H	1.24210360135132	10.48189072771587	12.04536343909872
C	3.61731476943203	12.43731307696031	10.58724948889967
C	1.09732882762468	12.83958024880247	10.66782574369448
H	1.42591353707956	13.67752199861845	10.03662959629519
C	5.42399992460204	18.13448599442102	11.13497112567666
C	4.76769624617137	11.65290318065726	10.83648347132745
C	7.00977696265886	10.96393267980717	9.84864643502171
H	7.30371250827342	10.28285981080671	10.66115312684603
H	7.93366273583318	11.36050019221209	9.40289624777824

H	6.48568275301220	10.37203564967851	9.08403012667885
C	6.13615334852946	12.11621387488331	10.35872412725885
H	5.96895640857977	12.80236090213778	9.51593870700324
C	4.28394651758081	20.11193341474906	11.97653958068906
H	4.31183190074799	21.04909076868174	12.53482576538429
C	4.20031465177804	17.69903373302585	10.58308743272275
C	4.49027776934533	17.51444952044328	7.80138220318146
H	3.69179495354793	18.26465889980842	7.90432057958719
H	4.58041209469608	17.24514992535597	6.74273619816910
H	5.42273371276462	18.00198992673665	8.12186155658188
C	4.05775986083458	15.05025370139116	7.99630388950601
H	4.12053233636015	15.04257862069776	6.90949841744807
C	1.72710201091134	18.00286046480050	10.04406267050321
H	1.99656711744478	17.33465592603266	9.21319024519736
C	5.44026713594845	19.34472265206423	11.83929167652318
H	6.37490549336547	19.69153797193438	12.28488720960893
C	3.02656790959751	18.48431250237800	10.67276537646820
C	6.69413997570379	17.31681443529049	10.97127342219342
H	6.44645410200406	16.45724670367068	10.33168156260152
C	3.35339249207918	10.05244486807349	12.00516585407132
H	3.25220135552587	9.12601013509093	12.57285114889772
C	4.61077632507352	10.46392100955553	11.55807102046451
H	5.48497185747490	9.84911252512670	11.77714849695591
C	3.65947231409461	12.58082073010674	7.78747061404355
H	4.43364788638556	11.83286836355485	8.01496799734930
H	3.71539475919931	12.82982365831943	6.72155922022483
H	2.69036232339063	12.09927837390910	7.98671593232968
C	3.09251942362863	19.68753175115071	11.38434213110405
H	2.19738356844687	20.30343205699891	11.48140078451025
C	2.20073419396326	12.63907246825377	15.03019275115060
H	1.32651954988375	13.27516538351025	15.22666830789186
H	2.18466394674680	11.79681865711454	15.73899353429589
H	2.11148100267941	12.22858660398678	14.01523096115657
C	0.06754872291697	12.02143864948219	9.87374800503098
H	0.51074901377232	11.59945537283043	8.96004604374544
H	-0.78307838121922	12.65512278103022	9.58169600721920
H	-0.32646500127184	11.18627250273047	10.47203401520707
C	0.87905806392041	19.14080021380865	9.46496511200424
H	0.48495801497846	19.79877787826878	10.25360122358143
H	0.01594454374025	18.72627789270318	8.92401090471621
H	1.45824600635154	19.76053640819136	8.76481573113425
C	6.86260431518189	12.92170442485565	11.44781094398909
H	6.24244648235974	13.76682813682686	11.78686461229199
H	7.81202527206533	13.32211034353791	11.06175225535332
H	7.08394304544532	12.28895464310094	12.31962832043651
C	3.17424519950906	15.78678223010920	16.79813574855100

H	3.40115104946704	16.44662833102140	17.65027954212366
H	2.09158592374228	15.58287395958780	16.83795947435403
C	2.17611180700019	17.60974211914899	14.39883444586305
H	2.46106674241880	17.95726609763532	13.39616760673194
H	1.96055685987615	18.49519115576243	15.01551883091179
H	1.25883063433685	17.01173079369599	14.31060929094376
C	7.79827259941228	18.12475806000752	10.27210959301639
H	7.45564843562781	18.51747788810144	9.30385964761894
H	8.67989647693323	17.49144036309256	10.09321123670691
H	8.11972902400023	18.97983323780084	10.88595366844179
C	0.47038964873006	13.44071133927425	11.93382248827031
H	0.13085714882524	12.65118832293422	12.62041898019788
H	-0.39801821251526	14.06399717676339	11.67396821588941
H	1.19985746814049	14.06993190160628	12.46250787166954
C	5.14325545603732	17.60831568429763	15.19031891710831
H	5.99651004770588	17.00620668554472	15.53214014829339
H	5.02121510861996	18.45851539806803	15.87932849224676
H	5.37437477512979	18.01068351202208	14.19511578469624
C	3.96930292284464	14.45442557862122	16.86984341981899
H	3.62981436582338	13.81711708659908	17.70153314355231
H	5.03751529976688	14.66092460715844	17.04779172849195
C	5.24640890082042	12.51347025912698	14.71019818418092
H	5.09237807398701	12.11371917176220	13.69815497883310
H	5.33576342902969	11.66083190538745	15.40050475837760
H	6.19084400536784	13.07438367884547	14.72438765082940
C	0.91295584412658	17.15800980919564	11.03732168790347
H	1.52421930291324	16.33202831259179	11.43518444282319
H	0.02772700380470	16.72973375513961	10.54380838855634
H	0.57254132633767	17.77135714577383	11.88431543222777
C	7.18560934174752	16.75478724912105	12.31273799258842
H	7.44964003335225	17.56317238078215	13.01089756566861
H	8.07933208400721	16.13123920724967	12.16187214870340
H	6.40668880762013	16.13465718458601	12.77767784195035

Atomic coordinates of the optimized structure of **8** (S = 3/2) using BP86

Fe	3.87962249111764	15.17030839633915	11.26052407067998
Si	4.69236874036455	13.85552811456601	15.36941454206185
Si	2.73049580226020	16.11708609206801	14.92906161412198
N	4.04008052230226	14.82070119935511	12.90526668245444
N	4.29601869302481	16.46543288855106	9.93515761178216
N	3.54301415854682	13.73069919661349	10.03271728851005
N	3.83544728047319	14.90940191953882	14.20502586862365
C	4.35587331247366	16.25498936036291	8.61014867030835
C	3.64919761002925	13.82693073319473	8.69557628421836
C	2.07242202814875	12.19816262338029	11.26031007645703
C	1.89760327875584	10.96183780935653	11.89259259759905

H	0.94475862577639	10.73182095140042	12.36902719607508
C	3.32004569918745	12.46518158993142	10.64719139406600
C	0.98159260058306	13.25685374587185	11.26609761034525
H	1.49370028483702	14.20966902407147	11.50542198688131
C	5.68883693898236	18.10008119319993	11.12010932237395
C	4.38625031986127	11.53763949223582	10.71887388976381
C	6.53948837998210	10.70899079575558	9.61915112828395
H	6.80059440179060	9.97235149085624	10.39350506289445
H	7.48004165898830	11.05443331494481	9.16619831603533
H	5.95493492825103	10.19176688316835	8.84414982006459
C	5.77083251416275	11.90026860550523	10.20157617706993
H	5.65104955244701	12.64051755685571	9.39838532210130
C	4.74359928721930	20.26175590945179	11.71469088517803
H	4.84996119059182	21.23705821748907	12.19228998012623
C	4.46846621869558	17.76407391913051	10.49423865549146
C	4.78866828327262	17.38725221108482	7.71200244340305
H	4.08454564225388	18.23149801481406	7.76439667723838
H	4.85673338492891	17.05374975510684	6.67029790202588
H	5.76883768799778	17.77606837236486	8.02667316495894
C	4.03085527814967	15.01329693049806	8.03899329475156
H	4.09344617778446	14.95569388199267	6.95336454645311
C	2.04451951270923	18.24710462963321	9.88310580873748
H	2.25263643538308	17.57661245609696	9.03589469208070
C	5.80260866950722	19.35713728268508	11.72809071378222
H	6.73872181095639	19.62947883715909	12.21961264724288
C	3.37662228022569	18.66935826327353	10.48213965073977
C	6.84731804669488	17.12074912486641	11.16415610691112
H	6.54637817626661	16.23267647382488	10.58861221243712
C	2.92624504987771	10.02007306248266	11.93771723211525
H	2.76837007162441	9.06235119368825	12.43631146164671
C	4.16273235851724	10.31672826319716	11.36648063496482
H	4.97258053615863	9.58906310405194	11.43287215702001
C	3.35483870563561	12.61377436503294	7.84203676652224
H	4.16946838670390	11.87548673070430	7.89686521697722
H	3.23126705000258	12.90660941642779	6.79283152212974
H	2.44351433274575	12.10310858778939	8.18171376566937
C	3.53995857792638	19.91449751231813	11.09645599534225
H	2.71269954287638	20.62486801899779	11.09985034964225
C	3.92804547818970	12.14704366960721	15.27145348543827
H	2.87301081591247	12.17278881201673	15.58090608040593
H	4.45719177953714	11.41947093758814	15.90447796260694
H	3.96221740781302	11.78765526538699	14.23114105611139
C	0.32032281953869	13.42958751619238	9.88877607673739
H	1.05138865577505	13.70987161062155	9.12165109164878
H	-0.44475247034478	14.21939009315864	9.93029985835277
H	-0.17004229143702	12.49477490489626	9.57659745662437

C	1.20452538771263	19.41342428739663	9.35360029409773
H	0.85756334239242	20.06949188162327	10.16570141751228
H	0.31200494471029	19.02761333750783	8.84054568427856
H	1.77224572040888	20.02727164857639	8.63943200567473
C	6.57689736323965	12.58478597632791	11.31996338853151
H	6.03916345965428	13.46282830249216	11.70721130897882
H	7.56119981561282	12.91017791028665	10.95122905498625
H	6.73290044464258	11.88792202709367	12.15763261039111
C	2.90369091858114	15.55932436881319	16.73119538022049
H	2.77691414385582	16.38824330241065	17.44526401190695
H	2.07815670935777	14.85534351050445	16.92755182639692
C	0.98312350457541	15.90415768857790	14.29243349212937
H	0.96058985118734	15.89717204028753	13.19507434780046
H	0.33768057402737	16.72597733046590	14.63721787769587
H	0.55599300448202	14.95653365294688	14.64751633151650
C	8.11162228646778	17.69902892455247	10.51159885154991
H	7.92000767714358	18.00036727971559	9.47160321931162
H	8.91876208813422	16.95152673699437	10.51026017650765
H	8.47309795947666	18.58281281455841	11.05819059292692
C	-0.08605492658157	13.02923865619947	12.33854844366408
H	-0.70514980942834	12.14711425911148	12.11392130150331
H	-0.75609597336186	13.89899351611420	12.38527309031088
H	0.36162215517636	12.88992082293965	13.33214944374032
C	3.40842033228139	17.81325881871298	14.51700117462307
H	4.39018091718339	17.97149072073824	14.98567702112324
H	2.73294521346009	18.61764926205315	14.84261078415531
H	3.54200920920370	17.90341664224145	13.42797566092828
C	4.26787100057825	14.83776774298921	16.93044739422992
H	4.26578629272383	14.21644848117558	17.83973681828795
H	5.06828510056782	15.58380928600091	17.06728255740106
C	6.52422873999959	13.80118803124735	14.99714111322346
H	6.69743853124008	13.43594461797364	13.97589378951522
H	7.04198927458247	13.12563231603534	15.69457149047035
H	6.97265642598618	14.80042816305813	15.08379494512212
C	1.25011228367763	17.42921924478215	10.91427427016975
H	1.84292479229395	16.56608909814711	11.26478196930394
H	0.31513572816284	17.04252329077488	10.48321940834279
H	1.00676615645125	18.04140366302353	11.79506742821726
C	7.11393933802504	16.66505590714350	12.60712058961652
H	7.44172979398074	17.50839333080467	13.23403167408889
H	7.90028383123575	15.89625257555931	12.63060929946744
H	6.20244568486068	16.23688321128255	13.04594237579030

Atomic coordinates of the optimized structure of **8** (*S* = 1/2) using B3LYP

Fe	3.85290792785585	15.07035581700189	11.30261103027165
Si	3.65330822974279	13.58550546457475	15.23833834829781

Si	3.63332113743985	16.59398837169778	15.22608572777022
N	3.75821327591165	15.07992644139040	12.95573417777771
N	4.08228335833172	16.42827502418382	9.97405287343153
N	3.78161927371930	13.69207957390888	9.97571635060082
N	3.67911314990739	15.08599067538351	14.26936186975268
C	4.15854771900673	16.28817824950064	8.65497872579333
C	3.85192474039675	13.81534372741944	8.65442324863575
C	2.37475858797173	11.91574286159940	10.89892549526917
C	2.28362175722276	10.71263334873422	11.59586073218811
H	1.30963225724716	10.33240221922969	11.87740921588365
C	3.65165448973614	12.39665133949223	10.56368416432964
C	1.12110018531639	12.68378992041104	10.51677175005650
H	1.42165680621011	13.46668498822529	9.82109341333572
C	5.41534124893458	18.18487573539202	11.03278907712068
C	4.81166902685097	11.65804427914833	10.85871106911484
C	7.07441219672987	11.07371622648371	9.86147278896057
H	7.33869080135607	10.31629758039889	10.60211812476670
H	8.00504673539895	11.51003147351120	9.49205726642941
H	6.57885696347625	10.57067500481694	9.02854056239518
C	6.18695978805690	12.16851871029949	10.46079395270198
H	6.04478551245990	12.92702634230938	9.69184295572878
C	4.30974477525434	20.15611319035812	11.90149970886688
H	4.36006101598738	21.09636435506652	12.43650307349422
C	4.17386427328752	17.72812517119264	10.55919535898086
C	4.38439507954222	17.50222351251971	7.79030848182737
H	3.57144668236763	18.22169937834012	7.90126805347280
H	4.45574453294346	17.21809042630252	6.74269646246292
H	5.30255266140092	18.01800034145553	8.07649539727075
C	4.03676886390296	15.04775725212187	8.02334667528359
H	4.09530478053555	15.04022581518193	6.94554618794519
C	1.66959695632891	18.01177812466371	10.17759941824437
H	1.86878584297890	17.18433443141025	9.49840874906096
C	5.45802106433898	19.40074052769937	11.71219575087687
H	6.40332100794761	19.76181811971621	12.09789154617869
C	3.00642392741197	18.49697965488837	10.71245265759864
C	6.68591602253132	17.38177659482159	10.81677952593912
H	6.43771925392982	16.54805811363718	10.16065974818149
C	3.42044578467160	9.99193080105143	11.93252721218726
H	3.33133152708681	9.06173462655619	12.47994573713637
C	4.67152344607863	10.45778498793844	11.55181775563487
H	5.55231713223035	9.88193908173046	11.80496512528549
C	3.73561483666712	12.58623746868320	7.78903419929781
H	4.54353845760700	11.88294850542860	7.99922086949392
H	3.77138610365698	12.85373793815750	6.73529633210363
H	2.80028363657385	12.05944141546147	7.98678140278460
C	3.09792693827031	19.70886937307516	11.39322244969458

H	2.20804617889880	20.30879681672396	11.53539914711856
C	2.03122118333506	12.68384374384450	15.03582596287366
H	1.17844225900085	13.34057774164747	15.21812730736496
H	1.97608965041479	11.84628063253236	15.73672952769738
H	1.94575144174518	12.28221081383864	14.02577273791324
C	0.09967692007386	11.79562255138499	9.79820830696564
H	0.54507098629245	11.29660202231583	8.93518773351291
H	-0.74167578350174	12.39768799044252	9.44772549285184
H	-0.29992328034073	11.02487629496757	10.46080531714653
C	0.93280159401712	19.09208022471785	9.37974821091584
H	0.62485261799531	19.92511300696476	10.01504007038612
H	0.03174011854518	18.67374131728611	8.92567513967654
H	1.56076881535875	19.49378018289978	8.58209020877235
C	6.88832630926474	12.85542951041958	11.63909072528529
H	6.27566370159709	13.66487541615241	12.03699560279934
H	7.84616223009921	13.27374745391595	11.32073708050228
H	7.07642318897847	12.14378820073155	12.44546620879408
C	3.20536489388255	15.80381299143139	16.88200826771057
H	3.51360646811967	16.41952447112390	17.73117548080722
H	2.11576033863297	15.71626048243471	16.94122067061570
C	2.33391050994034	17.76376243458335	14.58564180885986
H	2.61118049047405	18.15169772647794	13.60559622649958
H	2.23139909557090	18.61362177489346	15.26624867485462
H	1.36275375079094	17.27390824894592	14.49998125031385
C	7.77193622174261	18.21048397471813	10.12046470887864
H	7.40699923124168	18.63667505345806	9.18397019756886
H	8.63849319293120	17.58473359996001	9.89524876415785
H	8.11337530654706	19.03425467751078	10.75115438035386
C	0.48745012792556	13.37398557325999	11.73004695372682
H	0.14547523996565	12.63847678823322	12.46110370855864
H	-0.37365908786336	13.97155725840787	11.42188715500682
H	1.20534889585929	14.03349533746703	12.21833294030468
C	5.30193588403161	17.43272570238425	15.22483131681704
H	6.10033149612692	16.74304143418395	15.50518476090486
H	5.30490823352672	18.26767506819613	15.93090775029246
H	5.52398605779226	17.82981434400184	14.23411045172993
C	3.84918086508038	14.39286909568136	16.93034317843146
H	3.42590256380969	13.78194440677218	17.73223766612368
H	4.91987958568261	14.48104488955314	17.14049385329371
C	5.06410151890963	12.46323685055468	14.77169996566109
H	4.93003732489667	12.07468520257395	13.76225881314907
H	5.10744479512890	11.61174822317228	15.45649825608595
H	6.02040996865332	12.98719895909430	14.81479251651695
C	0.78630472190643	17.46145948937595	11.30365862233860
H	1.29837874605247	16.66479505139719	11.84441151457122
H	-0.14360414923193	17.05782471118705	10.89588960897855

H	0.53252263262176	18.24750063787114	12.01806826941038
C	7.21070797007658	16.78498031981784	12.12723238206327
H	7.49354975384256	17.57122570703574	12.83046434127344
H	8.09214843588431	16.16807357556640	11.93784517876773
H	6.45243501485887	16.16089443530798	12.60010878487278

Atomic coordinates of the optimized structure of **8** (S = 3/2) using B3LYP

Fe	3.80245126662892	15.12325048689403	11.31349469290959
Si	4.36592307010053	14.03587065549931	15.24117087177812
Si	2.18991909283582	16.10503669361701	15.26987921861011
N	3.46381158788428	15.29566288218039	13.00222048821140
N	4.31541077675817	16.47666860317779	9.95448497479784
N	3.54349161722513	13.67779385552889	9.99461903144268
N	3.35474209052154	15.16418200353560	14.30793758936130
C	4.27696074072323	16.27376263779170	8.64338842064507
C	3.58496646189635	13.83999832170555	8.67643468987005
C	2.13277614051171	11.93348064510780	10.98662658901862
C	2.04100335481355	10.69046963794822	11.61187749169789
H	1.07709261044106	10.34053676534617	11.95917254605010
C	3.39312762674708	12.37537331154332	10.55520451324305
C	0.89389841721669	12.78860071487279	10.80501158594197
H	1.16213032684051	13.61656186739779	10.14804506928240
C	6.00479386142472	17.98439058630920	10.87924949906329
C	4.54044583394765	11.57502180871596	10.72795512357016
C	6.82856417270279	10.97432523614410	9.76943200951685
H	7.12647053542586	10.28407880459373	10.56121400899862
H	7.74017367512408	11.41625292701836	9.36197750130265
H	6.34788883619334	10.39478442217277	8.97903415000791
C	5.90531654577820	12.07800403510399	10.28976478407402
H	5.75323446446496	12.78193416813119	9.47179212140722
C	5.35964675400394	20.19454708651015	11.62762848052613
H	5.62276960504950	21.14296131523718	12.07944624088892
C	4.68008134516537	17.75437985072876	10.47312637441071
C	4.65098225070156	17.40274558453767	7.71317272507344
H	3.95655161581466	18.23854619712521	7.81708423077804
H	4.64393683745470	17.07117774636694	6.67752052226117
H	5.64301890736976	17.78760888429915	7.95446610811586
C	3.90649082632551	15.05396587026984	8.05442449473705
H	3.89929238327301	15.04115620984603	6.97504390289818
C	2.25062879904658	18.48731203401946	10.23518846809217
H	2.24205351364651	17.63132369186111	9.56096776973135
C	6.32348451100471	19.21341433996789	11.45400751462013
H	7.34060914950171	19.40220529603627	11.77423847086709
C	3.69226823409652	18.74419815889292	10.63547764709319
C	7.07407566699671	16.91795448405613	10.73555196104984
H	6.62585038799101	16.06976286335674	10.21851969253470

C	3.16122645086178	9.89646184135140	11.79607861228000
H	3.07199332792198	8.93472681331570	12.28571059764682
C	4.40032708823206	10.33885620807834	11.35220816949138
H	5.26979318176506	9.71224183621028	11.49924257399483
C	3.29253750428325	12.65805340156715	7.78442850808749
H	4.05330615930416	11.88331194432290	7.90201381894231
H	3.26501341949322	12.95912102949496	6.73967458773602
H	2.33627102058881	12.20296718079043	8.04735976055903
C	4.05464288668352	19.95530892943190	11.21931985420604
H	3.30589680405576	20.72402335951879	11.36096004636173
C	3.92406407333291	12.29435079506846	14.73423854264651
H	2.88068029187583	12.06900961909311	14.96427795223308
H	4.55344089980893	11.55525339072671	15.23592593931993
H	4.05813798924420	12.17472219530681	13.65700708750185
C	-0.25234627856697	12.02231002898779	10.13674644667882
H	0.06117849165855	11.59290841175715	9.18310066429228
H	-1.09355598541869	12.69310767803863	9.94883030891216
H	-0.61447767175023	11.20775145969765	10.76749402550506
C	1.61710978832123	19.66345358796556	9.48771304209938
H	1.50423031198517	20.53886058268281	10.13044859791700
H	0.62200890949134	19.38728321240683	9.13225997259269
H	2.21796111151896	19.95652814977258	8.62436579412967
C	6.57685540363590	12.85264809650234	11.42964729440591
H	5.94837866217831	13.67745575212504	11.77257645415533
H	7.53239652864799	13.27094768421943	11.10705813708183
H	6.75565131351050	12.19917073900996	12.28648255275718
C	2.35758026345828	15.13564672565605	16.87882479907438
H	2.11503796402993	15.73109577397525	17.76309431409896
H	1.62577596262673	14.32211845056306	16.84506731343166
C	0.46096297241272	16.04511870512947	14.56907276255275
H	0.43314506808139	16.41531595265902	13.54452019438632
H	-0.20327949579332	16.67065436562403	15.17173827280325
H	0.06980431210429	15.02698814783327	14.57459361213303
C	8.26134807626325	17.39638341931238	9.89303712592854
H	7.93698275080715	17.72374330622225	8.90347284481448
H	8.98492088103426	16.58817166618249	9.76352558965232
H	8.77824825092429	18.23233572097118	10.36949045984955
C	0.45586460137518	13.39206107844419	12.14440468906435
H	0.19458062728904	12.60723370764485	12.85872979107915
H	-0.41762136072122	14.03458978579714	12.01165804011193
H	1.25725483743498	13.99191127554854	12.57392289576034
C	2.79864008980918	17.87103616397727	15.37478313427346
H	3.76394505943106	17.91952693986253	15.88379740784233
H	2.09448327455967	18.50928817330131	15.91428315042174
H	2.92870092023612	18.28016254726405	14.36992226051802
C	3.79234839408434	14.54579124189300	16.96579315763265

H	3.84711256478390	13.72839644262370	17.68985668390285
H	4.48424462919794	15.31616722456424	17.32137278569132
C	6.18998779162574	14.34908569450861	14.99492165190326
H	6.50797800629257	14.10631484448731	13.98205802333986
H	6.76717685235766	13.72680885855092	15.68481581039321
H	6.43801651177197	15.39384220030871	15.19146905558719
C	1.41971563584978	18.10261341877550	11.46598300915888
H	1.85614861788746	17.24025790136309	11.97107605427239
H	0.39431351291094	17.85568874784512	11.17950465759694
H	1.38513893398777	18.92901293237660	12.18028775932225
C	7.53111963149005	16.43041555359662	12.11467774362304
H	8.00979412673120	17.23583648493453	12.67629936095973
H	8.24668852513329	15.61091314307744	12.02005726318041
H	6.68078113422069	16.07557429423508	12.69649690962524

Atomic coordinates of the optimized structure of a benzyl radical

C	-3.41612428391307	3.37495007360100	0.06106319679206
H	-4.43193207018423	3.73205344558472	-0.04056523660373
H	-2.73226075359596	3.54366790305449	-0.75961965252887
C	-2.99784781087119	2.71331057962871	1.22267115238582
C	-3.88927698152650	2.48693468205837	2.30727080209992
C	-1.66405487290569	2.24101745222050	1.36524429239919
C	-3.47089789913529	1.83436237688735	3.44904232406656
C	-1.25798149755326	1.59023775775229	2.51241118649520
C	-2.15432812923990	1.37988127664363	3.56372002139635
H	-4.91108016087480	2.83720229860146	2.22356232794896
H	-0.96514297365438	2.40110193024670	0.55306523545352
H	-4.16810616801365	1.67344693653135	4.26213284173430
H	-0.23670627959535	1.23997966613804	2.59840865079226
H	-1.83088762093670	0.86822969505137	4.46087280556841

Atomic coordinates of the optimized structure of bibenzyl

C	-8.76575644577102	3.65543380711450	-0.06842164978842
C	-7.37255013226490	3.37804836918400	0.53512562405652
H	-9.16506896911237	2.72172635360425	-0.47161398603893
H	-8.65315118899084	4.34358981818805	-0.90954398395056
C	-9.72021734686438	4.23063550222472	0.94291445586407
C	-6.41847120689879	2.80214488222493	-0.47617160333848
H	-7.48517197573546	2.69019280106529	1.37649722799646
H	-6.97303677419237	4.31184314829261	0.93789715219554
C	-9.81152658096605	5.60958181767745	1.13614928786483
C	-10.65990400147376	6.14154722121617	2.10055848622044
C	-11.43361323709136	5.29874383034461	2.89133297139859
C	-11.35253280053274	3.92233322577982	2.70820961039184
C	-10.50240037180630	3.39604668385603	1.74228477955572
C	-6.32713367169034	1.42305094160072	-0.66833203990356

C	-5.47929732326949	0.89041300911437	-1.63283861386902
C	-4.70616942781400	1.73266788005871	-2.42478348154433
C	-4.78723299231012	3.10921289597150	-2.24270485668054
C	-5.63681631035213	3.63616582167231	-1.27667225965082
H	-9.21425111956428	6.27275990494694	0.52060170502076
H	-10.71995443755125	7.21496394193551	2.23201305092047
H	-12.09730097031241	5.71130349846576	3.64070946068746
H	-11.95496133394359	3.25793520291934	3.31544895299293
H	-10.44706276711902	2.32237228617154	1.60189067104813
H	-5.69221931436916	4.70993131270522	-1.13701258261618
H	-6.92394727163589	0.76030551765330	-0.05186778904744
H	-5.41913650406909	-0.18308551312862	-1.76357376722358
H	-4.04296058786331	1.31950975557363	-3.17425401108855
H	-4.18524798143521	3.77318925356721	-2.85084213747337

Atomic coordinates of the optimized structure of **Bn-TS** (*S* = 1)

Fe	0.16328924088922	0.26406873140511	-0.21046913783882
Si	-2.18568654253364	-2.92428740582496	1.65020516394807
Si	-0.97908801056955	-1.02781201342260	3.59496905128230
N	-0.72196104864226	-0.66784363296723	0.92625158461930
N	-1.26418757484887	-1.43224967559270	1.89034295069604
N	1.21699347977375	-0.57043476826684	-1.58999478999824
N	0.92639347885177	2.04280561569562	-0.51816960455345
C	-1.16474041714598	-4.18410227427029	0.73255400892251
H	-0.25220825605461	-4.41484946762298	1.28540263581591
H	-0.86885289771921	-3.81600646568813	-0.24892646708181
H	-1.72240752178519	-5.11341425485292	0.58909366721312
C	-3.83230942432915	-2.66759274842451	0.80097276486279
H	-4.47175370814513	-1.99800676922134	1.37969774467207
H	-4.34489055175213	-3.62995332628125	0.71255433162327
H	-3.72428817703637	-2.25258056314322	-0.19990985256794
C	0.65682842629160	-1.70569632701141	4.21009470133895
H	0.63084676354351	-2.79784121196456	4.21874870627712
H	0.85680877923567	-1.36495797985851	5.22965303560097
H	1.49167826920800	-1.39813632278485	3.58313082652053
C	-1.08863396381123	0.81960846409679	3.84376978488265
H	-0.39459397970021	1.34255762218169	3.18415426029703
H	-0.83854566992085	1.08609984788437	4.87354264429875
H	-2.09459886032988	1.18656057414940	3.63179474663851
C	-2.42873508428279	-3.35582931475458	3.47657338814071
H	-3.35824010508266	-3.90945087477721	3.63623644926607
H	-1.61767501923951	-4.01986714402917	3.79004674400979
C	-2.38759258489377	-2.04708583793962	4.31291141225846
H	-2.28300219527952	-2.25097587968178	5.38171804485242
H	-3.32253463552019	-1.49273730145099	4.18470358934383
C	-1.99744596578601	0.68522675970955	0.43053001280215

H	-2.60847602005099	0.39738128860071	1.27361752517830
H	-1.57787994780729	1.66483039250846	0.64039540818140
C	-2.76524169114757	0.64697126506172	-0.83911664270310
C	-2.19971085819829	0.94171265003335	-2.08670101069137
C	-4.13761732064690	0.37495384980378	-0.80227853015003
C	-2.97146045778351	0.98413131487043	-3.23699878739754
C	-4.91408244349741	0.40657326208422	-1.95368689900984
C	-4.33581978214241	0.71652896533741	-3.17904297623013
H	-1.13754269010067	1.13522603531040	-2.16564566894226
H	-4.60230820796717	0.15200503064619	0.14905911302263
H	-2.50201559681147	1.21711461002778	-4.18479714105437
H	-5.97464803964394	0.19593985823586	-1.89077601369561
H	-4.93811713265674	0.74387347318653	-4.07820150521428
C	1.90295230204566	0.07918140158735	-2.52699118781033
C	2.56846474372536	-0.69155581946795	-3.64304005816366
H	3.17500860029274	-1.50792962716993	-3.25094715403060
H	3.20185794131850	-0.03266748132364	-4.23241710195230
H	1.82665806983688	-1.13903776045111	-4.30458906316744
C	2.09758686874319	1.46329915791722	-2.52372976919577
H	2.70705586528675	1.86088807620998	-3.32115550577287
C	1.74165500457614	2.36355350130775	-1.51316211369563
C	2.42015640684778	3.71180783968484	-1.55486684096873
H	1.85626446092315	4.48534848754415	-1.04305780489084
H	2.59549227037149	4.01512162946686	-2.58621222954415
H	3.39377076523445	3.63879633049571	-1.06319439590304
C	1.26582596849422	-2.00177602790494	-1.59617057343579
C	2.16914531768222	-2.64414595258933	-0.72516872236338
C	2.29751841753576	-4.02855859615316	-0.80763984352748
H	2.99305348781899	-4.54071574809794	-0.15761316468551
C	1.54282140812373	-4.76940083653465	-1.70585443412739
H	1.66207112007211	-5.84445138284345	-1.75879424180061
C	0.61243084276873	-4.13079397398636	-2.50761211687137
H	-0.00805159032823	-4.71663091826274	-3.17346258124780
C	0.44792626680454	-2.74678489732126	-2.46158894635881
C	2.97989222593135	-1.84551267033575	0.28227197554259
H	2.33593779231681	-1.02955864212520	0.61732772422132
C	3.36350882780904	-2.65768885275649	1.52047532318454
H	2.49551132379670	-3.14851363762874	1.96210993873873
H	3.80082650956978	-2.00063080335940	2.27354905415734
H	4.10675201080071	-3.42399765546734	1.28963102001372
C	4.23174530979607	-1.21276882554713	-0.34042086733373
H	4.89304094589155	-1.98424696091137	-0.74277884001205
H	4.78614226738162	-0.65259201587463	0.41657717692573
H	3.97824950664795	-0.52364728244926	-1.14332238925373
C	-0.65231142526478	-2.10052853638324	-3.28219562485201
H	-0.49671797172190	-1.02304671619192	-3.26354715001368

C	-2.00727150395508	-2.38060345548132	-2.62013905667057
H	-2.20337603832682	-3.45458797651177	-2.58329113696322
H	-2.81608533257212	-1.89848874060449	-3.17039693579890
H	-2.01706499588718	-1.99508537929290	-1.60343618359054
C	-0.66814987149816	-2.54761825262109	-4.74697732841201
H	0.29934325617846	-2.39769683196377	-5.22913206428654
H	-1.41622081061553	-1.97654344648402	-5.30115756417853
H	-0.92589005427675	-3.60434018229467	-4.84219097391600
C	0.72816321454800	2.98006863885556	0.54953483074631
C	1.63559183323463	2.98453800781521	1.62608611227591
C	1.40034064075429	3.84078889872479	2.69900785351228
H	2.08308954283735	3.83915558103253	3.53931241621265
C	0.30795040139572	4.69386159188352	2.71021965723275
H	0.13846674157303	5.35159409581989	3.55369101137246
C	-0.56034278295388	4.70679928022918	1.63036713909469
H	-1.40177838776096	5.38708979608269	1.63431874774859
C	-0.37152991067649	3.86266274001711	0.53633323004721
C	2.85258208818042	2.07795958867765	1.65283688915441
H	2.93384229723685	1.59586465324908	0.68024807064752
C	2.67418472195522	0.97147650648359	2.69407959042832
H	2.55022287262377	1.38776765631007	3.69618093045280
H	3.53997280675238	0.30697172063388	2.70605114255217
H	1.79097858746586	0.37974783889569	2.46133011694651
C	4.15122878805759	2.85569216891207	1.89229881686444
H	4.27332932584333	3.66032832989672	1.16518773132827
H	5.00980885483387	2.18577515712097	1.80806344510607
H	4.17524816185017	3.30016658811844	2.88931244674933
C	-1.31904724352039	3.95064683796813	-0.64767652994848
H	-1.20680735479931	3.03319281177674	-1.21710953496018
C	-2.79149518277613	4.07177972302148	-0.23894010970877
H	-3.07097015183193	3.32206462904837	0.50057618130324
H	-3.42799580025360	3.93295397763731	-1.11446453066762
H	-3.01184701470456	5.05670745183925	0.17805254471884
C	-0.95190062641396	5.11203756596203	-1.58331361864149
H	-0.98719023358456	6.06462828876953	-1.04898876360517
H	-1.66107614398400	5.16284367819310	-2.41284749462119
H	0.04550738901091	4.99652760820473	-2.00344583976781

Atomic coordinates of the optimized structure of **Bn-TS** (S = 2)

Fe	0.16012218301986	0.21998856650244	-0.10656713141275
Si	-2.23151582667567	-3.04429119729275	1.82897211171307
Si	-1.14529626789399	-0.89199799414519	3.58195312930610
N	-0.82712382830943	-0.95260251607872	0.84167853763141
N	-1.36989371499450	-1.49774138022893	1.93071709724830
N	1.17065043821714	-0.56836114266760	-1.67547866132837
N	0.93017199623609	2.08047284718096	-0.34773692754422

C	-1.16243233672844	-4.32170327510464	0.98865944091664
H	-0.19547867293058	-4.40111055479028	1.48988094704796
H	-0.97391747096922	-4.05315302335725	-0.05127711614286
H	-1.63852915009861	-5.30564640897918	1.00395364445925
C	-3.88080359750167	-2.88349377810024	0.95952989616432
H	-4.52691724748439	-2.16965367932432	1.47523322646626
H	-4.39017290242185	-3.85120545786175	0.94446670542070
H	-3.75937478478158	-2.55169944605624	-0.07150364543865
C	0.55809749623877	-1.35406444981902	4.20736010979149
H	1.33626418635493	-0.97576044205645	3.54580942691322
H	0.65961546634084	-2.44077737685528	4.25278729578237
H	0.74041401557122	-0.95257548058983	5.20740413960214
C	-1.41226660513931	0.95286789775934	3.67397193519065
H	-0.72056143612328	1.48519794990539	3.01916659811387
H	-1.23832014967579	1.30985775920031	4.69224697018173
H	-2.42908926924355	1.22302633443321	3.38525891232271
C	-2.44200086511525	-3.33345958292977	3.68567254549019
H	-3.33702041641410	-3.91951788612163	3.91153676495548
H	-1.59099199399820	-3.92136299583433	4.04313752160628
C	-2.46389772798831	-1.95435565799729	4.40456852043348
H	-2.32440170507153	-2.05920932717192	5.48380362208895
H	-3.43654816917682	-1.47374479905151	4.25914119735384
C	-2.10434587038371	0.60303819755411	0.27137911895299
H	-2.75310090644756	0.23479492134833	1.05264066228722
H	-1.67731406483112	1.55975535099150	0.56638772136474
C	-2.74104629678272	0.60632592380369	-1.05139543886443
C	-2.09423237638135	1.08539017121992	-2.20111766158387
C	-4.05025918959459	0.12418076732128	-1.20053556062894
C	-2.72253532104023	1.07731515683993	-3.43657271066164
C	-4.68255541175260	0.11920074072908	-2.43551214990635
C	-4.02128797303055	0.59204387801542	-3.56449096033479
H	-1.08121735610691	1.45999275931704	-2.12937183165575
H	-4.57553789759835	-0.24075451037738	-0.32742641486918
H	-2.19353875324564	1.44762747157495	-4.30625697419865
H	-5.69433311198989	-0.25899763680505	-2.51811664142240
H	-4.50966026641156	0.58150509078096	-4.53054443395351
C	1.77373561313747	0.18336938043704	-2.58676414667324
C	2.44059411830081	-0.47921088382591	-3.76897274899928
H	3.24730316238722	-1.13244023072839	-3.43156871745920
H	2.85291650377383	0.26212732934908	-4.44927724247031
H	1.73620246016608	-1.10765863395698	-4.31353966674474
C	1.89259798694353	1.57844435865326	-2.50281180288960
H	2.41198626304432	2.04424196937026	-3.32734266074816
C	1.59792715883752	2.44125998286542	-1.43429427966686
C	2.13651386838823	3.84640218100410	-1.58133469653415
H	2.15224321146546	4.38132384443215	-0.63591356389180

H	1.51765652021429	4.40925943799676	-2.28187131948761
H	3.14650912871627	3.81698475114929	-1.98836945308350
C	1.32789770038721	-1.98713538561120	-1.72569626364719
C	2.37759069472254	-2.57292745834351	-0.99444202296478
C	2.51704964746824	-3.95893517804907	-1.01133471781325
H	3.32017813380923	-4.41863388748413	-0.44857565028055
C	1.64643757533819	-4.75844388130054	-1.73563791856919
H	1.76830970964193	-5.83452821328678	-1.73948026776844
C	0.61334550816809	-4.16977848827984	-2.44828267138031
H	-0.07413697437803	-4.79585494545890	-3.00312871069975
C	0.43358613586707	-2.78771380335090	-2.45586009909353
C	3.34509261759445	-1.73439457313625	-0.17985772891692
H	3.08757693214983	-0.68819694506316	-0.33451760234194
C	3.19022833146402	-2.02954360929967	1.31535420690926
H	2.16221759249447	-1.85796281705320	1.63531900766392
H	3.84754070730531	-1.38737204851981	1.90518044315547
H	3.44350146722318	-3.06770131072554	1.54090981086149
C	4.79662817358711	-1.91989995850002	-0.63601669495459
H	5.13671411713224	-2.94541612011718	-0.47672132745138
H	5.45878387924414	-1.25659048565549	-0.07455084497866
H	4.90764933696460	-1.69074744369579	-1.69739276321467
C	-0.74210960383069	-2.18953178427538	-3.20075491825630
H	-0.63047448918517	-1.10677041161776	-3.18563974391198
C	-2.04587667041153	-2.52126975017968	-2.46864341971942
H	-2.21625073044086	-3.60055464204515	-2.44972794691315
H	-2.89499363235529	-2.04486961985500	-2.95850986577992
H	-2.00009853180286	-2.16278109044092	-1.44230383524094
C	-0.81159089184655	-2.63295668917329	-4.66547093887976
H	0.11722046828570	-2.41835591282779	-5.19757090104356
H	-1.62397915141553	-2.11006659288869	-5.17513178266689
H	-1.00312287678775	-3.70477169809040	-4.75071995364756
C	0.73400318437135	2.97531964107705	0.74522686586826
C	1.50481939205778	2.77139456491209	1.91375592479316
C	1.27852109174312	3.59316054551027	3.01372237409733
H	1.85844913046359	3.45463364003556	3.91438444765099
C	0.30649107251284	4.58334059307663	2.98597460568793
H	0.13953341479288	5.20534917636067	3.85641653072412
C	-0.44990522461808	4.76520478827209	1.84303289384304
H	-1.20896874068410	5.53668019522366	1.82514423932953
C	-0.25482745533256	3.97930989233795	0.70613163815920
C	2.55917469132660	1.67538288298759	1.97206928995737
H	2.11666081914171	0.77650445355173	1.52610693392555
C	2.97988433478128	1.31094856077954	3.39616905019118
H	3.57196740672606	2.10804097856443	3.85136517900214
H	3.59881697505528	0.41332405316239	3.37902071946362
H	2.12191219610629	1.11704251470179	4.03849687282784

C	3.80709872865688	2.02029329374439	1.14688452431632
H	3.57699629764038	2.16438668031759	0.09463281665964
H	4.54024822322309	1.21428935468849	1.22156208212338
H	4.26791122274670	2.93517303856065	1.52682629904996
C	-1.09891408198490	4.27227368713052	-0.52462004081467
H	-0.83930430435548	3.54604318931432	-1.29295938451194
C	-2.60434352457207	4.14650640041696	-0.25034434343748
H	-2.86564703692487	3.17396937942529	0.15996055409392
H	-3.16478557146020	4.28021479830576	-1.17762699164814
H	-2.93689319556753	4.91143963227922	0.45438951594925
C	-0.81147142056357	5.67858934364682	-1.07548958252260
H	-1.15910576436566	6.44221524623334	-0.37642792945731
H	-1.33795587285625	5.82849121897982	-2.02071596341087
H	0.25071599252514	5.84473772910115	-1.24523724057690

Atomic coordinates of the optimized structure of **13** ($S = 1$)

Fe	6.03251610755002	13.48336973339988	4.52300520264043
Si	5.27347419494424	16.63532982825490	2.44872034688643
Si	2.69118744667861	15.66632238498371	3.46994205200100
N	5.46017421025513	11.67805236029027	4.92704644429669
N	7.92215231447083	13.05877623556342	4.61243336684440
N	5.14121277725037	15.06900919805514	4.75416086680569
N	4.43779322314766	15.66637937502799	3.64637831407248
C	4.13257507579391	11.27753633673615	4.56338333477099
C	8.38113166096997	12.06746917233005	5.37272230793247
C	8.87778514514931	13.83202924124221	3.87281747914931
C	6.23490938727836	10.78734912633951	5.54050554101984
C	3.10073898879918	11.27310708734838	5.52044808305215
C	1.57963632941139	10.43268095750161	3.83244536108715
H	0.58735099630738	10.10412943133674	3.54958807070222
C	1.83361525325657	10.84254496658805	5.13167193598534
H	1.02965406115118	10.83830983114359	5.85597913143516
C	9.45184518104732	14.99648542926152	4.40660623589683
C	3.88160434526597	10.87763174441915	3.23841518773154
C	7.57202008126774	11.03747735385818	5.86359579901438
H	8.07412265848321	10.27308856759451	6.43663834548230
C	9.23491950056703	13.38367477932096	2.58264847435964
C	10.41142129423464	15.67398818567868	3.65253519778720
H	10.86385636015092	16.57052818126863	4.05696143290986
C	4.97230263171250	10.93978318385516	2.18770424233916
H	5.92678934558286	10.93752477081340	2.71177744791709
C	3.31605623368923	11.78176582611997	6.93316430483758
H	4.38780132625815	11.82123559754060	7.11520947591623
C	8.58341297722145	12.15579203407178	1.97126510154244
H	7.59136961680749	12.07638851075741	2.41413783588470
C	2.59890413900680	10.45963768166795	2.89399600601886

H	2.39307122380296	10.15350847916941	1.87685410597827
C	9.84659151024117	12.00000798786231	5.72978065484330
H	10.48749961192529	12.23046898113247	4.88121674987651
H	10.10438654155021	11.01525758377661	6.11334800141932
H	10.06465127099047	12.73568679412723	6.50635686120684
C	10.20068131717442	14.08891748227935	1.87205726428975
H	10.48886887993715	13.75428473416608	0.88500345447301
C	5.69507200278105	9.41822367795439	5.87528405936601
H	4.87314570225841	9.46997166882911	6.58921627392902
H	6.47991462053590	8.79348809887544	6.29506794318933
H	5.30350796759227	8.93384631977884	4.97878861909956
C	10.79689595801327	15.22383313504549	2.40269955067851
H	11.54838901294572	15.75992275554159	1.83635845189321
C	4.86799952723499	12.25476023154337	1.40369942133182
H	3.90644623271116	12.31729442194352	0.88965560447987
H	5.65819211264530	12.32635060039379	0.65685052830378
H	4.94136081041451	13.12314613171769	2.06251334859588
C	8.41125100909934	12.26201762371081	0.45402818717595
H	7.91932875124539	13.19278704450554	0.16916450714847
H	7.80669336294611	11.42895367713685	0.09104489524603
H	9.37047359220714	12.21412326012628	-0.06505132926813
C	9.05067961125798	15.56809878457750	5.75194826715818
H	8.30854002070814	14.90627404544016	6.19160784457483
C	4.97766590824980	9.73880586483610	1.23943469956290
H	5.01684030262598	8.79895360156509	1.79324535312295
H	5.85135259346333	9.78529185609978	0.58614510501038
H	4.09311151790083	9.71737097774294	0.59987478535891
C	1.98070257484050	13.97146287190719	3.14671268747477
H	2.35879372009111	13.55668286875644	2.21268534518760
H	0.89114286570891	14.03243703802770	3.07477834752033
H	2.22023511449998	13.26704165936868	3.94124533319177
C	1.73945997302454	16.42920937978226	4.90238191616096
H	1.70186828191454	15.78389614684954	5.78150341554128
H	0.70734904814237	16.60116547101750	4.58010655029721
H	2.16155457714555	17.39179027079448	5.19798175075151
C	2.77794823536154	13.21298760427819	7.04932843750706
H	3.22558931410149	13.84763238757101	6.28773680882712
H	3.00815544150115	13.63689562537035	8.02861322533181
H	1.69464089104918	13.22852451026853	6.90938337267670
C	8.39946243968982	16.94258674749247	5.55888292899326
H	9.08246680825670	17.63413129943937	5.06137856449340
H	8.11932634922077	17.37314989404607	6.51954109106370
H	7.50252389917727	16.85589930231175	4.94935278656785
C	2.70556830823211	10.88064543509771	8.01006508906029
H	1.61516619834285	10.87257269562584	7.96133663364430
H	2.98685598569373	11.24508718541173	9.00024839112766

H	3.05259849453526	9.84991324587989	7.91631247833373
C	3.89869955502998	16.69966741707577	1.16531650596824
H	4.00798715317631	17.52001488218763	0.45071944417490
H	3.93421275432736	15.77022450126269	0.58787261062921
C	2.56628063272627	16.78297895271832	1.95067081825561
H	1.70349487401348	16.52608096940712	1.32894011555656
H	2.40398533822438	17.80978713074635	2.29367679040179
C	9.34047633818419	10.86509554829236	2.31633963936773
H	10.36303301033184	10.90799496014023	1.93315553508735
H	8.84334669985486	10.00420096898371	1.86291164516023
H	9.38524075935144	10.69517845288667	3.38991285719701
C	5.66677557260114	18.37236791238663	3.05477471575042
H	4.75272727511856	18.88149327467002	3.37005710725869
H	6.12324286265058	18.96365280013616	2.25552235884947
H	6.35797465620605	18.36951772917809	3.89869924665907
C	10.22917671453385	15.67284500509780	6.72724635332435
H	10.73329660652714	14.71485622377144	6.85787332138996
H	9.87263770307507	16.00371693789539	7.70463374531889
H	10.97153153897407	16.39411312024887	6.37774976322776
C	6.83144825921203	15.83853051870997	1.80269278232445
H	7.64093791841715	15.84536513690378	2.53035334568973
H	7.17517413444662	16.38802492662695	0.92140609084972
H	6.65511787068727	14.80492893158846	1.50331428269051
C	4.96213530802375	15.88955512167229	5.94875021587975
H	3.90127298333305	15.91810092284393	6.22524229770009
H	5.22706837036267	16.92631999305126	5.71249311987612
C	5.75207493124294	15.45817620630720	7.15907945817461
C	6.16969819795685	16.42932449113647	8.07086306076244
C	6.07502390226712	14.13042637170697	7.42878944232552
C	6.90685281606195	16.09027144290722	9.19772564247933
C	6.82549205263688	13.78342174952609	8.54638963449124
C	7.24921629896337	14.76258265401333	9.43554607371043
H	5.93052996007631	17.46928946831143	7.88163564857824
H	5.73834179562918	13.34449085568148	6.76895620323070
H	7.22761063378492	16.86516781614489	9.88305215758520
H	7.07769177251493	12.74376839328285	8.71259789383972
H	7.83840457077174	14.49683425393852	10.30420062011296

Atomic coordinates of the optimized structure of **13** ($S = 2$)

Fe	6.13797378668061	13.63223540977502	4.60276896848753
Si	5.35790443454206	16.71849174218984	2.43109271669008
Si	2.73673357485353	15.77195614346426	3.39577320595922
N	5.51660083378483	11.74411158206683	5.02806794287325
N	8.09659144484451	13.08202792710625	4.48870058458965
N	5.09011324530890	15.21300815497719	4.77869122927734
N	4.47303068176241	15.78529802611397	3.61206408484545

C	4.18079768563690	11.33440165102236	4.72958841519780
C	8.53249675715358	12.07070187845148	5.24400658209813
C	9.05186124412882	13.80558959486365	3.70593207207443
C	6.33407032285072	10.86317923964913	5.58354453461096
C	3.16588172931329	11.46179942219435	5.69156231917953
C	1.60688326601446	10.45742531481639	4.13114805259615
H	0.60739290949024	10.10927338619652	3.90225185543435
C	1.88730740768836	11.00485657999969	5.37202177878602
H	1.09661054070663	11.08919448912429	6.10685146783595
C	9.73779165244064	14.91312507643615	4.23121302550007
C	3.90084693478633	10.81393970407734	3.45217178524468
C	7.70342237129942	11.09695431217372	5.80885517740165
H	8.21355429531793	10.32998241981820	6.37109353610909
C	9.26995463732801	13.39318304950495	2.37395315023482
C	10.63118944405735	15.60073074773096	3.41021695251331
H	11.15505089689358	16.46277585714655	3.80240456004294
C	4.96580327787843	10.80498903993312	2.37257275906215
H	5.93707104109619	10.81136273891885	2.86641384542443
C	3.40761387096557	12.11651223861902	7.03896453536249
H	4.45090147144671	12.42328479669176	7.08211914024925
C	8.52048742647328	12.20647647391575	1.79273348866520
H	7.54623850076740	12.18700208439982	2.28140470358714
C	2.60873768232489	10.37882408795403	3.17456862723046
H	2.37841502735965	9.97860733645524	2.19608160510049
C	10.01413929605325	11.92072607345543	5.50487161447893
H	10.59366662754621	12.01314823301396	4.58770300505882
H	10.23075232086897	10.96000768716636	5.96576418101343
H	10.35775658197498	12.70995305299120	6.17660591983016
C	10.18486528219084	14.09914550211217	1.59853860278948
H	10.36447022749080	13.79706016895506	0.57620685405933
C	5.82901777429488	9.48683799077684	5.94604725694323
H	4.87642691707123	9.52366670571749	6.46925169487575
H	6.55604492750671	8.95901995495465	6.55898934893954
H	5.66529237280432	8.90694874010918	5.03367594860099
C	10.86160401778449	15.19889237944355	2.10581628849831
H	11.56242617123240	15.74120151110356	1.48335219113587
C	4.85233499897504	12.09062879206244	1.54140254917312
H	3.87373484618016	12.14694572195364	1.06106743478041
H	5.61582592286387	12.12611692638322	0.76552567417702
H	4.95589442861288	12.98355401189081	2.16307758885927
C	8.28800406920639	12.31616275967358	0.28453258244778
H	7.82296375003422	13.26520522570899	0.01487156485996
H	7.63467207700104	11.50751057248491	-0.04722330962012
H	9.22097187197941	12.22421369698769	-0.27503453312524
C	9.50330244588562	15.40394513108872	5.64583304107482
H	8.97486981704740	14.62002974438129	6.18359587026109

C	4.92720581894943	9.56978240756430	1.47133283662948
H	4.97088300941390	8.65087310015550	2.05891624250996
H	5.78169560523884	9.57994460006299	0.79138965280726
H	4.02386853180490	9.53677673665727	0.85916486858846
C	2.05194097635910	14.06500269811751	3.05823813005045
H	2.41858331520425	13.67668141536098	2.10762779294418
H	0.95984288510156	14.09554560580767	3.01238876034596
H	2.33039269375936	13.35520542162451	3.83722346104261
C	1.76342165213407	16.51542364967800	4.82370854086623
H	1.82325426470694	15.91670148374798	5.73326590431131
H	0.70797722452154	16.57598909939864	4.54029732495532
H	2.10694848838829	17.52627884726532	5.05314509601409
C	2.55034342280580	13.38077259267411	7.16412526172865
H	2.80139751870529	14.08505741514533	6.37304909474099
H	2.72665198492334	13.86957360498578	8.12180970141506
H	1.48676451879672	13.14464325532863	7.08905525074404
C	8.60398651446577	16.64455800535700	5.62916974045824
H	9.11078378283056	17.47927914845552	5.13976289450189
H	8.33921688107804	16.94804037782241	6.64382080536425
H	7.68427395039303	16.44514339157887	5.08148756586693
C	3.14895258361431	11.16929498194562	8.21617974775067
H	2.10437560503773	10.85091086518725	8.24714545329300
H	3.37387864946954	11.67460217579532	9.15785481389604
H	3.76936494321467	10.27376222499504	8.15925008087843
C	3.99312747704553	16.86571873114132	1.13822013845993
H	4.12043939786550	17.72241562507089	0.47065366546242
H	4.03788576094507	15.97126727311745	0.50803946301305
C	2.63683580325214	16.91132393670601	1.88947953927665
H	1.79724298486407	16.66460829272689	1.23316205694441
H	2.45396413491050	17.92737776354207	2.25413462308350
C	9.21280818635372	10.87248356644788	2.10970937876374
H	10.22441097482773	10.85961739831342	1.69664922133948
H	8.65439503853784	10.04468200986625	1.66620374831722
H	9.27872359983105	10.69133400940453	3.18009739893096
C	5.89882561564334	18.40127025387054	3.07199956787045
H	5.04496106085586	18.96498524811377	3.45523907504204
H	6.36354093529936	18.98934695100538	2.27531076832810
H	6.62676572576752	18.30332026889077	3.87995173074959
C	10.80040464924228	15.68019461177177	6.41268064802203
H	11.46035935901174	14.81069496910774	6.40835957684234
H	10.57212229005889	15.93085872692761	7.45076494386432
H	11.35018605234596	16.52039757671845	5.98491707133969
C	6.86107285195717	15.82487740325193	1.77501690339683
H	7.64938778239032	15.72571016494301	2.52018237063463
H	7.28255554043393	16.37939218455999	0.93162138288194
H	6.60305862170885	14.82615249576136	1.41912168295833

C	5.06589276068847	16.13953719752389	5.90334434323218
H	4.07172743331454	16.58318325143925	5.99186875150746
H	5.75099547060391	16.98833589442782	5.74554556754041
C	5.39500535372388	15.48478062125028	7.22109371315525
C	4.67516113315645	15.82182777206543	8.36725241347357
C	6.40021331854110	14.52804854947517	7.34663909379129
C	4.93525559163574	15.20794478259746	9.58643056689645
C	6.65586765947653	13.89190133743996	8.55502548450982
C	5.91880589650432	14.22875750601307	9.68364931402714
H	3.88386444971729	16.55867186545225	8.29629438944769
H	7.03821283228022	14.29151527798852	6.50400346906157
H	4.35516329433141	15.48116283643888	10.45926564904566
H	7.43085732271727	13.13790283408910	8.60700444068808
H	6.11009785743896	13.73745723261699	10.62918658701916

Atomic coordinates of the optimized structure of **7c** ($S = 1$)

Fe	6.66519842035352	7.14784358013293	14.96704622318167
Si	4.47323581419303	4.77147599710602	17.25222255948820
Si	5.99794882476972	6.47663183389594	19.20736589993214
N	6.36885703834250	6.68249158176674	16.56374394884082
N	5.68064231258405	6.06447679234282	17.49744387130745
N	7.69302645484563	6.39081928843715	13.42710726105848
N	7.27841914846577	9.00652987363303	14.60840041779058
C	8.15014190992583	10.86604286502063	13.26582461778148
H	8.96156928650235	11.20251437325541	13.91376456860369
H	7.27899278639195	11.47398019497098	13.50948549645825
H	8.43838458998443	11.05298852899826	12.23350748957441
C	7.86384862641173	9.39856895736852	13.48102727681225
C	8.30020814795762	8.51625849080530	12.48762230264711
H	8.79254484356453	8.97079249343991	11.64005841505076
C	8.33944704196290	7.11139212771635	12.52590239536663
C	9.24620747117116	6.45567151980875	11.50890615321316
H	9.03977867806461	5.39794882975030	11.37954645801590
H	10.28287063145876	6.55414549621484	11.84125469697698
H	9.16385790436995	6.95887983632191	10.54584617032917
C	7.20073860485584	9.92012572322712	15.70263360315593
C	8.34441553321393	10.10197531670036	16.50732527990969
C	8.27256042893014	10.97938577915824	17.58602782952620
H	9.14364725232983	11.12094698790690	18.21336992856450
C	7.10400786375104	11.66853443954959	17.87568447476179
H	7.06638462229220	12.34998810576730	18.71651569417253
C	5.98099585908022	11.46467691531877	17.09066957712268
H	5.06484050456399	11.99211317479583	17.32560977561524
C	6.00382713103844	10.58935046450010	16.00551373721598
C	9.62749178944313	9.32670888916164	16.26025744166839
H	9.53382522097069	8.81567841260871	15.30398041335713

C	9.80796200104868	8.24718696972198	17.33476236007508
H	9.90508363204091	8.69683413002322	18.32584093380770
H	10.70609659685781	7.65663915220719	17.14000963054781
H	8.95114266503132	7.57474449672463	17.34780365713943
C	10.86213495542748	10.23048469697054	16.17878107506654
H	10.74319373747608	11.00085240936427	15.41467887588558
H	11.74489475134998	9.63804912249912	15.92802455243127
H	11.05897847897117	10.73036547044365	17.12945649295569
C	4.74187237860876	10.37579248578391	15.19416647814140
H	4.96878981735148	9.63782262953620	14.42846105298761
C	3.61836519615974	9.81076771849928	16.07330185533317
H	3.30722526670492	10.53652224028620	16.82811743883936
H	3.94499639472668	8.90721534688396	16.58881465790376
H	2.74443286555318	9.56279254433510	15.46688073459335
C	4.28845845902753	11.65940888458679	14.48821242510347
H	3.40085083727047	11.46677735823450	13.88091959328995
H	5.06959658255448	12.04808075748405	13.83272264062546
H	4.03854087759218	12.44031621043322	15.20993933702534
C	7.76361379712685	4.96596965648801	13.41233316413581
C	8.65565098627277	4.31248261268449	14.28281575416864
C	8.70697111116375	2.92055027173432	14.26913053520870
H	9.39794131428766	2.40959425460047	14.92769043833682
C	7.89328151384572	2.17885718016415	13.42727764384076
H	7.94890187005335	1.09735183058300	13.42713393895731
C	7.00067626090260	2.83227637149809	12.59243490336742
H	6.35728072122884	2.25066390182063	11.94459049916291
C	6.90993725911251	4.22291986788231	12.57139694223594
C	9.56516796255754	5.08095147213177	15.22240198772172
H	9.28788255970331	6.13276635611849	15.16606970448838
C	9.37255399021665	4.63200753245906	16.67400040381720
H	8.33216546575074	4.74823356317567	16.97567688250823
H	9.98845013105171	5.23292045852243	17.34426341930181
H	9.65557980011033	3.58613246065883	16.80948230236017
C	11.03395040734214	4.97098681936359	14.79536118171510
H	11.37936693926231	3.93557331545007	14.84142962326395
H	11.66820364499624	5.56883863570327	15.45411709055756
H	11.17471272784952	5.32524296538319	13.77288673030417
C	5.90545478968019	4.88410608061216	11.64271723133637
H	5.86515426861960	5.94021469611062	11.90174402013625
C	4.49657744682795	4.30019596960946	11.81650962370229
H	4.44075577891991	3.27918019572067	11.43334416542438
H	3.77019962418098	4.90080797220512	11.26517452937296
H	4.19628860300870	4.27978451124889	12.86354255139699
C	6.32707675641158	4.77644532208576	10.16997700387823
H	7.29346046222165	5.24382184041913	9.98731590999651
H	5.58904350228694	5.26511245723482	9.52944053661034

H	6.39700771734047	3.72978242600392	9.86414986924331
C	4.38207081608642	4.22411547451989	19.05403904627986
H	3.44329818716858	3.71234931168395	19.28199718763269
H	5.18440928401801	3.50469609828635	19.24463977992264
C	4.57952365955012	5.48097364574032	19.94497188303873
H	4.75894287982824	5.21121296478764	20.98901109841053
H	3.67367451253554	6.09468766823857	19.93199869627671
C	5.16941003800294	3.50857365334181	16.07160412298443
H	4.44566589842948	2.71348420621884	15.87675258285178
H	6.07650101244682	3.05649986819849	16.47654809438680
H	5.42900146549850	3.96753197294432	15.11691945067318
C	2.81250435828419	5.41645610165259	16.68289993187492
H	2.48249860020486	6.24786859754595	17.30956453569254
H	2.06735685058906	4.61917361774391	16.76064137281111
H	2.83875850957185	5.75842995482906	15.64968097048297
C	7.66132294274203	5.84040847947017	19.77496795012799
H	7.73722796207584	4.75926257925179	19.64441177910544
H	7.80274863372344	6.06610558137368	20.83555903767914
H	8.47526776396982	6.30764074715666	19.22026021515137
C	5.88721016313909	8.32529572095052	19.40276386845067
H	6.62708962611073	8.82008766485957	18.77059612652140
H	6.07044460958051	8.62723037899529	20.43692113878836
H	4.90186407631777	8.69015551006753	19.10695599635179
C	4.82407702653438	6.99657578058860	14.24527265765266
H	4.57019224446117	5.95660057990857	14.05590648512995
H	4.07404771865656	7.45342138893374	14.88213657768242
H	4.89232451259205	7.53516196133438	13.29702305822238

Atomic coordinates of the optimized structure of **7c** ($S = 2$)

Fe	6.34116839155840	7.21613008153240	14.82426453038604
Si	5.04992858328421	4.24510676879019	17.38541433675381
Si	5.37310811260638	6.77321031705440	18.97912458077023
N	6.26497414113869	6.47796510560010	16.40941799917472
N	5.72729290068629	5.90719543927021	17.44588681132441
N	7.53979069054381	6.36989747052122	13.41009128242505
N	7.36270020183934	8.99713782154239	14.75332739360805
C	8.60440409274951	10.75159714747772	13.54249613675162
H	8.35062205526999	11.39062281844370	14.38239584661772
H	8.26182201231832	11.22237747434705	12.61976517242087
H	9.69159222102538	10.67526980233373	13.48323578061167
C	8.00306280346914	9.36935762045441	13.66190170446155
C	8.23292744210842	8.52198804832302	12.56185943223485
H	8.71766285403608	8.99347327651315	11.71944539944338
C	8.11467467219205	7.12859242015383	12.48109824025027
C	8.72634095541161	6.48078400070075	11.26064442701147
H	9.34979812879109	5.63078560682489	11.53559284654617

H	9.32682707487737	7.19540833831342	10.70294463911792
H	7.94438574665846	6.10038113615738	10.60370599427289
C	7.33196465837200	9.81893915871552	15.91494376766538
C	8.37889755674265	9.71342496797170	16.84902593566016
C	8.29955038965259	10.45333770163230	18.02634361698587
H	9.09821427855397	10.38038084231266	18.75432810409432
C	7.22064282910339	11.28752948215121	18.27874951219257
H	7.17477760924989	11.85572274013987	19.19951117960203
C	6.20696408825660	11.39959658271227	17.33920717174106
H	5.37412695986255	12.06313648329108	17.53346282087770
C	6.23750412188980	10.67206536439511	16.15041992277377
C	9.59965630697797	8.85068609865023	16.58297196703276
H	9.45231555995614	8.35100639338458	15.62735387491048
C	9.77777386403002	7.76101504954188	17.64261022118961
H	9.88158437763028	8.18891961151473	18.64162780426353
H	10.67839752462731	7.18088020335044	17.43543409563942
H	8.92630589507747	7.08107447123913	17.64874402263997
C	10.86722258191121	9.70794279826112	16.46759227896933
H	10.75664343325352	10.47980976233594	15.70459777165020
H	11.72356169559416	9.08406802129825	16.20141797061213
H	11.09481523945679	10.20372563830045	17.41404675658016
C	5.13675678063335	10.85702413740897	15.12076034332695
H	5.23339087478260	10.05545475575904	14.39102816098242
C	3.73195132152506	10.76371386170267	15.72409504721354
H	3.52996743247636	11.59367547552212	16.40466353547586
H	3.59789721679139	9.83532042081362	16.27630458398068
H	2.98328515437455	10.79749714743894	14.93008385949080
C	5.30203411537948	12.18986845974268	14.37548081223116
H	4.52912476129372	12.29627609426032	13.61058084122963
H	6.27331421406339	12.26052199281601	13.88709752189091
H	5.21259854352935	13.03175950518071	15.06648741867025
C	7.67619275953822	4.95177049530928	13.33447162924523
C	8.70561068815451	4.33614173509793	14.07859863582234
C	8.88393655848561	2.96114721927924	13.95701672111668
H	9.67594904562886	2.47441295980150	14.50878328814539
C	8.05123782864884	2.19430761657356	13.15419595797918
H	8.20687141507826	1.12566285602647	13.07273970227204
C	7.00452120819749	2.80054724700488	12.48161079900066
H	6.33508454304008	2.19548684374554	11.88304233308263
C	6.79326359948453	4.17762366060684	12.56026972627657
C	9.59540382489726	5.15288916563035	15.00069896758314
H	8.98322233336028	5.97316742206574	15.37765709514897
C	10.09013485948835	4.35495704143378	16.20984344672144
H	9.26317536715286	3.88379859863041	16.74187982807943
H	10.60620830863936	5.01760881828464	16.90432248527476
H	10.79743488748418	3.57468538754891	15.92130856053452

C	10.78904517466034	5.77024075841661	14.25892847145636
H	11.40428411797711	4.98962219047106	13.80420964262724
H	11.41596474431951	6.33192470264671	14.95542148902360
H	10.47082113883122	6.45465628928248	13.47440826289300
C	5.59496997770504	4.77796548334883	11.84732232088382
H	5.61816659571618	5.85583328168709	12.00333556311786
C	4.29428913920610	4.24011897220721	12.45950069525733
H	4.18309089355868	3.17279112469396	12.25596775932909
H	3.42810227927548	4.75718544455573	12.04162977620426
H	4.28582289467911	4.37511786492821	13.53944442556030
C	5.60198041340759	4.51405401121215	10.33602450292919
H	6.51762091105579	4.86795681762962	9.86192425822195
H	4.75841367568377	5.02093477817632	9.86222644577211
H	5.51009620805512	3.44702799143584	10.12212761506223
C	4.85226493477691	4.01990182992355	19.24152946843617
H	4.15446365793190	3.22119040587656	19.50531517064182
H	5.82348870471614	3.74024196437186	19.66168763111342
C	4.39869093441005	5.38997255005774	19.81951516393127
H	4.50563899046403	5.42141130504391	20.90707441396792
H	3.33565378519795	5.54313305717199	19.61041061714721
C	6.22121720372620	3.08437339266965	16.52947339305306
H	5.79269076501051	2.08078936673287	16.47336410907595
H	7.17326030068574	3.02083665327200	17.05776626105426
H	6.42340064371122	3.42053797188139	15.51178209954565
C	3.39232230571719	4.31369018636074	16.52235235306476
H	2.68504200840036	4.92288057250479	17.08903657424151
H	2.96754638987953	3.31322760430459	16.40793471622329
H	3.49160270554102	4.75553492022772	15.53055195530882
C	6.93729510891905	7.21570547507193	19.89469157117979
H	7.59613870580945	6.35272735822594	20.00779977169822
H	6.68454690328067	7.58653779097728	20.89196291026192
H	7.48223580906522	8.00189283496969	19.37303287283479
C	4.37457417066503	8.29919991067625	18.60883350338900
H	4.97347727711817	9.01119430777701	18.04022839703944
H	4.05644051794006	8.79127457471790	19.53143898870424
H	3.48546300817997	8.05210058644134	18.02580085277587
C	4.40711307546466	7.45681256973731	14.21033312742730
H	3.93068456074344	6.49622813023380	14.01556023390176
H	3.81735221426485	7.98416929589875	14.96124556931240
H	4.40342846135585	8.04050469094227	13.28379439628709

Atomic coordinates of the optimized structure of **10** (η^1, $S = 2$)

Fe	4.87319766068948	3.83278464701006	13.32958876258251
C	3.27675438929291	0.97677535740938	14.66987750085420
H	2.28969095501170	0.99011726551296	15.15167713057926
H	3.73885542171427	0.01016825268622	14.87055098048354

H	3.12867591486229	1.05575698555349	13.59238014828949
N	3.73123468977468	3.38188425675007	14.74961057215787
N	5.55834176143919	5.56580211560430	12.59789140320624
N	5.71884819679512	2.72340346094450	11.87064197248872
C	7.05306791499877	7.00353614814665	11.28841857038503
H	6.25820802645581	7.71582176763717	11.06748278796414
H	7.71295397776753	6.93001477964060	10.42749019171026
H	7.61800331187084	7.41416457336708	12.12696568856770
N	4.15542225254598	2.03803212487553	15.13002307248985
C	6.48980474983485	5.65056981539876	11.65140146627337
C	6.97625366004264	4.55457411381879	10.93153236057205
H	7.70834005246752	4.78569101551042	10.17290058506357
C	6.52911654730757	3.22294549315041	10.94895782343975
C	6.96149302954121	2.36995964135844	9.77941736891052
H	7.05035794730376	1.31872702425819	10.03902452396651
H	7.90739446912762	2.72649238774535	9.37575594784113
H	6.21174629467054	2.44566535285306	8.98758453215435
C	4.96806314705713	6.76246810643601	13.10295678162123
C	5.56284642367376	7.45753371952070	14.16808461623892
C	4.96673263734623	8.63825348368205	14.60695390277449
H	5.41277317784549	9.18630166215617	15.42680399624983
C	3.81012522435224	9.11966437488833	14.01511430744345
H	3.36592502694617	10.04466550927841	14.36102265678830
C	3.20419616464248	8.39606848294055	12.99779608898580
H	2.27950973554495	8.75893864236870	12.57042418387993
C	3.75226322490405	7.20072970436038	12.53943551753202
C	6.78890900302565	6.91637200400488	14.88030181796741
H	7.21100887474904	6.12581400678374	14.26019154219028
C	6.37512687401606	6.28130918396110	16.21451548944415
H	7.24143513404679	5.85540112039696	16.72495401991591
H	5.64632765501124	5.48629272150744	16.05789082769860
H	5.92495823431805	7.02814491013526	16.87260469354265
C	7.87601856598936	7.97415341065854	15.09403790191308
H	7.54651025622416	8.75487061484044	15.78236585992910
H	8.15760484121649	8.45362248831220	14.15470700806448
H	8.76841321621567	7.51325927870283	15.52266141777176
C	3.04258024064490	6.37578462298838	11.47773046987723
H	3.31038827950990	5.33188430902765	11.65597355495907
C	1.51815619339410	6.47246280090285	11.56915195794374
H	1.16453632560563	6.25010425942169	12.57702761224316
H	1.06073648221732	5.75734279669086	10.88394868475025
H	1.15506901493589	7.46442713096546	11.29315106494446
C	3.51907943938933	6.72059895464075	10.06079495021080
H	3.32262875780392	7.77129096497659	9.83320085402824
H	2.99181891418077	6.10921882905946	9.32450473448536
H	4.58628884461948	6.54033532163739	9.94077764196532

C	5.20974347800319	1.39946392375581	11.71223605465937
C	5.82402788995143	0.33271684426252	12.39520760996547
C	5.29492718648393	-0.94465861500522	12.22892040245222
H	5.75057705814402	-1.78261439848479	12.73733569241119
C	4.17739782054474	-1.16569104514257	11.43567652839974
H	3.78016611810566	-2.16721252931487	11.32632630039967
C	3.56480617477545	-0.09977582157673	10.79787198595021
H	2.68076337865845	-0.27301590234501	10.19772137784736
C	4.06645981149261	1.19510025824270	10.92081926094891
C	7.02079941142790	0.57069671241853	13.30134288071575
H	6.86341695564359	1.54212350073196	13.77408894507677
C	8.34714928953177	0.64357541880702	12.53035354997365
H	8.50652629459654	-0.26948822291615	11.95139179232305
H	9.17907989758164	0.74890566628535	13.23055047492366
H	8.38091888582300	1.49001643716111	11.84843643590308
C	7.13989915282995	-0.47230194700878	14.41298745144111
H	6.19939417852953	-0.59527826717301	14.94755713266556
H	7.90262443992228	-0.16501627502410	15.12988218679422
H	7.43885372447625	-1.44698844587472	14.02136253104542
C	3.34297329364895	2.34871738776172	10.24852639373353
H	3.98233761622778	3.22815815594698	10.30916573377384
C	3.06528410988393	2.08815870416504	8.76447820895650
H	3.98111281668707	1.83199542512219	8.22862997855538
H	2.63692331478612	2.97943768982866	8.30104776043478
H	2.35633431961832	1.26989279112318	8.62360886037422
C	2.04644240003160	2.67443138524691	11.00155403396754
H	1.36827767846652	1.81801934590415	10.99532017983213
H	1.53315666333106	3.51723557268911	10.53513432319167
H	2.25159764804378	2.93194491071731	12.04136899685278
Si	2.37057683128681	4.03740647514592	15.61214609765584
Si	4.91758221823543	2.05583876690651	16.70935706482739
C	0.70923713645992	3.63298026584563	14.82140589933167
H	-0.11372147270025	4.05845581029977	15.40350360882665
H	0.54801862119529	2.55618116587045	14.74395302614241
H	0.65489821468798	4.05077791209575	13.81280963737733
C	2.50840261623131	5.90197473674726	15.70425688752174
H	2.60409573087616	6.36231638898695	14.72181870893344
H	3.36951253913906	6.21436633641626	16.29536341431580
H	1.60890737359893	6.30959742072416	16.17417369061989
C	2.47534690801433	3.31480540548572	17.35362936365431
H	1.87318325112937	3.92415195094029	18.03617173509430
H	2.02888567074320	2.31491579804470	17.37124588874109
C	3.95144178631761	3.25263212158819	17.80424546101649
H	4.03926626203335	2.93272662469626	18.84874313654079
H	4.40195670382202	4.24818332431005	17.75484204966499
C	6.70862473219610	2.58559221221408	16.55681794481281

H	6.80269670505195	3.42381051345916	15.86538013330455
H	7.33243556824026	1.77068343612088	16.18885405749072
H	7.10271660602230	2.90447353023430	17.52556506659531
C	4.80663686642327	0.34419538159599	17.47663971990563
H	5.31878431955743	0.35013823232411	18.44284299520900
H	5.26512000112553	-0.43308207843729	16.86496771396109
H	3.76537067012410	0.06549961360264	17.65551608954614

Atomic coordinates of the optimized structure of **10** (η^2, $S = 2$)

Fe	5.15434968251649	3.75170562562079	13.49068195161864
N	5.58462222455378	5.54345392206986	12.64827688856025
N	5.76722373515064	2.77091078604238	11.80622266159649
C	6.93650109095302	7.07501358961140	11.27684642325277
H	6.09868245004920	7.74787440394947	11.09486906378659
H	7.55704337796053	7.03675923672959	10.38482717351407
H	7.52027366737614	7.51388917795007	12.08718507844491
C	6.46037849160359	5.69008824592558	11.65363185122846
C	6.97143444372938	4.63426898820826	10.89049473834064
H	7.67768020728837	4.91184600182819	10.12325223363161
C	6.57525518796400	3.28963926597589	10.89461207571074
C	7.07961067395013	2.42379278336918	9.76580858483454
H	7.58533470471495	1.53685927623444	10.14715689398252
H	7.76789154498238	2.97664630422795	9.13083976547741
H	6.24747432723185	2.07246218398830	9.15323462333962
C	4.98142100788878	6.72433621527957	13.17640524674444
C	5.61518007331977	7.45512889982234	14.19337996629114
C	5.00519156286404	8.61949292032945	14.65703289466236
H	5.47938078867030	9.19215783547995	15.44355749801823
C	3.80501973022155	9.05987745296811	14.12292980510773
H	3.35102439755225	9.97427692142449	14.48421206531178
C	3.17581132183810	8.31451492329652	13.13559887698419
H	2.22427312704703	8.65136156029232	12.74791477669565
C	3.73407172214596	7.13042223436599	12.66094937221342
C	6.91358022036212	6.97737282757742	14.81597444845327
H	7.34496796623073	6.22999028403576	14.15034038952051
C	6.62739237484856	6.28305744339605	16.15200212043404
H	7.55170363492853	5.90848954997978	16.59687499380752
H	5.95052632209042	5.44012102067995	16.01282478377101
H	6.16539604701483	6.97750534372897	16.85797761197965
C	7.94673626891086	8.09428937979240	14.99241048571013
H	7.62476884433015	8.82707265516436	15.73493473317635
H	8.12830693605676	8.62474628628719	14.05582942930505
H	8.89469136304783	7.67473680322047	15.33563989424782
C	2.99948181229830	6.27561915053292	11.64015595855327
H	3.26171763476247	5.23844208670061	11.85739156778575
C	1.47733549677183	6.39712688940022	11.74433585488028

H	1.12868716815663	6.23286992926951	12.76453040072136
H	1.00263867121889	5.65530136257111	11.10132064512248
H	1.12606169370696	7.37927398806440	11.42144846463316
C	3.45047202771789	6.55593411354526	10.20011528141326
H	3.27431842440906	7.60208187270314	9.93762717606513
H	2.88754530307399	5.93186510279274	9.50183080083579
H	4.50740833880901	6.33958007186730	10.05834849616654
C	5.28086676654171	1.44283802536628	11.64124453213778
C	5.97601055105616	0.36181061623140	12.21229209005387
C	5.38975598467371	-0.90152417799662	12.17054729595329
H	5.90255982208379	-1.74068867052783	12.62380628008926
C	4.16422414372967	-1.10410544456120	11.55266869990274
H	3.72052928040822	-2.09178985167049	11.53519022127203
C	3.52097214071247	-0.03971274110435	10.93760186191540
H	2.57787520105831	-0.20787513295993	10.43237559621988
C	4.06459340945715	1.24197024463004	10.96252832097415
C	7.34123791892297	0.54526553854944	12.85158927593875
H	7.66203347132929	1.56685793241798	12.64979642355398
C	8.38776720448251	-0.39954529250893	12.24797534730834
H	8.16261647732284	-1.44260003947451	12.47961879793149
H	9.37603117925414	-0.17543821278235	12.65557831480879
H	8.43798377479335	-0.30303204592456	11.16185890345082
C	7.27368826795035	0.38399183106512	14.37163921929980
H	6.55932566768342	1.08741894487323	14.79285243447191
H	8.25150915014168	0.56738039817833	14.82327351218266
H	6.95966606570363	-0.62626770094361	14.64434553426138
C	3.34988122529809	2.38132269720652	10.25495378826680
H	3.99024167220348	3.26027124496714	10.31212397607729
C	3.12771042390792	2.06915778269573	8.76941669342577
H	4.06354482206965	1.80367182218651	8.27469390967244
H	2.70805370534178	2.93930758688079	8.25984676077908
H	2.43296077427964	1.23715813795112	8.63587017098481
C	2.02393935237469	2.74141859316354	10.93363386966802
H	1.35799885510568	1.87712233632974	10.98333916199518
H	1.51385806057303	3.52735649841940	10.37377219595874
H	2.18855489763857	3.10286840459227	11.94758350777883
Si	2.15846489698765	3.85857447376433	15.50724602405912
Si	4.88280939743992	2.40684874878506	16.79468300785411
C	0.48250818992810	3.67287888208085	14.69160045773390
H	-0.25607198983829	4.28846885662160	15.21149311916918
H	0.13135595746972	2.63959550539968	14.71200236456573
H	0.51660491141517	4.00024679513286	13.65045867433133
C	2.64175398932727	5.65140600565297	15.62054740501129
H	2.58071677325607	6.16677143527906	14.66581734026229
H	3.65400887958158	5.77701851965735	16.00029910789019
H	1.95843752631694	6.14925885776243	16.31462491179456

C	2.17366918983406	3.13383492250616	17.24566172756577
H	1.42409743263225	3.65273540585588	17.85430890499451
H	1.84895994503248	2.08899685244243	17.20257981744389
C	3.57936377461338	3.22790393851055	17.88975643032812
H	3.58084770003312	2.77619916713752	18.88820020710619
H	3.85360774311395	4.27855132038146	18.02722056140715
C	6.62296985939829	2.82978718762054	17.34868632344140
H	6.86584510494651	2.27955111568515	18.26131185342406
H	7.35946301513581	2.55723149390261	16.59010268235907
H	6.73210005218611	3.89318958206703	17.55728133894743
C	4.63118581649243	0.54629784891228	17.01158371594714
H	4.98161478494624	0.25122651135794	18.00555236176923
H	5.17909864605345	-0.03956599405992	16.27384166606143
H	3.57752991580451	0.27010500039785	16.94238195360134
N	4.67677817444962	2.99055945247774	15.17733749138145
N	3.33436922258121	2.92490563966033	14.54879240322663
C	2.92427998633801	1.55274238603245	14.21631700523270
H	2.10627049675698	1.57740689262876	13.49930704155032
H	2.59421274240467	0.98973855899207	15.09509784105686
H	3.75969150894610	1.02197039580444	13.76775551821763

Atomic coordinates of the optimized structure of **10** (η^2, $S = 1$)

Fe	4.95596774754971	3.67793984728505	13.41297810783837
C	3.15474731448115	1.46069409103625	14.38455058715096
H	2.38903022729881	1.25782317900279	15.13792627519037
H	3.68610185359488	0.53712818937660	14.16321023696572
H	2.66932873320587	1.80412746399952	13.47743845735100
N	3.68384043103995	3.87020697567689	14.73717331288047
N	5.53099761563197	5.38349902313257	12.71856630655618
N	5.76172055248574	2.68864672680762	11.92813734365489
C	6.89284884946624	6.95492749280203	11.39302791373745
H	6.05387261948818	7.61555734113927	11.17514601928680
H	7.55176051455027	6.92716640328022	10.52826542615182
H	7.43364430161424	7.40187816704282	12.22708539963946
N	4.11526915769240	2.48191221935360	14.83929826175408
C	6.42616443030837	5.56036516718844	11.74316610137793
C	6.95491533593239	4.52386492921672	10.97529277046360
H	7.65608433974078	4.80468821432693	10.20383315127001
C	6.54562510106806	3.18573444002486	10.98474135897343
C	6.97181534500047	2.35094189598727	9.79945397266701
H	6.95692256748394	1.28461567769846	10.00458440129720
H	7.97078797051198	2.64061322792756	9.47467211050192
H	6.29140476474098	2.53687954850190	8.96507999179115
C	4.91591000467843	6.56758162992793	13.23650144117037
C	5.51853095886060	7.29231426022540	14.27539352739347
C	4.95555788725351	8.51089555151498	14.65346618636577

H	5.41450073036775	9.08463325001318	15.44882210489152
C	3.82190213145287	9.00065975805288	14.02681839998152
H	3.40639826138539	9.95683470846189	14.31988523501576
C	3.19673718642589	8.24109773778708	13.04680564475774
H	2.28512122252614	8.60884795306454	12.59590661893121
C	3.71252156745584	7.00943118700154	12.65107481179203
C	6.73503670926074	6.76526672022770	15.01478638764859
H	7.08273862007419	5.88005008409100	14.48219512037491
C	6.33409788260398	6.32889230477492	16.42942241807652
H	7.18708064947946	5.89832851581877	16.95875462028372
H	5.54121691693486	5.58455215924701	16.38765899022019
H	5.96998017461869	7.18020497203930	17.00932189398577
C	7.89007432355984	7.77056303020577	15.07261256526119
H	7.61932027161331	8.65593469293798	15.65173404440372
H	8.18884602276147	8.10378474362355	14.07773367048204
H	8.76007188664023	7.31594362705103	15.55185681871707
C	2.98301732135540	6.15064768230797	11.63042613469223
H	3.19377883194185	5.11283445755627	11.89758013548956
C	1.46442023865095	6.33655747203988	11.68016010369894
H	1.08529747141289	6.25041589068744	12.69865310870934
H	0.97821389143456	5.57393376125815	11.06990917254326
H	1.16182554007746	7.30977903399350	11.28770097208658
C	3.48806776562425	6.36349326648897	10.19732627997167
H	3.36097345986398	7.40643123080086	9.89599904827447
H	2.92043437690936	5.73970676558855	9.50238652790841
H	4.53912263306767	6.10150330838140	10.09327071042792
C	5.26198719690053	1.35924428825522	11.78837800083842
C	5.90408222659733	0.29875043275297	12.45929118362977
C	5.35690670164605	-0.97740789153187	12.35057409353594
H	5.82916995774531	-1.80801570732543	12.85624283502355
C	4.20509990852412	-1.20694814768960	11.60828983920823
H	3.79347500776585	-2.20649303864107	11.54224225537821
C	3.58783515435769	-0.15446971131701	10.95311256583349
H	2.69145829906070	-0.33848357303749	10.37415624461614
C	4.10399604642649	1.13920038020724	11.02240392760869
C	7.15797721533030	0.54741769659881	13.28216615290419
H	7.01657988999363	1.51255268538971	13.77288195661288
C	8.42530034809096	0.66200420337909	12.42069437006372
H	8.55822008765044	-0.23599093811654	11.81238454584424
H	9.30150401567148	0.76568547256340	13.06483542689462
H	8.39880809330699	1.52320295761338	11.75975925217888
C	7.39725895774677	-0.50870456014276	14.36269935684972
H	6.51023625459337	-0.68737993529272	14.96675783228929
H	8.19963721941788	-0.18300600683597	15.02711695961594
H	7.70553329386008	-1.46190788157487	13.92746956680607
C	3.41128859113752	2.26238249561493	10.26669278475516

H	4.01730010869059	3.15996653938504	10.37361037353050
C	3.30158945464825	1.94935207868806	8.76847306422435
H	4.27242629017946	1.69281868131932	8.34274623757963
H	2.91021092100004	2.81557545337988	8.23065644926902
H	2.62546766284390	1.11127677426937	8.58622602637987
C	2.02765114575985	2.58189124430293	10.84550387790739
H	1.38174492811249	1.70101319467403	10.84140775125042
H	1.54217914175671	3.35840005542180	10.25108697881142
H	2.09988943438893	2.94722655363679	11.86964283977129
Si	2.32504102116362	4.38205747182769	15.68191904669149
Si	4.90603495884580	2.22696643221795	16.43748340183269
C	0.66420476790071	3.98098663151117	14.89016413268168
H	-0.16019875769797	4.33345627777872	15.51731151207583
H	0.53994666842542	2.90590192046436	14.74650465847224
H	0.56992904028034	4.45951897899997	13.91344922292561
C	2.41518273911583	6.21868836444392	15.99992165219142
H	2.48499612014645	6.78698613601801	15.07422501933251
H	3.28229096112285	6.47475631910752	16.60905760174169
H	1.51589990749097	6.54337252683140	16.53078920001690
C	2.48608857048652	3.43546882294605	17.31281580917240
H	1.93787162758861	3.96753680165004	18.09726461167225
H	2.00770003557908	2.45392424291084	17.22919904083437
C	3.97435324132725	3.27738041171799	17.68899460898083
H	4.09018044844195	2.80193308117150	18.67020164792806
H	4.44689571344032	4.25892394079031	17.77207900273684
C	6.71182237489334	2.69618723171681	16.34383966665376
H	6.85271502751317	3.59890722830861	15.74887235674445
H	7.31523874107370	1.90072174439266	15.90839350217053
H	7.09038436067400	2.89572373335637	17.34988913234675
C	4.68635536017055	0.42826425984774	16.92407513051857
H	5.32271552230811	0.20454982380242	17.78438132046688
H	4.94352581440994	-0.26866188335453	16.12804635610297
H	3.65210547091606	0.23433572961757	17.21723941643792

Atomic coordinates of the optimized structure of **10** (η^2, $S = 1$) second conformer

Fe	4.97635922348345	3.82155158703897	13.39190277746162
N	5.44508494542444	5.49690213799393	12.52799794822107
N	5.83532044186108	2.77053841915609	11.99632686165594
C	6.77986089321531	7.04776327818964	11.15206921399567
H	5.91976561590585	7.69932358574130	11.00188310511451
H	7.35628263982505	7.00441696286509	10.23060342759139
H	7.39909721817081	7.51420479160027	11.91984538088661
C	6.35687148748677	5.65974539892655	11.57214325606526
C	6.94367213274895	4.59122829014535	10.89535868049369
H	7.64413727554715	4.83935357061472	10.11187641757060
C	6.60892585109944	3.24050274445600	11.02489991562809

C	7.12628729674412	2.33233751366133	9.93173225963909
H	8.14398212038471	2.61868258442013	9.66660007120757
H	6.51201675263578	2.45072309238651	9.03712256624609
H	7.11324098429972	1.28293377498018	10.20801978355375
C	4.84934809073922	6.66330020129222	13.08993545147033
C	5.53434230454046	7.39532359637816	14.07621226074244
C	4.93460405957716	8.54792872615004	14.57924589046421
H	5.44229002904918	9.12574955172422	15.34033962323066
C	3.69544245855892	8.96862931473259	14.12169132949040
H	3.24740157524370	9.87078251141036	14.51937506010225
C	3.01809250548318	8.22025007360233	13.16811689418799
H	2.04132635926030	8.54663717786737	12.83815257339412
C	3.57147530747138	7.05430285703855	12.64457090172885
C	6.88214799542429	6.93543274120149	14.60920109202201
H	7.31204423195695	6.25075246738125	13.87957871224964
C	6.71636712273718	6.14214655187827	15.91211538526872
H	7.68856698907951	5.79823536960310	16.27299222452210
H	6.08370795629392	5.26762574980568	15.75712925942475
H	6.26369321749336	6.76127461123491	16.69033827963338
C	7.87554578048635	8.08530623581601	14.80058487987795
H	7.57030074067207	8.75481214362273	15.60721878838003
H	7.97922036976161	8.68176725166107	13.89213615289506
H	8.85842261342560	7.68710801761539	15.06070926345902
C	2.82232298562188	6.20956884080170	11.62785067268763
H	3.07010160186811	5.16996785136849	11.85106813597612
C	1.30304434193762	6.35152816749638	11.72469805765451
H	0.95408028296225	6.22957423383510	12.75010429619648
H	0.82175801183125	5.59078445768396	11.10771381466817
H	0.96274703826021	7.32596074802331	11.36712619532102
C	3.28108183460923	6.47911227331106	10.18786006792337
H	3.12586933342730	7.52809728804194	9.92329291530712
H	2.70587854872157	5.86555835828432	9.49051117183018
H	4.33323940160766	6.24131752698993	10.04737602408395
C	5.34681075610238	1.43128177505135	11.89191374331342
C	5.92758786152908	0.40299553595571	12.66193641734212
C	5.35961741950942	-0.86702453779176	12.59426898677784
H	5.77584775891950	-1.67203048711321	13.18232245467862
C	4.26008571852749	-1.12650294199025	11.78597265700745
H	3.83117517424993	-2.12052553038980	11.75785057424366
C	3.72590801703419	-0.11436735131822	11.00702642422326
H	2.88035038712772	-0.32474314851847	10.36428139142232
C	4.26072438497319	1.17296101673121	11.03388451307160
C	7.14337413347840	0.67293083719412	13.53528401563876
H	6.93202521820943	1.59905580416144	14.07462038723755
C	8.42628416449474	0.90997675851720	12.72154853501964
H	8.62469639356339	0.06629590883975	12.05605038309073

H	9.27650675965835	1.01009237404967	13.39995251347676
H	8.37403893952482	1.81524332700366	12.12525657622240
C	7.42400503760142	-0.42974779909716	14.55787696011317
H	6.55887293960712	-0.65823738823668	15.17671068711785
H	8.23500189238497	-0.11832028349927	15.21787456386227
H	7.74443898049014	-1.35167802746775	14.06677376112613
C	3.66080266392911	2.24135139876309	10.12950062011491
H	4.27806485920266	3.13462851641867	10.20189330011546
C	3.65653654699396	1.79681818927768	8.66028260339847
H	4.64454006323341	1.46709119748609	8.33746518686753
H	3.34510390979845	2.62406699059796	8.01878293254376
H	2.96079800043939	0.97100189059039	8.49756706762682
C	2.24574076791878	2.64422151199078	10.55951319143454
H	1.57436520473937	1.78265598851813	10.57394511869484
H	1.83733074795543	3.38018751171798	9.86352887737934
H	2.24610764054891	3.08758906622839	11.55245710668006
Si	2.25253790115546	4.21514676103174	15.78396050084034
Si	4.81355235175391	2.07911064819160	16.41850660766653
C	0.47414307063990	4.36824167443977	15.21337229188344
H	-0.06015684365227	5.05761901720777	15.87231684843604
H	-0.04346158193752	3.40786458489879	15.24478724062655
H	0.40378423505246	4.75812821556079	14.19696039209378
C	2.99885501978778	5.88980249096765	16.07115799904362
H	2.97225596317555	6.51135821977484	15.17942290184889
H	4.03072550018812	5.82358649917808	16.40707233403390
H	2.42367069125731	6.39431436681229	16.85290332432134
C	2.35030549333730	3.20216073555510	17.36575934641783
H	1.80422733022297	3.72452302829519	18.15974275007806
H	1.81972836388806	2.25605942945308	17.21413042608125
C	3.81461452600263	2.93586222042933	17.77543811948848
H	3.86224868921390	2.33470294355722	18.69084623831395
H	4.31187039068303	3.88322249354539	18.00718710676577
C	6.62925034257541	2.24395814848255	16.84980927166248
H	6.81605643135904	1.76854518635842	17.81686836543378
H	7.29633477833678	1.78704525384528	16.12240438286499
H	6.89539252538506	3.29874806863593	16.93618421692909
C	4.26838908246438	0.27329977632920	16.38387116874903
H	4.76606574743497	-0.30557926159459	17.16698729817375
H	4.48243663737767	-0.19346005400827	15.42238969118165
H	3.19130875387671	0.19799487656758	16.55221218183594
N	4.51702552197916	2.92738397079163	14.93465744811885
N	3.15144384859603	3.28917499505659	14.54651940364726
C	2.40254699499802	2.12145325610703	14.04313160014452
H	1.50483500133403	2.45440392533870	13.52509393936350
H	2.10493338782147	1.44564024164740	14.85190688239765
H	3.02436953701102	1.56467755584307	13.34813113218634

Atomic coordinates of the optimized structure of a methyl radical

C	-5.19615443433622	3.47387760441552	0.23495246789029
H	-4.17790844080107	3.34433709136090	-0.09972390865425
H	-5.70528587836262	4.41071768997067	0.06541336385098
H	-5.70526124650007	2.66692761425290	0.74017807691298

Atomic coordinates of the optimized structure of ethane

C	-8.33239840042945	4.48041122265558	0.00001298888531
C	-8.32112908821704	2.95372877256664	-0.00001302978574
H	-8.62126132258371	4.87480328481269	-0.97676229135219
H	-7.34640097296624	4.88415418533055	0.24067665268774
H	-9.03814424627525	4.87177534503580	0.73610015369898
H	-7.61538356036049	2.56236445867380	-0.73610037325834
H	-9.30712662023826	2.54998588563879	-0.24067628536085
H	-8.03226578892953	2.55933684528614	0.97676218448510

Atomic coordinates of the optimized structure of **Me-TS** (*S* = 1)

Fe	-0.05825614736244	0.22021562070150	-0.39663873730942
Si	-1.91567963356708	-2.86358207029337	1.95733288564635
Si	-1.56139285290973	-0.32973826239262	3.49818518093611
N	-0.78196101717620	-0.56580877064471	0.91258847780003
N	-1.39398613653665	-1.16846666541284	1.94544833841931
N	0.83448821763345	-0.50035668008818	-1.94904791112473
N	0.54756565659088	2.02863700808693	-0.73863571084567
C	-1.95093280734469	-3.07409490363397	3.82857530424939
H	-2.58423635943788	-3.90497803846139	4.15064505474569
H	-0.93453748309349	-3.30362669092075	4.16368614956891
C	-2.41348596792461	-1.72477036843419	4.43916262988777
H	-2.21815464636923	-1.67617869186033	5.51393961833588
H	-3.49532848389973	-1.61349922717986	4.31564978193707
C	-0.68942755301477	-3.93897534050488	1.06156372676806
H	-0.56074621810819	-3.61297333482529	0.02891950376686
H	-1.03176880094932	-4.97682068279679	1.04443358412031
H	0.28757656282630	-3.90859819855003	1.54467477898161
C	-3.63285983381017	-3.11874505291023	1.24394360463686
H	-4.34593891659959	-2.41022722584408	1.67041491783793
H	-3.97888061694182	-4.13014533107152	1.47735652726198
H	-3.65189481203872	-3.00647943207243	0.15912253730746
C	0.10224147703288	0.12961765200562	4.21184338748540
H	0.79898130976642	-0.71045338332604	4.19269400778169
H	-0.01309620667142	0.45829752747843	5.24847946875264
H	0.54268790018600	0.95178693453954	3.64856203447716
C	-2.59557157163737	1.21908282964342	3.35612790379874
H	-2.10072849194102	1.95338084711678	2.71838354551235
H	-2.72568756647317	1.67539160311500	4.34153183142084
H	-3.58422450298600	1.00800892820255	2.94462378030700

C	-2.29908921236327	0.04615601299165	-0.17191965297493
H	-2.75704588306769	0.69251600695175	0.56350757045377
H	-2.28863059841238	0.52002307923566	-1.15409791626731
H	-2.78380184389420	-0.91713606342793	-0.22849088127613
C	1.77897214784297	3.85389188863364	-1.84608388808455
H	1.17086144637465	4.54706499388487	-1.27188837224434
H	1.82595736531740	4.18846244077218	-2.88174453027164
H	2.79285082494626	3.89526205490487	-1.44080323706927
C	1.26060319407122	2.43678398801641	-1.77967962924188
C	1.63947559422671	1.58529314898080	-2.82246375859274
H	2.18764557063201	2.03802974675974	-3.63556573888454
C	1.49442763586983	0.19516741631213	-2.86854649717916
C	2.20145057681930	-0.51888397662687	-3.99696247783425
H	3.18029878542360	-0.86595351705478	-3.65727070580694
H	2.35904440404203	0.15915612561580	-4.83366156075181
H	1.65630008242400	-1.39359228801542	-4.34188576004656
C	0.36754391453856	2.88764951070082	0.38939195539911
C	1.40176375379442	2.98789201300377	1.33979421163696
C	1.17869290801279	3.74669318842847	2.48647114286813
H	1.95599152102882	3.81823907066668	3.23653710582230
C	-0.02110615818771	4.41387785762562	2.68250154410639
H	-0.17806635149518	4.99873080739015	3.58034307189018
C	-1.01703867876329	4.33641203003955	1.72006010406147
H	-1.94488107906407	4.87061646506686	1.87508522811369
C	-0.84785163970079	3.57541790781738	0.56480802263010
C	2.73457780184289	2.28343527734665	1.14502638591361
H	2.78098481625956	1.93590389662303	0.11478649545953
C	2.84839778565428	1.04315131060178	2.03614648307028
H	2.82251380525211	1.31705263764045	3.09275438050427
H	3.78951369677073	0.52307063633607	1.84442091014659
H	2.03055195478075	0.35056482309938	1.84180203460817
C	3.92512465906355	3.22353265223978	1.36471997955354
H	3.84302114135384	4.12104815851582	0.74907469010309
H	4.85635245869458	2.71497503007610	1.10580262475169
H	4.00170800439732	3.53948415189179	2.40704505134383
C	-1.93787767809589	3.52883362058420	-0.49171417516689
H	-1.79679707879247	2.60616367308795	-1.05122082024575
C	-3.35274744540556	3.51703120125596	0.09458596115748
H	-3.46222165723379	2.76676357666558	0.87649320360779
H	-4.07730555742995	3.29696928913569	-0.69184715641778
H	-3.61913715679637	4.48646704469736	0.52128272281714
C	-1.80150079922534	4.69041567741793	-1.48773603103259
H	-1.86885547808039	5.65072574116946	-0.97046520296805
H	-2.60344716176426	4.64758244183150	-2.22865446528577
H	-0.85232913970626	4.65758580786121	-2.01951508910974
C	0.80726028819258	-1.92602046875921	-2.05127923579265

C	1.84677288659770	-2.68096049474615	-1.48080866806439
C	1.82493960614428	-4.06556883975045	-1.63388525416984
H	2.61946554502347	-4.66032135736052	-1.20094973812504
C	0.79912106739204	-4.69580330398717	-2.32121761911087
H	0.80049013415981	-5.77290546432532	-2.43364069796655
C	-0.24072194583483	-3.94198117268909	-2.84495853517099
H	-1.04902382822301	-4.44061227106688	-3.36441737987322
C	-0.26182952316812	-2.55438354891214	-2.71551412584585
C	2.95576387277806	-2.03085199763399	-0.67348997246476
H	2.84033118534290	-0.95106581034763	-0.75905567049255
C	2.81239753990495	-2.39225443297345	0.81066326696621
H	1.84588336326055	-2.06444493279945	1.19326527842734
H	3.59562676354801	-1.91196121949330	1.39995184588771
H	2.89097122261105	-3.47187444745825	0.95660310944910
C	4.35133922721623	-2.39365365099060	-1.19230119416266
H	4.55881266125260	-3.45909362017275	-1.07180213228441
H	5.11454002369972	-1.84288138717467	-0.63789111640032
H	4.45858331699831	-2.15052964943723	-2.25066389570256
C	-1.41168740274791	-1.75420103155294	-3.30039684021557
H	-1.35120699588576	-0.75121275408011	-2.88084025833279
C	-2.77372258857117	-2.34047689130859	-2.91579767634528
H	-2.95808576006852	-3.29404295491374	-3.41523397585347
H	-3.57404390433318	-1.65602550217631	-3.20421760428836
H	-2.83948599949606	-2.50791925940085	-1.84165023905103
C	-1.29995762574598	-1.62255854353030	-4.82526811053994
H	-0.38040270567687	-1.11683577728686	-5.11860688134920
H	-2.14099252773981	-1.04740727859282	-5.21986550676625
H	-1.31079764582895	-2.60657909349706	-5.30052070809185

Atomic coordinates of the optimized structure of **Me-TS** ($S = 2$)

Fe	-0.13952489624654	0.30779339989887	-0.34508678122695
Si	-1.91426668865985	-3.04616617944092	2.01616544957406
Si	-1.57904144832144	-0.40556939768536	3.37052752167793
N	-0.87440826748396	-0.91026939194710	0.73491517093156
N	-1.42068330974729	-1.34803981824480	1.87883206575844
N	0.77278678932305	-0.49862437687190	-1.97565007447175
N	0.57161859430473	2.14863533351988	-0.68244803229621
C	-1.99242981941057	-3.12438839770341	3.89809962708338
H	-2.64536543124587	-3.91875182847841	4.26927455190204
H	-0.98614588329508	-3.34921050317147	4.26599667083577
C	-2.43631771634373	-1.72723053928172	4.40949716235940
H	-2.24333233684356	-1.60619497560444	5.47903334032124
H	-3.51687792034831	-1.61188249250208	4.27618523076098
C	-0.64555030376844	-4.17350976188045	1.24699994516251
H	-0.48512548462085	-3.91762893788914	0.19888227092726
H	-0.97601678531682	-5.21444625205253	1.29433911428849

H	0.31407198755587	-4.09480807159007	1.76002786945969
C	-3.59331580441353	-3.35607011735963	1.23968379961511
H	-4.34905805758450	-2.68168571032670	1.64816924980623
H	-3.91472594870859	-4.38357808564539	1.43289627225418
H	-3.56357679082767	-3.21722160032143	0.15725268698491
C	0.09115106195860	0.10018716895638	4.04128204896599
H	0.78639262549267	-0.74069539275988	4.07619675324431
H	-0.01393084577402	0.50400145921673	5.05198524141572
H	0.52968972437139	0.87962445339324	3.41866859907058
C	-2.61340961081134	1.13519423035200	3.15180989757059
H	-2.09852890407059	1.86178948122658	2.52215483025499
H	-2.78308138275975	1.60818944577871	4.12330515471215
H	-3.58351739560416	0.90962490463952	2.70643381525127
C	-2.38320641332192	0.04809598857656	-0.24302284496074
H	-2.66930537469647	0.91577629147422	0.34142268076615
H	-2.42045037424461	0.25901715133958	-1.31338036308779
H	-3.00661259408245	-0.80318413150872	-0.01212565775669
C	1.85788129549981	3.89029320420467	-1.84773171345914
H	1.28950292663546	4.61366088226757	-1.26900513520212
H	1.89665648490374	4.20974039781235	-2.88815251476680
H	2.88060753109453	3.89397991241277	-1.46317926660813
C	1.28175328766526	2.49690774131035	-1.74675601953103
C	1.59879915686472	1.62080398303132	-2.79300334286459
H	2.14716008963412	2.06279501297552	-3.61195755033649
C	1.43255367243036	0.22661066925198	-2.86721607796355
C	2.13543243466750	-0.45715583279094	-4.01763034740973
H	3.10318624202589	-0.83611792413077	-3.67982285460627
H	2.31394652454031	0.24366728853831	-4.83057897523935
H	1.57401306298176	-1.30976338734917	-4.39077447804524
C	0.43124804811257	3.01315481838536	0.44058945349524
C	1.48087045073045	3.10607121309975	1.37528171457633
C	1.26994288888273	3.84294046735829	2.53869604824904
H	2.05873197855123	3.90861802461108	3.27688379761562
C	0.06579373866729	4.49086225672631	2.76837585902463
H	-0.08178311898420	5.05663475634691	3.67982340482331
C	-0.94834639263596	4.41791161108836	1.82393880547398
H	-1.88027821847701	4.93463094430953	2.00886517336766
C	-0.79186560612519	3.68219969070324	0.65140812124706
C	2.80966351598562	2.40404726587272	1.14697089387407
H	2.86249088754422	2.12007808516192	0.09811908262440
C	2.90119640869653	1.10683416368326	1.95572384713103
H	2.85141857490853	1.30785993962181	3.02766740769334
H	3.84279471560451	0.59408365719160	1.74833992967819
H	2.08704014567394	0.42770529249892	1.70145463773821
C	4.00927613476301	3.31326032140980	1.43167738832077
H	3.94151706674641	4.24709481205635	0.87043581101764

H	4.93521407188533	2.80937762479608	1.14643983195916
H	4.08526045485593	3.56389350216155	2.49149302754452
C	-1.89560808834593	3.63362855694929	-0.39199178217145
H	-1.79914077141090	2.68154246931180	-0.91365736254716
C	-3.30557360395902	3.70754224186666	0.19921931173560
H	-3.44797638654242	2.98931486565901	1.00606280137940
H	-4.04144460152837	3.49431373428937	-0.57822683229814
H	-3.52601857181377	4.70236519594492	0.59251967405868
C	-1.71658375825174	4.74027883947939	-1.44260847729735
H	-1.73789696548619	5.72529144627074	-0.96983212532648
H	-2.52541372727092	4.69768613193219	-2.17581895908536
H	-0.77424092238422	4.64090978974050	-1.97811957560716
C	0.77407092245563	-1.92120938948392	-2.08813668287217
C	1.80518259326426	-2.66157318661884	-1.48254419855809
C	1.79635993837660	-4.04854638195495	-1.61104070318729
H	2.58449726510832	-4.62967188499195	-1.14878291545374
C	0.78969600509865	-4.69966709984838	-2.30719209344879
H	0.79956887602430	-5.77873977592329	-2.39817718402777
C	-0.24128134618467	-3.96122183466803	-2.86738122126152
H	-1.03564111874602	-4.47372051676197	-3.39528549233638
C	-0.27365356512842	-2.57152155502607	-2.76410086991864
C	2.89059043688402	-1.99467152590802	-0.65660004957809
H	2.76316426570045	-0.91649855125597	-0.74668096281699
C	2.72505977950592	-2.35508421115708	0.82571571236585
H	1.74323470886308	-2.04860208730423	1.18737936076361
H	3.48685993387665	-1.85891543561986	1.42989073548039
H	2.82188377233154	-3.43249360103865	0.97615003963528
C	4.29934548342531	-2.34004799806408	-1.15120087794093
H	4.51502869044800	-3.40408517713723	-1.03260172628076
H	5.04754160775293	-1.78480446417651	-0.58087230139053
H	4.42224277565463	-2.09044492605843	-2.20650833486360
C	-1.42490758275371	-1.80075503704630	-3.38481592949802
H	-1.33602900694029	-0.76452867392893	-3.06084332965296
C	-2.78045592200559	-2.32762418648286	-2.90157590882010
H	-2.96838831354622	-3.33862093356654	-3.26859808548309
H	-3.58813854601708	-1.68739998078100	-3.26298822176813
H	-2.82325777967702	-2.34988991695963	-1.81371178926064
C	-1.36100436890376	-1.81001922227558	-4.91798768895865
H	-0.43534704586122	-1.36465617211051	-5.28325903909874
H	-2.19598223095399	-1.24366812358441	-5.33732650938349
H	-1.41936827984255	-2.83085916243419	-5.30296563180325

Atomic coordinates of the optimized structure of **11** ($S = 1$)

Fe	6.00816707817306	13.61534843762986	4.51451119348569
Si	5.29201392975454	16.41302135596024	2.15909482628918
Si	2.70680748230451	16.03517232813480	3.51954388512250

N	5.41322292025524	11.86125766968679	5.00328384678689
N	7.88361627389119	13.15817557952881	4.66614924720342
N	5.17636374815640	15.24403103107936	4.65553828011620
N	4.45208910566239	15.89359841227904	3.59858394631727
C	4.06492136232324	11.53689502638771	4.65506822643738
C	8.33564451881191	12.15242547384580	5.41092399851924
C	8.80715698330349	13.95359969417305	3.91558725252040
C	6.15417672521619	10.94852085186861	5.62479592814712
C	3.04764029928466	11.58120556046026	5.62457410668698
C	1.47556116618094	10.79419903211499	3.95952879965439
H	0.46958236785225	10.49652704770734	3.69119983203166
C	1.75926785080464	11.20038441906348	5.25372651288304
H	0.96403235309189	11.22976886251998	5.98737130191696
C	9.30329869552588	15.16484217547475	4.42722262211980
C	3.77832902913094	11.17913426318393	3.32318991706995
C	7.50985067973598	11.13892331149325	5.90483255720962
H	7.99662054375308	10.36118213622342	6.47431741338502
C	9.17957371942926	13.50631115470511	2.62835925571974
C	10.16015411007435	15.92357716310901	3.62991524687151
H	10.54918385209000	16.85934290751459	4.01066563621791
C	4.86781146863785	11.19018530276074	2.26616536591673
H	5.58559960740544	11.95909019632029	2.56127315343700
C	3.30664627245503	12.09849089636086	7.02741095037578
H	4.38489071541797	12.13587140856579	7.17688193706009
C	8.66587574405632	12.18707349074753	2.07288232631749
H	7.72440614090925	11.97376735106157	2.57756153448993
C	2.47839342052139	10.79837264377878	3.00151036653696
H	2.24138738434961	10.51009025611765	1.98655990017619
C	9.79938411426040	12.05386986617605	5.77316738594947
H	10.44130543744484	12.56841294552718	5.06340343436027
H	10.10930798581215	11.01251121431342	5.84478005073195
H	9.95496449270168	12.51024143424449	6.75357307910299
C	10.04670914451983	14.29354443686252	1.87617124222928
H	10.34099744174264	13.96759207018417	0.88769973778338
C	5.54623661077343	9.63049968005296	6.03900925745793
H	4.87217454519210	9.75846827997242	6.88652283616234
H	6.32518436727995	8.92790926276400	6.32662188149857
H	4.95899158743220	9.19545164660409	5.23047286422200
C	10.53245448847328	15.49751988016535	2.36591201551821
H	11.20074403271060	16.09975669154264	1.76302933398629
C	4.35822601863974	11.56218527052471	0.87300121109215
H	3.72785547574542	10.77965833997933	0.44612544250102
H	5.20428349478609	11.70457929478012	0.19906642007957
H	3.78117547864685	12.48800209166080	0.89538656173680
C	8.39007301251646	12.22442860156510	0.56681256577002
H	7.73504785280557	13.05116271330121	0.29790646874974

H	7.90871136429110	11.29421530625372	0.25960157050902
H	9.31192696020428	12.31607022068614	-0.01090977475890
C	8.97475487017161	15.64549938227820	5.82819110024740
H	8.21359198562173	14.97833565992500	6.23320614976154
C	5.62728991282645	9.85710000112556	2.22627347941161
H	6.10905569935686	9.64034604881357	3.17889422400568
H	6.40139736530176	9.88200045721847	1.45696011510764
H	4.94397369838357	9.03641469017952	1.99477610509516
C	1.89627439572393	14.39257026860355	3.13755430889286
H	2.24602592269570	14.00124258142622	2.18010727065848
H	0.80834563428744	14.48972679225692	3.08727623781714
H	2.12966779287365	13.64753969370572	3.89855222708694
C	1.88052149488322	16.77265904665708	5.03560532607594
H	1.85121305163885	16.08355764914854	5.88043994359850
H	0.84578068655673	17.02035305128224	4.77863294213344
H	2.37663268151114	17.69106205949427	5.35473009090448
C	2.78494396048812	13.53591948069655	7.14374946174589
H	3.23125788337711	14.16067380962836	6.37358288315587
H	3.03101553432318	13.96161714331595	8.11937407614423
H	1.69986329060633	13.56594221973158	7.02018416640513
C	8.40919524196349	17.06859348665063	5.83379262921063
H	9.16104291164205	17.79399688389181	5.51613531196560
H	8.08886760403757	17.34369414258721	6.84094963443479
H	7.55358546555135	17.15535850771292	5.16971071347260
C	2.70950097991190	11.21292760416351	8.12500384703250
H	1.61820714518195	11.21829195272559	8.09593698488905
H	3.01318352857905	11.58063817567960	9.10743167039843
H	3.03989560321439	10.17683551825492	8.03358897015634
C	3.80244720651950	16.93197695632599	1.11968986087858
H	4.02303982798655	17.77373899894122	0.45755413725616
H	3.54959475422675	16.08789537852427	0.46994802834433
C	2.61415122564214	17.23302754213750	2.06738535537244
H	1.65354916798402	17.19189800973290	1.54601229954243
H	2.70725468591561	18.24665809708756	2.47027040236997
C	9.62512886489740	11.02976690837645	2.38835765930472
H	10.60310381214074	11.21015579926409	1.93522667626504
H	9.23031177100427	10.09432926881375	1.98548226937671
H	9.76777894087900	10.89862848234250	3.45828374481416
C	6.51316876358546	17.79927087360707	2.47427556386753
H	6.05811039886159	18.62369406048733	3.02695716156817
H	6.89073655032945	18.19065030068360	1.52533920782466
H	7.37078890349412	17.43432786926460	3.04165923734470
C	10.20742048835101	15.57549135632026	6.74189681934273
H	10.64193705968382	14.57721475049465	6.76137200507869
H	9.93867785664209	15.85252506199067	7.76400121610376
H	10.97941330113281	16.26809111461143	6.39869852735355

C	6.20974317298872	15.00866851472015	1.33653198253349
H	7.05396918653011	14.67494836140457	1.93799970585613
H	6.61001627470544	15.32752747261831	0.36984792776491
H	5.54813759635739	14.15868924462605	1.16448084536885
C	5.12410036605902	16.04564013504250	5.86538847692324
H	4.11608889592684	16.10487295116285	6.28577175096049
H	5.77797777523616	15.60070352540725	6.61716377193040
H	5.45542913067069	17.07077754383484	5.67099455814047

Atomic coordinates of the optimized structure of **11** ($S = 2$)

Fe	6.02336145456005	13.70184105121247	4.60249956055539
Si	5.20661783283864	16.53534139866447	2.20188477341776
Si	2.63016778678095	15.97478211358793	3.51262619936848
N	5.36239721143224	11.83417201105003	4.99146380798938
N	7.97177518991258	13.15837194721234	4.68181910817908
N	5.13086917569571	15.36674595569017	4.68767750531268
N	4.37298555144437	15.91636771541386	3.59976389552383
C	4.02409079541376	11.48654867896710	4.64363949235178
C	8.35806527214815	12.11795452547974	5.41324524592367
C	8.93270585790466	13.90627648163998	3.93570954199065
C	6.12464998727150	10.94436643365031	5.61721890454182
C	3.00292939902058	11.53372724202123	5.60927041094389
C	1.44695248455641	10.72380435502999	3.93997965676178
H	0.44479525540327	10.41585881209087	3.66910445045364
C	1.72068959467826	11.13861913951432	5.23467555414678
H	0.92066472486096	11.16595426183599	5.96275923183605
C	9.46697777501215	15.10195255702298	4.44320656084957
C	3.74808184897786	11.12752964967543	3.31142123078729
C	7.48495429511087	11.13713554457380	5.90187555388599
H	7.95177298501929	10.35076384849900	6.47576306185014
C	9.28679913639732	13.44419486769587	2.64965849440014
C	10.36904435296695	15.81627671088016	3.65535342740903
H	10.79192418973585	16.73755338192322	4.03556003255227
C	4.84431444336717	11.15401855699849	2.26093548551476
H	5.56932527728034	11.90578704449869	2.57899157834361
C	3.26215042491863	12.07455075092304	7.00427064455942
H	4.33509229707852	12.03105382486777	7.18751127657530
C	8.69801356221501	12.16439555408337	2.07889470418946
H	7.76835244707838	11.97096201891615	2.61314251192494
C	2.45252583896057	10.73450399140540	2.98538367074917
H	2.22187323829281	10.44302247777341	1.96964867885749
C	9.81507676575106	11.94093802610714	5.77435286645369
H	10.48018649847531	12.39407451412389	5.04448232197499
H	10.06499813163710	10.88745592426405	5.88415743267381
H	9.99919667049540	12.42761803137910	6.73553465339753
C	10.20095184598749	14.18550377044597	1.90669553069326

H	10.48767135120966	13.84414322813384	0.92104331900820
C	5.53298367479229	9.62237345312900	6.04624903660112
H	4.84478753469720	9.75983022796785	6.88174759670857
H	6.31472824414134	8.93286613908322	6.35527082276528
H	4.95939780223572	9.17143407813348	5.23685454017608
C	10.74200119099388	15.36388199842362	2.40048179282244
H	11.44780227818806	15.92955098938437	1.80505200649146
C	4.34687994152910	11.57214996071869	0.87618726053756
H	3.69430641373467	10.81912600515166	0.43002094348993
H	5.19727718413261	11.70648059190175	0.20579578417593
H	3.79716432698914	12.51373054528724	0.92099794643352
C	8.36079948465550	12.27162406078696	0.58882074892528
H	7.72708934987488	13.13374406988376	0.38621464595954
H	7.83229718981434	11.37341457124761	0.26410879558733
H	9.25936859791203	12.35734283475669	-0.02528883393272
C	9.11150683704543	15.61966134730510	5.82368692622156
H	8.33055450505048	14.97382604953506	6.22691325112937
C	5.58859524681569	9.81378462268281	2.19253089711706
H	6.05540619110007	9.56590979616270	3.14564908059626
H	6.37310958204294	9.84944521424060	1.43405776583525
H	4.89936924969346	9.00684267684785	1.93198650675516
C	1.86070481776206	14.29378756009114	3.22467749611357
H	2.22867925733199	13.85236376607504	2.29666869881508
H	0.77189742725412	14.36862740812233	3.15514530645543
H	2.09391866648383	13.59917263503859	4.03086696222365
C	1.79746133459212	16.76264942557739	5.00026860860242
H	1.87269351614262	16.13722623491048	5.89116526607962
H	0.73403093410896	16.90692344221079	4.78638378507729
H	2.23225162081339	17.73777378463881	5.22819632521296
C	2.85890922246087	13.55437282612986	7.06662879902019
H	3.39315381483974	14.13057914117069	6.31135481263450
H	3.08972854195139	13.97565023451185	8.04795783990962
H	1.78709605406061	13.66892082354081	6.88875350626171
C	8.55992114586817	17.04719173283219	5.76676555257269
H	9.32592224190607	17.75440675335348	5.44222684640628
H	8.21305219072483	17.36141650293936	6.75322243486490
H	7.72194772892410	17.11329054348593	5.07617574489326
C	2.57364613881951	11.26962292843481	8.10930371711788
H	1.48693741000221	11.36097409707375	8.06021846566101
H	2.88854220413620	11.63637989875123	9.08841430121455
H	2.82347984970021	10.20861647911666	8.04659350366629
C	3.71361148809417	16.78562362940448	1.07493165354734
H	3.86271003261553	17.56755493358764	0.32512630855325
H	3.55481865188024	15.85285923858838	0.52344596930121
C	2.48722265849562	17.07068096054293	1.98071257970673
H	1.54255852112431	16.92847783564756	1.44769853398015

H	2.50491997608244	18.11494778956896	2.30924725226158
C	9.62081421146845	10.96124314128104	2.32127495063571
H	10.58601295620067	11.11433329314556	1.83219679460028
H	9.17257409574430	10.05238573657673	1.91360786684820
H	9.80347096695113	10.79765833593156	3.38166639871015
C	6.13517372759485	18.13517186579873	2.52396456532426
H	5.46066538883026	18.91490587531794	2.88470744341649
H	6.61272409785139	18.49466785810414	1.60777267839752
H	6.91970024219063	17.99373240406294	3.26955166571857
C	10.31454738351120	15.55216696799308	6.77464888779447
H	10.72042838266547	14.54280635499137	6.83923004812979
H	10.02457590109954	15.86955553157993	7.77894918283176
H	11.11473061354662	16.21193297063506	6.43168797258406
C	6.41268358514573	15.28187590114042	1.51204982279766
H	7.29189809111538	15.16778295474779	2.14675670561266
H	6.76625710604687	15.58973880023453	0.52403978903415
H	5.93504737857912	14.30533987308956	1.40849381372258
C	5.17221776964215	16.26087816865446	5.82366889016021
H	4.19500797937853	16.37269676622986	6.31122551177619
H	5.86688235638280	15.86437704962646	6.56837401515964
H	5.51154631754832	17.26600348762561	5.54223684924803

References

(1) McWilliams, S. F.; Rodgers, K. R.; Lukat-Rodgers, G.; Mercado, B. Q.; Grubel, K.; Holland, P. L. *Inorg. Chem.* **2016,** *55*, 2960-2968.
(2) McWilliams, S. F.; Broere, D. L. J.; Halliday, C. J. V.; Bhutto, S. M.; Mercado, B. Q.; Holland, P. L. *Nature* **2020,** *584*, 221-226.
(3) Sciarone, T. J. J.; Meetsma, A.; Hessen, B. *Inorg. Chim. Acta* **2006,** *359*, 1815-1825.
(4) MacLeod, K. C.; DiMucci, I. M.; Zovinka, E. P.; McWilliams, S. F.; Mercado, B. Q.; Lancaster, K. M.; Holland, P. L. *Organometallics* **2019,** *38*, 4224-4232.
(5) McWilliams, S. F.; Bill, E.; Lukat-Rodgers, G.; Rodgers, K. R.; Mercado, B. Q.; Holland, P. L. *J. Am. Chem. Soc.* **2018,** *140*, 8586-8598.
(6) Castillo, M.; Metta-Magaña, A. J.; Fortier, S. *New J. Chem.* **2016,** *40*, 1923-1926.
(7) Stoll, S.; Schweiger, A. *J. Magn. Reson.* **2006,** *178*, 42-55.
(8) Prescher, C.; McCammon, C.; Dubrovinsky, L. *J. Appl. Cryst.* **2012,** *45*, 329-331.
(9) Baker, T.; Lewis, S. E. *Synth. Comm.* **2010,** *40*, 2747-2752.
(10) Field, L. D.; Hazari, N.; Li, H. L. *Inorg. Chem.* **2015,** *54*, 4768-4776.
(11) Battino, R.; Rettich, T. R.; Tominaga, T. *J. Phys. Chem. Ref. Data* **1984,** *13*, 563-600.
(12) Sheldrick, G. *Acta Cryst. C* **2015,** *71*, 3-8.
(13) Spek, A. L. *Acta Cryst. C* **2015,** *71*, 9-18.
(14) McWilliams, S. F.; Brennan-Wydra, E.; MacLeod, K. C.; Holland, P. L. *ACS Omega* **2017,** *2*, 2594-2606.
(15) Bhutto, S. M.; Mercado, B. Q.; Holland, P. L. *Inorg. Chem.* **2023,** *62*, 9335-9342.
(16) Weigend, F.; Ahlrichs, R. *Phys. Chem. Chem. Phys.* **2005,** *7*, 3297-3305.
(17) van Lenthe, E.; Baerends, E. J.; Snijders, J. G. *J. Chem. Phys.* **1993,** *99*, 4597-4610.
(18) Izsák, R.; Neese, F. *J. Chem. Phys.* **2011,** *135*, 144105.
(19) Angeli, C.; Cimiraglia, R.; Malrieu, J.-P. *Chem. Phys. Lett.* **2001,** *350*, 297-305.
